# The interaction networks of small rubber particle proteins in the latex of *Taraxacum koksaghyz* reveal diverse functions in stress responses and secondary metabolism

**DOI:** 10.3389/fpls.2024.1498737

**Published:** 2024-12-13

**Authors:** Silva Melissa Wolters, Natalie Laibach, Jenny Riekötter, Kai-Uwe Roelfs, Boje Müller, Jürgen Eirich, Richard M. Twyman, Iris Finkemeier, Dirk Prüfer, Christian Schulze Gronover

**Affiliations:** ^1^ Fraunhofer Institute for Molecular Biology and Applied Ecology IME, Münster, Germany; ^2^ Institute of Plant Biology and Biotechnology, University of Münster, Münster, Germany; ^3^ TRM Ltd, Scarborough, United Kingdom

**Keywords:** SRPPs, small rubber particle proteins, natural rubber, latex, *Taraxacum koksaghyz*, rubber elongation factor family, triterpenoid saponins, stress response

## Abstract

The Russian dandelion (*Taraxacum koksaghyz*) is a promising source of natural rubber (NR). The synthesis of NR takes place on the surface of organelles known as rubber particles, which are found in latex – the cytoplasm of specialized cells known as laticifers. As well as the enzymes directly responsible for NR synthesis, the rubber particles also contain small rubber particle proteins (SRPPs), the most abundant of which are SRPP3, 4 and 5. These three proteins support NR synthesis by maintaining rubber particle stability. We used homology-based searches to identify the whole *TkSRPP* gene family and qPCR to create their spatial expression profiles. Affinity enrichment-mass spectrometry was applied to identify TkSRPP3/4/5 protein interaction partners in *T. koksaghyz* latex and selected interaction partners were analyzed using qPCR, confocal laser scanning microscopy and heterologous expression in yeast. We identified 17 SRPP-like sequences in the *T. koksaghyz* genome, including three apparent pseudogenes, 10 paralogs arranged as an inverted repeat in a cluster with *TkSRPP3/4/5*, and one separate gene (*TkSRPP6*). Their sequence diversity and different expression profiles indicated distinct functions and the latex interactomes obtained for TkSRPP3/4/5 suggested that TkSRPP4 is a promiscuous hub protein that binds many partners from different compartments, whereas TkSRPP3 and 5 have more focused interactomes. Two interactors shared by TkSRPP3/4/5 (TkSRPP6 and TkUGT80B1) were chosen for independent validation and detailed characterization. TkUGT80B1 triterpenoid glycosylating activity provided first evidence for triterpenoid saponin synthesis in *T. koksaghyz* latex. Based on its identified interaction partners, TkSRPP4 appears to play a special role in the endoplasmic reticulum, interacting with lipidmodifying enzymes that may facilitate rubber particle formation. TkSRPP5 appears to be involved in GTPase-dependent signaling and TkSRPP3 may act as part of a kinase signaling cascade, with roles in stress tolerance. TkSRPP interaction with TkUGT80B1 draws a new connection between TkSRPPs and triterpenoid saponin synthesis in *T. koksaghyz* latex. Our data contribute to the functional differentiation between TkSRPP paralogs and demonstrate unexpected interactions that will help to further elucidate the network of proteins linking TkSRPPs, stress responses and NR biosynthesis within the cellular complexity of latex.

## 1 Introduction

The Russian dandelion *Taraxacum koksaghyz* produces large amounts of natural rubber (NR) in its roots and is promising as a new crop for the rubber industry (Salehi et al., 2021). NR is mainly composed of poly(*cis*-1,4-isoprene) produced in the latex, the cytoplasm of specialized cells known as laticifers. Within the latex, NR is stored in organelles known as rubber particles comprising a protein-decorated phospholipid monolayer surrounding a dense NR core (Bae et al., 2020; Collins-Silva et al., 2012; Cornish et al., 1999; Hillebrand et al., 2012; Schmidt et al., 2010b; Wood and Cornish, 2000). Proteins on the rubber particle surface contribute to NR synthesis in *T. koksaghyz* and its close relative *T. brevicorniculatum*, which produces small amounts of NR (Benninghaus et al., 2020; Collins-Silva et al., 2012; Hillebrand et al., 2012; Laibach et al., 2015; Niephaus et al., 2019). These proteins include small rubber particle proteins (SRPPs), the most abundant of which are SRPP3–5 (Collins-Silva et al., 2012; Hillebrand et al., 2012; Schmidt et al., 2010a; Wahler et al., 2012), correlating with the high levels of *SRPP3–5* mRNA in the latex (Benninghaus et al., 2020; Lin et al., 2022; Niephaus et al., 2019). *SRPP* gene silencing in *T. koksaghyz* and *T. brevicorniculatum* caused the depletion of NR and reduced rubber particle stability or NR molecular mass, confirming that SRPPs are needed for efficient NR biosynthesis (Collins-Silva et al., 2012; Hillebrand et al., 2012). Accordingly, several *SRPP* genes are upregulated in the roots of plants that produce large amounts of NR (Panara et al., 2018), and *T. koksaghyz* plants overexpressing the transcription factor MYC2 that induces *SRPP* transcription also accumulate more NR than controls (Wu et al., 2024). Dandelion SRPPs promote NR synthesis by contributing to rubber particle stability and dispersity via steric hindrance, and/or potentially by promoting *cis*-prenyltransferase (CPT) long chain polymerization (Collins-Silva et al., 2012; Hillebrand et al., 2012). The recently published genome assemblies of *T. koksaghyz* revealed 11 *SRPP* paralogs (Lin et al., 2018, 2022) but few studies have considered the entire TkSRPP family (He et al., 2024; Wu et al., 2024). Given that only TkSRPPs 3/4/5 are abundant in rubber particles, these paralogs may have the greatest impact on NR biosynthesis while the others may be involved in stress responses (Laibach et al., 2018).

Despite the clear link between TkSRPPs and NR biosynthesis, detailed information about the functions of individual TkSRPPs is limited and many studies do not refer to specific TkSRPPs and/or use inconsistent nomenclatures (Dong et al., 2023b; He et al., 2024; Mofidi et al., 2024). The functions of TkSRPPs in latex have been proposed based mostly on the characterization of TbSRPPs 1/2/3/4/5. In *Nicotiana benthamiana* cells, TbSRPP1/2/3/4/5 localized to lipid droplets (LDs) and the endoplasmic reticulum (ER), and TbSRPP1 and TbSRPP3 additionally to the cytosol, supporting the hypothesis that rubber particles, like LDs, bud from the ER and that SRPPs might be involved in this process (Cornish et al., 1999; Herman, 2008; Laibach et al., 2018; Wilfling et al., 2014). Furthermore, the expression of *TbSRPP4* and *TbSRPP5* increased LD number and size, respectively (Laibach et al., 2018). A similar effect was observed for LD size in *Arabidopsis thaliana* (Arabidopsis) overexpressing stress-related proteins (SRPs) 1–3, which are homologous to SRPPs (Kim et al., 2016). TbSRPP1–5 form homodimers and heterodimers, and at least TbSRPP3–5 bind to negatively charged lipids, suggesting they interact with rubber particles via pockets of unsaturated phosphatidylcholine (PC) (Laibach et al., 2018). SRPPs may also influence the formation and growth of rubber particles by binding to the minor lipid component phosphatidylinositol (PI) (Bae et al., 2020; Laibach et al., 2018), which causes positive membrane curvature (Harayama and Riezman, 2018).

Dandelion SRPPs belong to the rubber elongation factor (REF) superfamily and share a conserved REF domain, whose function remains unknown, with canonical REF proteins (Dennis and Light, 1989; Laibach et al., 2015; Oh et al., 1999). REF proteins are widespread in plants, even those without latex (Gidda et al., 2013; Horn et al., 2013; Kim et al., 2010, 2004; Seo et al., 2010). In several NR-producing plants other than dandelion, REF family proteins have been associated with NR biosynthesis (Berthelot et al., 2014; Dai et al., 2013; Dennis and Light, 1989; Schmidt et al., 2010b). The *T. brevicorniculatum* major REF protein has a higher molecular mass than TbSRPPs, but is also located on rubber particles. The downregulation of *TbREF* caused NR depletion but did not affect rubber particle stability, therefore suggesting a role in rubber particle biogenesis (Laibach et al., 2015).

In non-rubber plants, like Arabidopsis and avocado (*Persea americana*), REF proteins associate with non-seed LDs (Gidda et al., 2013, 2016; Horn et al., 2013; Kim et al., 2016). LDs have an architecture similar to rubber particles, but store lipids other than NR, such as triacylglycerol (TAG) or sterols (Fernández-Santos et al., 2020; Gidda et al., 2016; Huang, 2018; Kretzschmar et al., 2020; Murphy, 2011; Slocombe et al., 2009). REF proteins from NR-producers and other plants are also involved in stress responses. SRPPs from *T. brevicorniculatum*, sweet potato (*Ipomoea batatas*), pepper (*Capsicum annuum*) and Arabidopsis conferred drought stress tolerance when overexpressed in tobacco (*Nicotiana tabacum*) or Arabidopsis (Kim et al., 2010, 2016; Kim et al., 2012; Laibach et al., 2018; Seo et al., 2010). The genes are induced by drought or other forms of abiotic stress (Guo et al., 2014; Kim et al., 2010, 2016; Kim et al., 2012; Laibach et al., 2018; Seo et al., 2010), and in some cases also by methyl jasmonate (MeJA), abscisic acid (ABA), ethylene, salicylic acid or wounding, via stress and hormone response elements in the promoter (Cao et al., 2017; Dong et al., 2023a; Fricke et al., 2013; He et al., 2024; Wu et al., 2024).

To characterize the function of TkSRPPs in more detail, we identified their interaction partners, providing insight into their roles in NR biosynthesis, stress tolerance, rubber particle composition and biogenesis, and the metabolic and regulatory networks in latex. We therefore determined the spatial expression patterns of all *TkSRPP* genes, followed by affinity enrichment-mass spectrometry (AE-MS) for TkSRPP3/4/5. Two interactors shared by TkSRPP3/4/5 (TkSRPP6 and TkUGT80B1) were chosen for independent validation and detailed characterization.

## 2 Materials and methods

### 2.1 Plant cultivation and tissue processing

We cultivated *T. koksaghyz* and *N. benthamiana* plants under controlled greenhouse conditions (18°C, 16-h photoperiod, 260 PPFD high-pressure sodium lamps with enhanced yellow and red spectrum) as previously described (Unland et al., 2018). *T. koksaghyz* tissues were harvested separately for expression analysis and immediately flash-frozen in liquid nitrogen. After lyophilization, root tissues were pulverized using a ZM 200 Ultra Centrifugal Mill (Retsch, Germany), and leaf tissues were ground under liquid nitrogen with a pestle and mortar. Latex was transferred from cut root surfaces to rubber extraction buffer (REB) [100 mM Tris-HCl pH 7.8, 350 mM sorbitol, 10 mM NaCl, 5 mM MgCl_2_, 5 mM dithiothreitol (DTT)], flash-frozen and used for RNA extraction without further processing.

### 2.2 Heterologous production of TkSRPP3/4/5

SRPPs were expressed in *Escherichia coli* BL21Ai (DE3) cells (Thermo Fisher Scientific, USA) transformed with expression vector pET23a(+) containing codon-optimized sequences of *TkSRPP3/4/5* (**Supplementary Table S1**). Protein expression and purification were carried out as previously described (Laibach et al., 2018).

### 2.3 AE-MS

Latex was harvested from the roots of 12-week-old *T. koksaghyz* plants (line 203-1-ST) as previously described (Post et al., 2012; Niephaus et al., 2019) with slight modifications (**Supplementary Methods S1** in **Supplementary Data Sheet 1**). For affinity enrichment, 100 µL Ni-NTA agarose (Qiagen, Germany) in a 2-mL tube was washed three times with 500 µL Ni-NTA lysis buffer (50 mM NaH_2_PO_4_, 300 mM NaCl, 10 mM imidazole, pH 8.0) and centrifuged (500 g, 5 min, 4°C). We dissolved 200 µg recombinant TkSRPP3/4/5 in 1 mL Ni-NTA lysis buffer containing a protease inhibitor cocktail (diluted 1:10), added this to the Ni-NTA agarose beads, and incubated the mixture for 1 h at room temperature (RT), shaking at 200 rpm. The samples were then centrifuged (500 g, 5 min, RT) and the beads were washed three times with Ni-NTA wash buffer (50 mM NaH_2_PO_4_, 300 mM NaCl, pH 8.0). We diluted 400 µL of the latex or its fractions with 500 µL Ni–NTA wash buffer, added this to the Ni-NTA agarose beads, and repeated the incubation, centrifugation and washing steps as above. After seven further washes with 1 mL Ni-NTA wash buffer, 500 µL of the wash buffer was added to the beads and the samples were analyzed by LC-MS. Beads loaded only with recombinant protein or latex fractions were used as controls. Three replicate samples were prepared.

### 2.4 LC-MS/MS-based quantitative proteomics

LC-MS/MS data acquisition and processing steps were carried out as previously described (Lassowskat et al., 2017). Briefly, proteins were extracted and digested using a modified filter-assisted sample preparation protocol (FASP). After reduction and alkylation, the samples were digested with trypsin, followed by LC-MS/MS analysis using an EASY-nLC 1200 device coupled to a Q Exactive HF mass spectrometer (both from Thermo Fisher Scientific). For details see **Supplementary Methods S2** in **Supplementary Data Sheet 1**.

Raw data were processed using MaxQuant v1.6.9.0 (Cox and Mann, 2008). MS/MS spectra were assigned to the *T. koksaghyz* proteome (Lin et al., 2018). The sequences of 248 common contaminant proteins and decoy sequences were automatically added. Trypsin specificity was required and a maximum of two missed cleavages was allowed. We set cysteine carbamidomethylation as a fixed modification and methionine oxidation, deamidation of N and Q and protein N-terminal acetylation as variable modifications. We applied a false discovery rate of 1% for peptide spectrum matches and proteins, and enabled matching between runs, label-free quantification (LFQ) and iBAQ.

### 2.5 Proteomic data analysis and annotation

Proteins were annotated by BLASTP searching against the NCBI non-redundant and UniProt databases (e ≥ 1 × 10^–3^). Data were processed using Perseus v1.6.0.7 and v2.0.11 (Tyanova et al., 2016). We removed proteins that were only identified by site, reverse hits, or potential contaminants. LFQ intensities were log_2_ transformed and proteins were filtered for those with a mean LFQ intensity in the no-prey control lower than the 10^th^ percentile of all data (19.45 for TkSRPP3 runs; 19.91 for TkSRPP4/5 runs) or undetected in this control, as well as proteins quantified in at least two replicate AE-MS runs. Missing values were imputed using the quantile regression imputation of left-censored missing data (QRILC) algorithm in the package imputeLCMD v2.1 (Lazar and Burger, 2022; R Core Team, 2021) and normal distribution was confirmed by consulting histograms (**Supplementary Figure S1**). Data reproducibility was examined by principal component analysis (PCA). Replicates formed clusters separately from control samples except for rubber phase control 1 from the TkSRPP3 run and pellet phase control 3 from the TkSRPP4/5 run, which were therefore excluded from further analysis (**Supplementary Figure S2**). For enrichment analysis, we applied two sample *t*-tests between AE-MS and no-bait control samples. A permutation-based FDR (q-value) was calculated to correct for multiple testing. To visualize enriched proteins in volcano plots, q-values equal to zero, representing high confidence, were replaced with the next even value smaller than the smallest calculated q-value within the same approach. Volcano plots and Gene Ontology (GO) heat maps were produced using the ggplot2 v3.5.1 R package (Wickham, 2016). Venn diagrams were generated using InteractiVenn (Heberle et al., 2015). GO terms were assigned using eggNOG-mapper (Cantalapiedra et al., 2021). GO enrichment analysis was carried out using the topGO R package v2.50.0 (Alexa and Rahnenfuhrer, 2022). Protein classes were determined using PANTHER 19.0 (Thomas et al., 2022). Protein abundance data (Benninghaus et al., 2020) were processed and heat maps generated in Perseus v2.0.11 (Tyanova et al., 2016).

### 2.6 *In silico* sequence analysis

Protein domains were predicted using Interpro (Paysan-Lafosse et al., 2023). Phosphorylation and *N*-glycosylation sites were predicted using CLC Main Workbench v23.0.3 (Qiagen). Phylogenetic trees were constructed using MEGA11 (Tamura et al., 2021) and *cis*-acting regulatory elements were detected using NSITE-PL (Shahmuradov and Solovyev, 2015). The chromosome map was created using MapChart (Voorrips, 2002). Isoelectric points (*pI*) and protein charges were determined using Prot Pi v. 2.2.29.152 (https://www.protpi.ch/).

### 2.7 Amplification and cloning of *TkSRPP3/4/5* and candidate interactor genes

*TkSRPP* and *TkGUT80B1* coding sequences were amplified from *T. koksaghyz* latex cDNA with flanking primers based on the genomic sequences (Lin et al., 2018) (**Supplementary Table S2**). Amplified fragments were digested with restriction enzymes indicated in the primer names and ligated into the Gateway pENTR 4 entry vector (Thermo Fisher Scientific).

For the split-ubiquitin membrane yeast two-hybrid (SUY2H) assays, plasmids pRS313 and pRS314 (Sikorski and Hieter, 1989) were modified to form Gateway destination vectors. The Gateway cassette was amplified from pAG304-P_GAL1_-*ccdB* (Addgene, USA) using primers M13 rev and attR *Bgl*II fw, and overhangs were prepared by digestion with *Bgl*II and *Kpn*I. The pRS314 interim vector was digested with *Bam*HI and *Kpn*I and ligated with the Gateway cassette. For pRS313, the Gateway cassette was amplified from pBatTL (Jach et al., 2006) using primers attR *Spe*I fw and attR *Age*I rev, and ligated into the digested pRS313 vector. *TkSRPPs, TkUGT80B1* and *mEmerald* were introduced into pRS313-*ccdB-CRU* and pRS314-*Nua-ccdB* using Gateway LR Clonase II mix (Thermo Fisher Scientific). For co-immunoprecipitation (co–IP), *TkSRPP3/4/5* and *TkUGT80B1* were transferred to pAG425-P_GPD_-*ccdB-Cerulean* and pAG423-P_GPD_-*ccdB*-HA (Addgene), respectively, by Gateway cloning. For *N. benthamiana* transient expression experiments, genes were introduced into the Gateway-compatible vector pBatTL-*ccdB-Cerulean* as previously described (Epping et al., 2015; Unland et al., 2018). To test TkUGT80B1 activity in yeast, the *TkUGT80B1* coding sequence was inserted into pAG423-P_GAL1_-*ccdB* (Addgene) by Gateway cloning. All constructs were validated by Sanger sequencing.

### 2.8 RNA extraction, cDNA synthesis and quantitative PCR

RNA extraction, cDNA synthesis and qPCR were carried out as previously described (Niephaus et al., 2019) with slight modifications (**Supplementary Methods S3** in **Supplementary Data Sheet 1**). Briefly, normalized expression was calculated using the ΔC_q_ method (Equation 1) relative to the mean C_q_ value of the reference genes. To account for different primer efficiencies, mean Cq values of technical replicates were adjusted by multiplication with an adjustment coefficient based on primer efficiency (Equation 2).


(1) $$normalized\;expression=2^{mean\;adjC_{q}\left(reference\;genes\right)-adjC_{q}\left(target\;gene\right)}$$

(2) $$adjC_{q}=C_{q}\times \left(\frac{-1}{slope}\times \log_{2}10\right)$$

### 2.9 Subcellular localization studies

Transient expression in *N. benthamiana* leaves was carried out using pBatTL constructs in which the protein of interest was fused to the N-terminus of the blue fluorescent protein Cerulean (Müller et al., 2010). Monomeric red fluorescent protein (mRFP) C-terminally fused to the N-terminal sequence of CYP51G1 (CYP51G1-mRFP) was used to mark the cytosolic surface of the ER (Bassard et al., 2012). Two-pore-channel 1 (TPC1) was fused to orange fluorescent protein (OFP) as a tonoplast marker (Batistič et al., 2010). To test LD localization, we co-expressed Arabidopsis *LEAFY COTYLEDON 2* (*AtLEC2*) to induce LD formation, and infiltrated the leaves with Nile red solution (Santos Mendoza et al., 2005). *AtLEC2* was amplified from *A. thaliana* cDNA that was obtained as previously described (Jekat et al., 2013) using flanking primers introducing restriction sites for cloning into pENTR4. This construct was used for Gateway cloning into pBatTL-*ccdB* using Gateway LR Clonase II mix (Thermo Fisher Scientific) (Müller et al., 2010). Leaf discs were analyzed by confocal laser scanning microscopy (CLSM) using a Stellaris 8 microscope (Leica Microsystems, Germany). Cerulean fluorescence was detected at 445–550 nm (excitation at 440 nm), mRFP fluorescence at 570–648 nm (excitation at 555 nm), and Nile red fluorescence at 571–587 nm (excitation at 541 nm).

### 2.10 SUY2H assay

The *Saccharomyces cerevisiae* strain InvSc1 (Thermo Fisher Scientific) was transformed with combinations of pRS313 and pRS314 using the lithium-acetate method (Agatep et al., 1998). Positive clones were identified by colony PCR using gene-specific and vector primers. They were grown for 5–6 h at 30°C in 1 mL synthetic defined (SD) medium containing 50 µM CuSO_4_ and lacking histidine, tryptophan and methionine. Cultures were centrifuged (19,000 *g*, 1 min, RT) and the OD_600_ was adjusted to 1 using 1× TE. We transferred 10 µL of three serial dilutions to SD medium lacking histidine and tryptophan, or to selective media containing (1) 300 µM methionine, 50 µM CuSO_4_ and lacking histidine, tryptophan and uracil, or (2) 300 µM methionine, 50 µM CuSO_4_ and 1 g/L 5-fluoroorotic acid (5-FOA), and lacking histidine and tryptophan. The plates were incubated at 30°C for 2–3 days.

### 2.11 Co-immunoprecipitation

Yeast strain InvSc1 was transformed with each of the pAG425-P_GPD_-*TkSRPP*-Cerulean constructs and pAG423-P_GPD_-*TkUGT80B1*-HA alone or with the three combinations of *TkUGT80B1* and each *TkSRPP*. Positive transformants were identified by colony PCR using gene-specific and vector primers. To test for the interactions between TkUGT80B1, TkSRPP3 and TkSRPP5, 5 mL of SD medium lacking histidine, leucine or both were inoculated with a single colony of each genotype, incubated at 30°C until the OD_600_ reached 3 and harvested by centrifugation (4,000 *g*, 10 min, RT). Cells expressing *TkSRPP4* and a control expressing only *TkUGT80B1* were cultivated at 20°C to enable sufficient recombinant protein synthesis. Overnight cultures were used to inoculate 50 mL SD medium to an OD_600_ of 0.3. The main cultures were then cultivated at 20°C, shaking at 140 rpm. They were harvested by centrifugation after 42 h (4,000 *g*, 10 min, RT) and washed once with 10 mL 1× TE. Proteins were extracted with 1 mL cell lysis buffer comprising 20 mM Tris-HCl pH 7.5, 150 mM NaCl, 1 mM Na_2_EDTA, 1 mM EGTA, 1% Triton X-100, 2.5 mM Na_4_P_2_O_7_, 1 mM β–glycerophosphate, 1 mM Na_3_VO_4_ and 1 µg/ml leupeptin (Cell Signaling Technology, USA) supplemented with 1 mM phenylmethylsulfonylfluoride (PMSF) and cOmplete EDTA-free protease inhibitor cocktail (Merck, Germany) for 1–5 min at 30 Hz in an MM400 bead mill (Retsch) followed by centrifugation (11,000 *g*, 5 min, 4°C). ChromoTek GFP-Trap magnetic agarose (Proteintech Group, USA) was used for immunoprecipitation according to the manufacturer’s instructions. Extracts were incubated with the beads for 1 h at 4°C on a platform rocker. Proteins were eluted in 50 µL 5× SDS loading buffer containing 100 mM DTT. Samples were separated by SDS-PAGE on 10% SDS polyacrylamide gels and analyzed as previously described (Niephaus et al., 2019) with modifications. Briefly, membranes were incubated with either an anti-GFP primary antibody (Clontech Laboratories, USA; #632380) diluted 1:2,000, or with an anti-HA primary antibody (Merck; #H3663) diluted 1:1,000–2,000, followed by washing and incubation with either a goat anti-mouse IgG coupled to alkaline phosphate (Merck; #A3562) diluted 1:10,000, followed by detection using SIGMAFAST BCIP/NBT tablets (Merck), or a goat anti-mouse IgG coupled to horseradish peroxidase (Thermo Fisher Scientific; #32430) diluted 1:1,500, followed by detection using SuperSignal West Dura Extended Duration Substrate (Thermo Fisher Scientific).

### 2.12 TkUGT80B1 activity in yeast

We transformed a *S. cerevisiae* strain, previously engineered for enhanced triterpenoid production and expressing *T. koksaghyz lupeol synthase* (*TkLup*) under the control of a galactose-inducible promoter (Bröker et al., 2018), with pAG423-P_GAL1_-*TkUGT80B1* or the empty vector using the lithium-acetate method (Agatep et al., 1998). As a control, the same strain lacking *TkLUP* was transformed with *TkUGT80B1* or the empty vector. Positive transformants were identified by colony PCR using gene-specific and vector primers (or two vector primers for the empty vector). For yeast cultivation, we inoculated 5 mL SD medium lacking histidine and tryptophan with a single colony of each genotype and incubated it overnight at 30°C on a rolling wheel. We inoculated 50 mL of the same medium, supplemented with 150 µM CuSO_4_ to repress sterol synthesis, with the overnight cultures to an OD_600_ of 0.2 and incubated them at 30°C, shaking at 140 rpm. When the cultures reached an OD_600_ of 0.5–0.6, inducible gene expression was activated by switching to SD medium containing 2% galactose instead of glucose. Cells were harvested when cultures reached an OD_600_ of 4. Metabolites were extracted from lyophilized yeast pellets by adding glass beads and 1 mL ethyl acetate, followed by lysis for 30 min at 30 Hz in an MM400 bead mill. After centrifugation (11,000 *g*, 1 min), the supernatant was transferred to a fresh tube and the extraction was repeated twice with 0.5 mL ethyl acetate, each time vortexing for 15 min. The extracts were analyzed by LC-MS/MS using an UltiMate 3000 Rapid Separation System (Thermo Fisher Scientific) and amazon speed ion trap MS (Bruker Corporation, USA) or using an Acquity Premier LC system (Waters Corporation, UK) coupled to a Synapt XS 4k (Waters Corporation) ion mobility time-of-flight mass spectrometer. Extracts were separated using a Reprosil Pur Basic C18 (5 µm particle size, 4×250 mm) (Analytik Altmann, Germany) by isocratic elution with 10% mobile phase A (90:10 *v/v* isopropanol:water + 10 mM ammonium formate) and 90% mobile phase B (methanol + 10 mM ammonium formate). The flow rate was 0.4 mL/min and the run time 45 min. The column temperature was set to 23°C. For the identification of lupeol, an authentic standard was analyzed separately.

## 3 Results

### 3.1 Analysis of the *TkSRPP* gene family in *T. koksaghyz*

**Figure 1:**
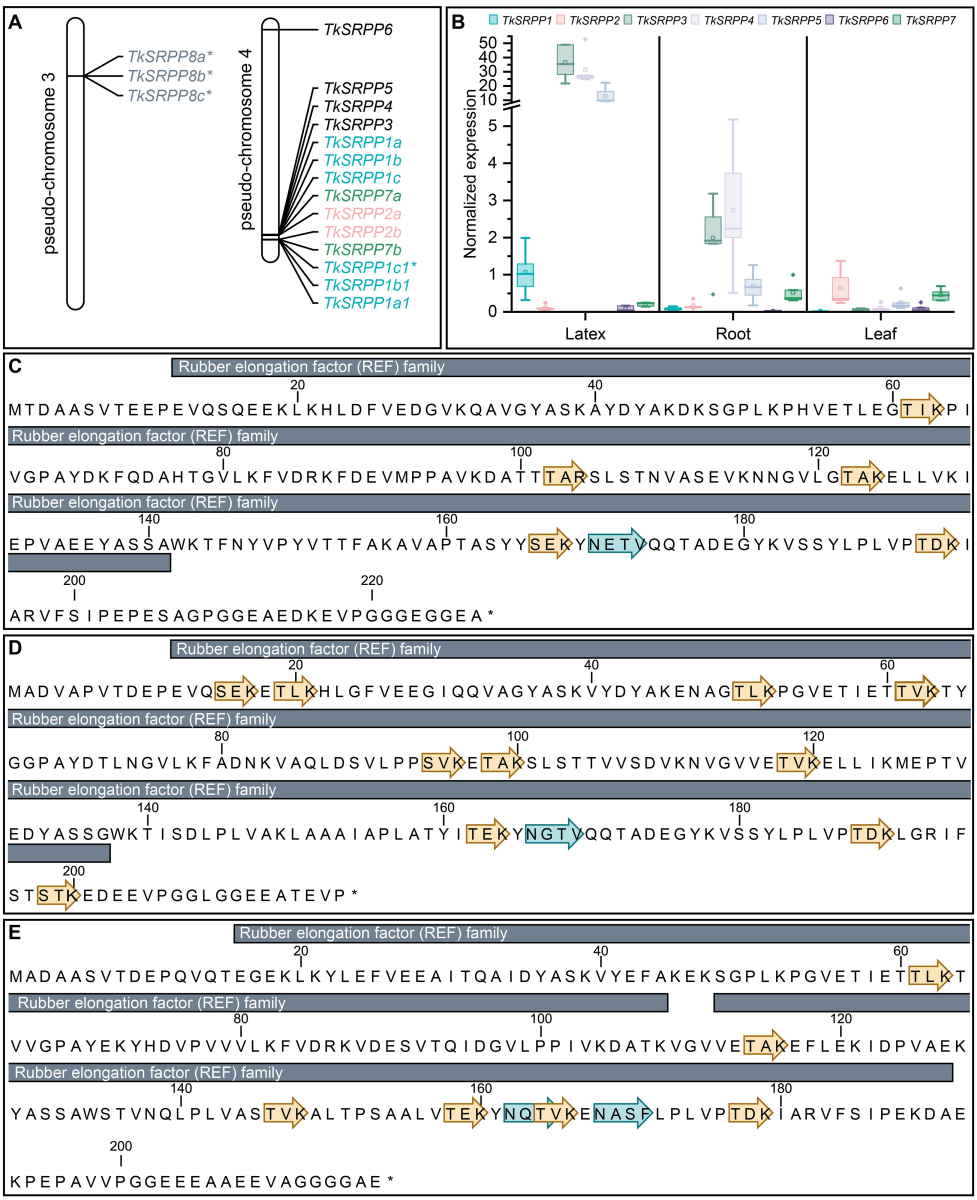
The *TkSRPP* gene family. **(A)** Pseudo-chromosome maps showing the loci for the *TkSRPP* family. Asterisks indicate *TkSRPPs* with premature stop codons. **(B)** *TkSRPPs* have different spatial gene expression patterns. Normalized gene expression levels in different tissues of 10-week-old wild-type *T. koksaghyz* plants. Box plots represent data from four or five individual plants. Expression levels of *TkSRPP1*, *2* and *7* represent transcripts of all corresponding gene copies. Expression levels were normalized against *elongation factor-1 α* (*TkEF1α*) and *ribosomal protein L27* (*TkRP*). **(C-E)** Protein sequences of TkSRPP3 **(C)**, TkSRPP4 **(D)** and TkSRPP5 **(E)** showing predicted protein domains (InterPro), phosphorylation sites (yellow arrows) and *N*-glycosylation sites (blue arrows*). Asterisks mark the end of the amino acid sequences.

We identified 17 *SRPP*-like sequences in the *T. koksaghyz* genome (Lin et al., 2018, 2022). Twelve of them cluster on pseudo-chromosome 4 (**Figure 1A**). The nomenclature we applied is based on sequence similarity to known orthologs (mainly from *T. brevicorniculatum*) and the first publicly available annotations (Collins-Silva et al., 2012; Hillebrand et al., 2012; Laibach et al., 2018; Schmidt et al., 2010a). Corresponding sequence IDs and comparisons with other published nomenclatures are summarized in **Supplementary Table S3**. We identified one copy each of *TkSRPP3/4/5* and *TkSRPP6*, the latter being the only full *TkSRPP* gene located outside the cluster, on the other arm of pseudo-chromosome 4. The most recent genome annotation (Lin et al., 2022) lists *TkSRPP5* as a pseudogene, but the earlier version (Lin et al., 2018) includes an open reading frame, which we confirmed by amplification from cDNA. Similarly, the 2022 genome assembly contains a premature stop codon for TkSRPP6, but amplification of an open reading frame from cDNA confirmed its integrity. We found that *TkSRPP3/4/5* form a contiguous set along with 10 additional paralogs. This indicates an inverted duplication in which the adjacent copies of the first five genes run in the opposite direction. Because the first three contiguous genes are more closely related to each other than other paralogs, we named them *TkSRPP1a-c* and their copies with sequence identities of 93–100% *TkSRPP1a1/b1/c1* respectively (**Table 1**). However, *TkSRPP1c1* contains a stop codon after 30 bp and has only 93% identity to *TkSRPP1c*, suggesting that functional redundancy has allowed sequence divergence in this case. The next two genes of the cluster were designated *TkSRPP7a* and *TkSRPP2a*, and their copies *TkSRPP7b* and *TkSRPP2b*. *TkSRPP2a* is not annotated in the 2022 genome release, but was identified manually by homology searches. Three additional *TkSRPP* sequences sharing 99.9% identity form another cluster on pseudo-chromosome 3 and were designated *TkSRPP8a-c*.These sequences appeared to originate from incomplete gene transposition and duplication of TkSRPP2a/b, because they contain only the first 654 bp (exon I, intron I, part of exon II) of TkSRPP2a/b which could encode a 50 aa peptide. The high conservation between *TkSRPP8a/b/c* suggests a relatively recent duplication event. Because of this truncation resulting in an incomplete REF domain, we excluded *TkSRPP8* from further analysis. *TkSRPP* gene duplications have been proposed before (He et al., 2024), but our classification differs in that we identified five additional genes and established a nomenclature based on previous publications. The TkSRPPs, sequence identities and predicted protein properties are summarized in **Table 1**.

**Table 1:** Comparison of TkSRPP sequences and protein properties.

DNA aa	TkSRPP1a	TkSRPP1a1	TkSRPP1b	TkSRPP1b1	TkSRPP1c	TkSRPP1c1*	TkSRPP2a	TkSRPP2b	TkSRPP3	TkSRPP4	TkSRPP5	TkSRPP6	TkSRPP7a	TkSRPP7b	TkSRPP8a*	TkSRPP8b*	TkSRPP8c*
TkSRPP1a	100	100	94.7	97.4	96.4	90.1	74	74.4	63.5	56.9	62.5	52.4	73.5	73.5	40.7	40.7	40.7
TkSRPP1a1	100	100	94.7	97.4	96.4	90.1	74	74.4	63.5	56.9	62.5	52.4	73.5	73.5	40.7	40.7	40.7
TkSRPP1b	96.1	96.1	100	96.1	95.1	91	73.5	74	62.6	55.8	62.1	52.4	73.6	73.6	40.7	40.7	40.7
TkSRPP1b1	97.4	97.4	97.8	100	97	90.4	73.5	74	63.7	57.2	62.5	52.9	73.6	73.8	40.9	40.9	40.9
TkSRPP1c	96.6	96.6	95.7	97	100	93.1	74	74.3	62.8	56.5	62.5	54.3	73.5	73.6	40.3	40.3	40.3
TkSRPP1c1*	x	x	x	x	x	100	70	69.8	59.4	54	60	51.3	69.3	69.3	37.7	37.7	37.7
TkSRPP2a	67.3	67.3	67.8	67.3	67.8	x	100	99.4	64.7	60.3	66	55.6	88.2	87.1	45	45	45
TkSRPP2b	67.8	67.8	68.3	67.8	68.3	x	99.5	100	65.2	60.6	66.5	55.2	88.3	87.2	45.2	45.2	45.2
TkSRPP3	50.7	50.7	49.8	51.2	49.3	x	51	51.5	100	71.8	72.7	47.9	66.0	65.7	40.6	40.6	40.6
TkSRPP4	44.6	44.6	43.8	44.6	43.1	x	45	45	64.5	100	68.5	46.3	61.1	61.3	40.2	40.2	40.2
TkSRPP5	51	51	51.4	51.9	52.4	x	55.6	56.2	61.3	59.9	100	52.6	65.6	65.6	41.2	41.2	41.2
TkSRPP6	45.8	45.8	45.6	45.3	44.9	x	48.6	48.6	38.4	37.2	40.2	100	55.4	55	39.9	39.9	39.9
TkSRPP7a	70.2	70.2	70.7	70.2	69.7	x	88.1	87.6	54	48	56.7	51	100	98.6	44.1	44.1	44.1
TkSRPP7b	69.7	69.7	70.2	69.7	69.2	x	86.7	86.2	54.5	48	56.7	51	98.6	100	44.5	44.5	44.5
TkSRPP8a*	66	66	64	64	62	x	98	96	49	46.9	51	44	74	74	100	99.9	99.9
TkSRPP8b*	66	66	64	64	62	x	98	96	49	46.9	51	44	74	74	100	100	99.9
TkSRPP8c*	66	66	64	64	62	x	98	96	49	46.9	51	44	74	74	100	100	100
*pI*	5.4	5.4	5.4	5.5	5.8	x	8.3	8.3	4.8	4.5	4.6	5.7	8.6	8.3	4.8	4.8	4.8
Charge pH 7.4	-9.3	-9.3	-10,4	-9.3	-9.1	x	1.3	1.3	-13.7	-14.8	-15.8	-6.9	2.3	1.3	-3.7	-3.7	-3.7

Percent identities are shown for TkSRPP DNA and protein sequences. The isoelectric point (*pI*) and protein charge at pH 7.4 are predicted for all paralogs. Sequence identities were determined using Clustal Omega, whereas *pI* and charge were predicted using Prot pi. Asterisks indicate either paralogs containing a premature stop codon (*TkSRPP1c1*) or partial genes (*TkSRPP8a/b/c*) (see text for details).

We designed primer pairs to quantify *TkSRPP* gene expression in the tissues of wild-type *T. koksaghyz* plants. No discriminating primers could be designed for the proposed gene duplications, so the data for *TkSRPP1*, *TkSRPP2* and *TkSRPP7* reflect the expression of all duplicates. We observed extremely strong *TkSRPP3/4/5* expression in latex (**Figure 1B**), in agreement with published RNA-Seq data and previously reported high protein levels (Collins-Silva et al., 2012; Lin et al., 2018, 2022; Niephaus et al., 2019). *TkSRPP3* and *TkSRPP4* showed similarly high transcript levels, each about twice the level of *TkSRPP5*. A comparable profile was observed in the roots, which contain considerable amounts of latex (**Figure 1B**). In leaves, *TkSRPP3/4/5* expression was low. *TkSRPP1* was also predominantly expressed in latex, albeit at level of one tenth or less of that of *TkSRPP3/4/5*. *TkSRPP2* expression notably differed from the others, with the highest level in leaves. *TkSRPP6* was expressed at low levels and *TkSRPP7* at moderate levels in all tissues. These diverging sequences and spatial expression profiles indicate specialized, tissue-specific functions. Because latex is of special interest in *T. koksaghyz*, the remarkably strong expression of *TkSRPP3/4/5* and their contribution to NR biosynthesis prompted us to screen for protein interaction partners of these paralogs in latex (Collins-Silva et al., 2012). We also identified several *N*-glycosylation and phosphorylation sites that could play a role in these interactions and their biological functions (**Figures 1C–E**).

### 3.2 Identification of TkSRPP3/4/5 protein interaction partners by AE-MS

**Figure 2:**
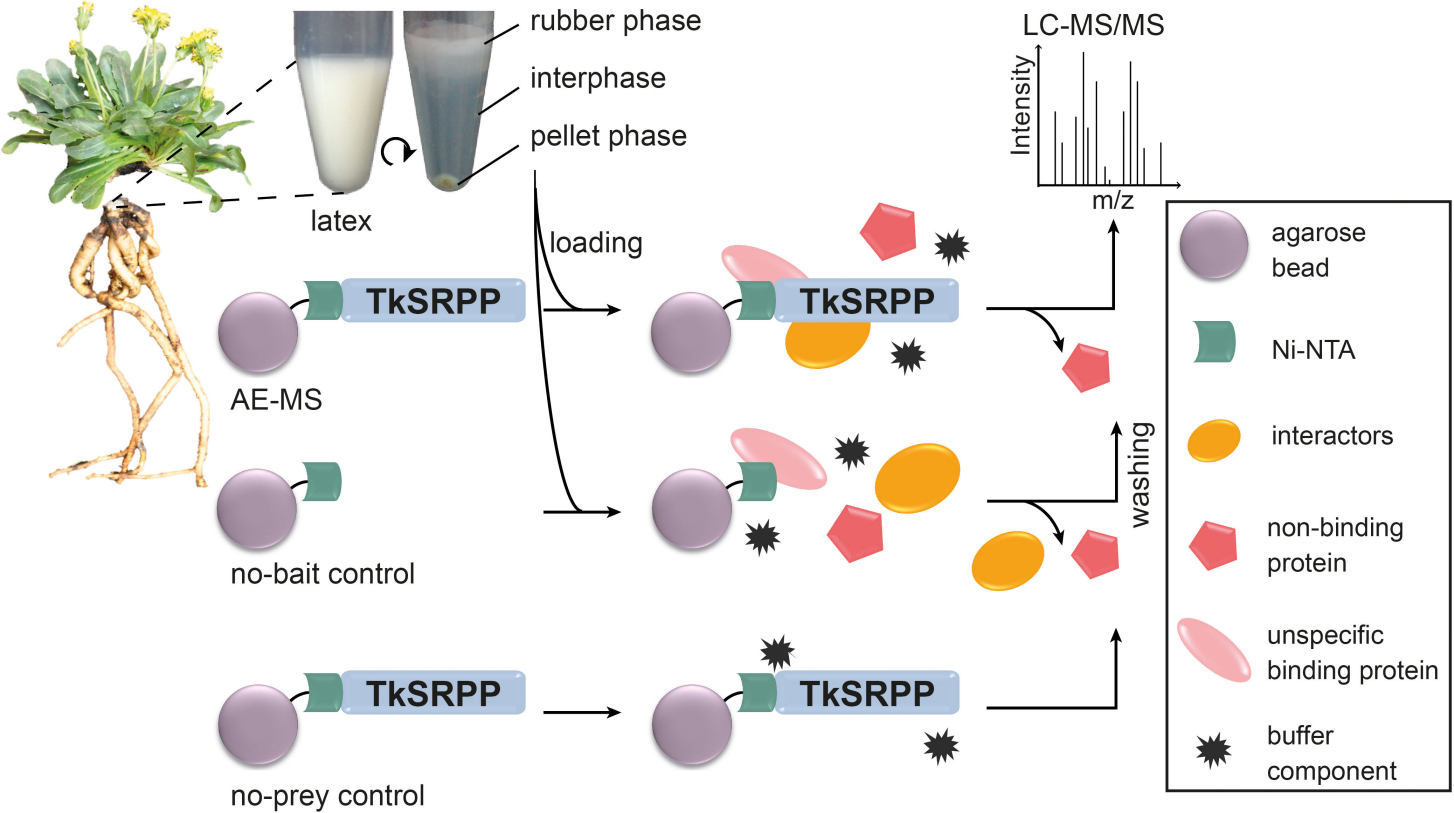
Overview of AE-MS experimental design. Latex was harvested from the roots of wild-type *T. koksaghyz* plants and separated by centrifugation. Recombinant TkSRPP3/4/5 expressed in *E. coli* were bound to Ni-NTA agarose beads and loaded with the four separate latex fractions. Fresh agarose beads were loaded with the separate latex fractions as no-bait controls. Agarose beads bound to TkSRPP3/4/5 without exposure to latex fractions served as background (no-sample) controls. All samples were washed and analyzed by LC-MS/MS for the identification of enriched proteins.

Recombinant TkSRPPs with His_6_ tags were produced in *E. coli* and coupled to agarose beads before mixing with whole latex as well as the separate rubber phase (RP), interphase (IP) and pellet phase (PP) obtained by centrifugation (**Figure 2**). The latex phases are thought to comprise different cellular fractions, with the RP mostly containing rubber particles (Collins-Silva et al., 2012; Schmidt et al., 2010b), the PP containing most of the membrane and organelle components of the latex, and the IP containing most of the cytosol. This was supported by the enrichment of GO terms of the cellular component (CC) category in one fraction relative to all detected latex proteins (**Supplementary Table S4**), enabling us to infer interaction sites in the laticifers and potentially detect interactions with less-abundant proteins that are enriched in particular phases. This is interesting because of potential TkSRPP functions other than rubber particle stabilization and TbSRPP3/4/5 were localized in the ER and cytosol in *N. benthamiana* (Laibach et al., 2018). We used two controls to ensure qualitatively reliable results. First, we used TkSRPP-His_6_-coupled beads that were not loaded with latex as no-prey controls for each paralog. Second, uncoupled agarose beads were loaded with each latex fraction as no-bait controls. Proteins were identified by LC-MS/MS and quantified by LFQ using the MaxQuant software suite. The no-bait controls were used to calculate protein enrichments (**Figure 2**).

**Figure 3:**
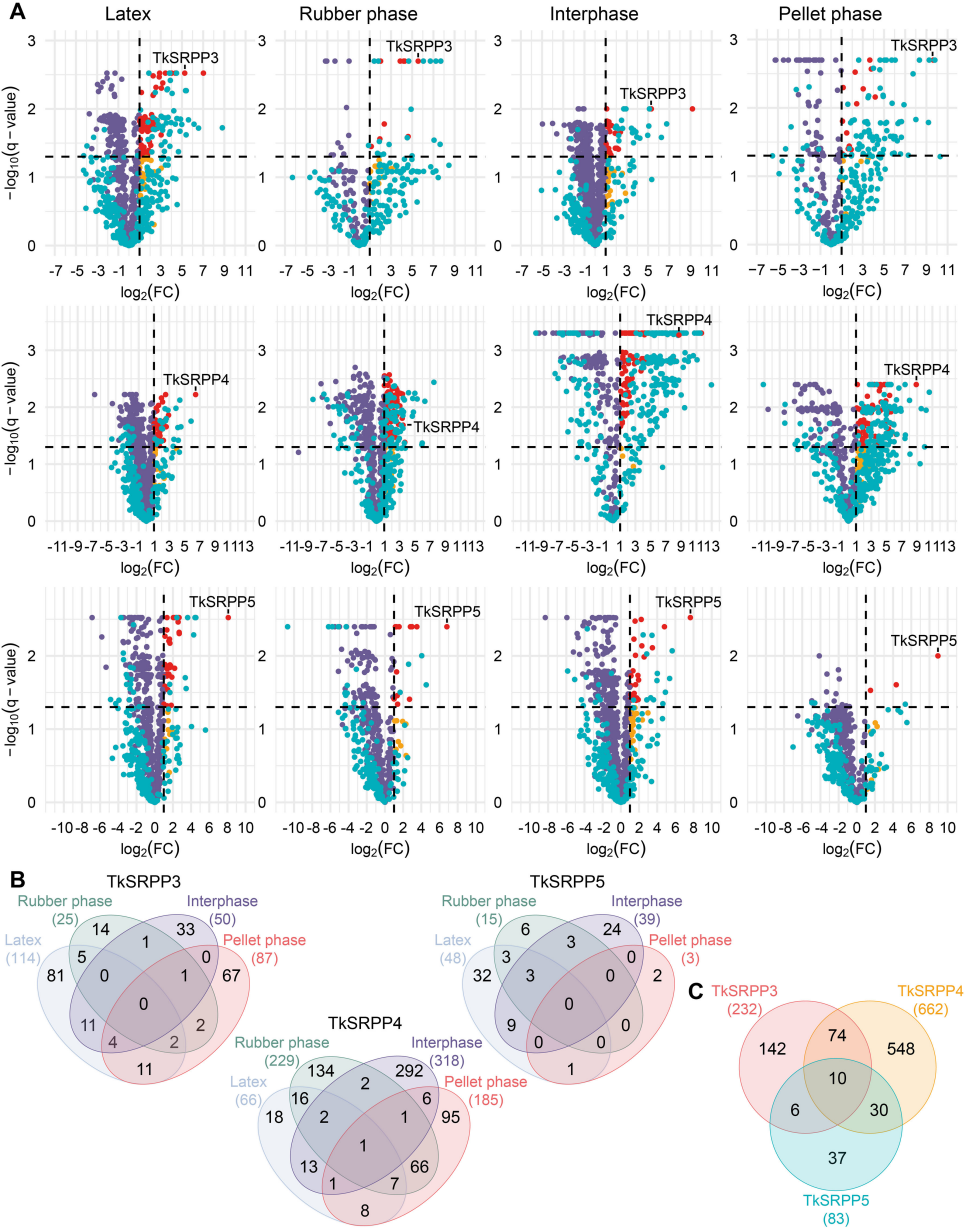
AE-MS reveals the enrichment of overlapping sets of proteins with each TkSRPP from each latex fraction. **(A)** Volcano plots showing the enrichment of proteins from four different latex fractions – latex, rubber phase (RP), interphase (IP), pellet phase (PP) – by TkSRPP3/4/5 compared to no-bait controls. The log_2_FC values are plotted against the –log_10_(q-value). Dashed lines show threshold values for proteins considered as interactors (log_2_FC ≥ 1; –log_10_(q-value) > 1.3. Red dots highlight proteins in this area. Orange dots represent enriched proteins with higher q-values. Violet dots represent proteins that are not enriched due to their log_2_FC. Blue dots mark proteins that were not detected in all replicates of the AE-MS and the control so that missing LFQ values were generated by imputation. These include both, proteins considered as interactors and those outside the thresholds. The latter may be of interest if they are close to the threshold values because imputation can affect enrichment factors and significance levels by sample variance. **(B)** Venn diagrams showing the number of total proteins enriched from each latex fraction with each TkSRPP and the overlaps between fractions. **(C)** The Venn diagram shows the total numbers of different proteins significantly enriched with one TkSRPP from all latex fractions.

Proteins were considered as potential interaction partners when they were significantly enriched compared to the no-bait control (LFQ log_2_ fold change (FC) ≥ 1; q < 0.05) (**Figure 3A**). Given the nature of the experiment, the MS data contained a relatively large number of missing values that were mainly assumed to be missing not at random (MNAR). Accordingly, missing values were imputed using QRLIC (**Supplementary Figure S1** in **Supplementary Data Sheet 1**), which performs best for left-censored MNAR data (Wei et al., 2018). Imputation is necessary to enable quantitative statistical analysis, but it can influence data analysis and interpretation. Therefore, we did not strictly exclude proteins from the group of potential interaction partners if they fell outside the defined thresholds for log_2_FC and q-value but were subjected to imputation (**Figure 3A**, blue dots). Our candidate lists thus contain proteins meeting the defined threshold criteria and represent potential interaction partners with the highest confidence according to our data analysis strategy, but we do not limit TkSRPP interaction partners to these proteins (**Supplementary Tables S7–S9** in **Supplementary Data Sheet 1**).

### 3.3 Characterization of the interactome datasets

For each TkSRPP, four independent datasets were obtained representing whole latex and its three fractions, revealing different numbers of interaction partners in partially overlapping sets (**Figures 3B, C**). The volcano plots show that the respective bait TkSRPP was one of the most strongly enriched proteins in all AE-MS experiments (**Figure 3A**). The number of interaction partners identified for TkSRPP5 (83) was much lower than for TkSRPP3 (232) and TkSRPP4 (662), this could have been caused by different affinities of TkSRPP3/4/5 to the Ni-NTA agarose beads or the protein stability on the Ni-NTA agarose. Further, it must be distinguished between the total number of hits and the number of different proteins detected within each latex phase. Some of the interactors (16% for TkSRPP3, 19% for TkSRPP4 and 23% for TkSRPP5) were detected under more than one condition, providing more confidence in their veracity (**Figure 3B**). The large number of interacting proteins enriched exclusively in one phase confirmed that the use of separate latex phases allowed the identification of more specialized interactions and low-abundance interactors. For TkSRPP3, most interacting proteins were enriched from the whole latex (114), followed by the PP (87), IP (50) and RP (25). In contrast, most TkSRPP4 interactors were enriched from the IP (318), followed by the RP (229), PP (185) and whole latex (66, 18 of which were exclusive to whole latex). We found 75 proteins enriched from both the RP and PP, suggesting they are not exclusive to rubber particles but are also found in other organelles. For TkSRPP5, only three interactors were enriched from the PP, with one also enriched from whole latex. Most TkSRPP5 interactors were enriched from whole latex (48) and the IP (39), with 12 enriched from both. The interactomes indicated that TkSRPP4 is a promiscuous hub protein that binds many partners from different compartments, whereas TkSRPP5 interacts more specifically, primarily with proteins present in rubber particles or the cytosol. We also found 10 interactors common to TkSRPP3, 4 and 5 (**Figure 3C**). TkSRPP4 shared the most interactors with the other paralogs, probably reflecting the presence of more interactors overall. More than half of the proteins interacting with TkSRPP5 also interacted with TkSRPP3 or TkSRPP4, leaving only 37 unique to TkSRPP5. In contrast, most TkSRPP3 and TkSRPP4 interactors were exclusive.

Because RP interactors are of particular interest for the elucidation of the role of TkSRPP3/4/5 in NR biosynthesis, compared the TkSRPP3/4/5 RP interactomes and found that four interactors are shared by TkSRPP4 and TkSRPP5 (**Supplementary Table S6**) whereas all TkSRPP3 RP interactors are exclusive, again highlighting the functional specialization on the rubber particle, as previously shown on LDs in *N. benthamiana* for TbSRPP4 and TbSRPP5 but not TbSRPP3 (Laibach et al., 2018). The shared TkSRPP4/TkSRPP5 RP interactors comprised Ras-related protein Rab11C, a CBL-interacting protein kinase, α-ketoglutarate-dependent dioxygenase, and a protein similar to an uncharacterized protein from lettuce. Notably, TkSRPP5 was identified as an interaction partner of TkSRPP3 by co-enrichment from whole latex (log_2_FC 1.1) and the IP (log_2_FC 2.7), but TkSRPP3 was not identified as an interactor when TkSRPP5 was the bait. For the closely related homologs TbSRPP3/4/5, all pairwise interactions have been shown by bimolecular fluorescence complementation (BiFC) (Laibach et al., 2018).

**Figure 4:**
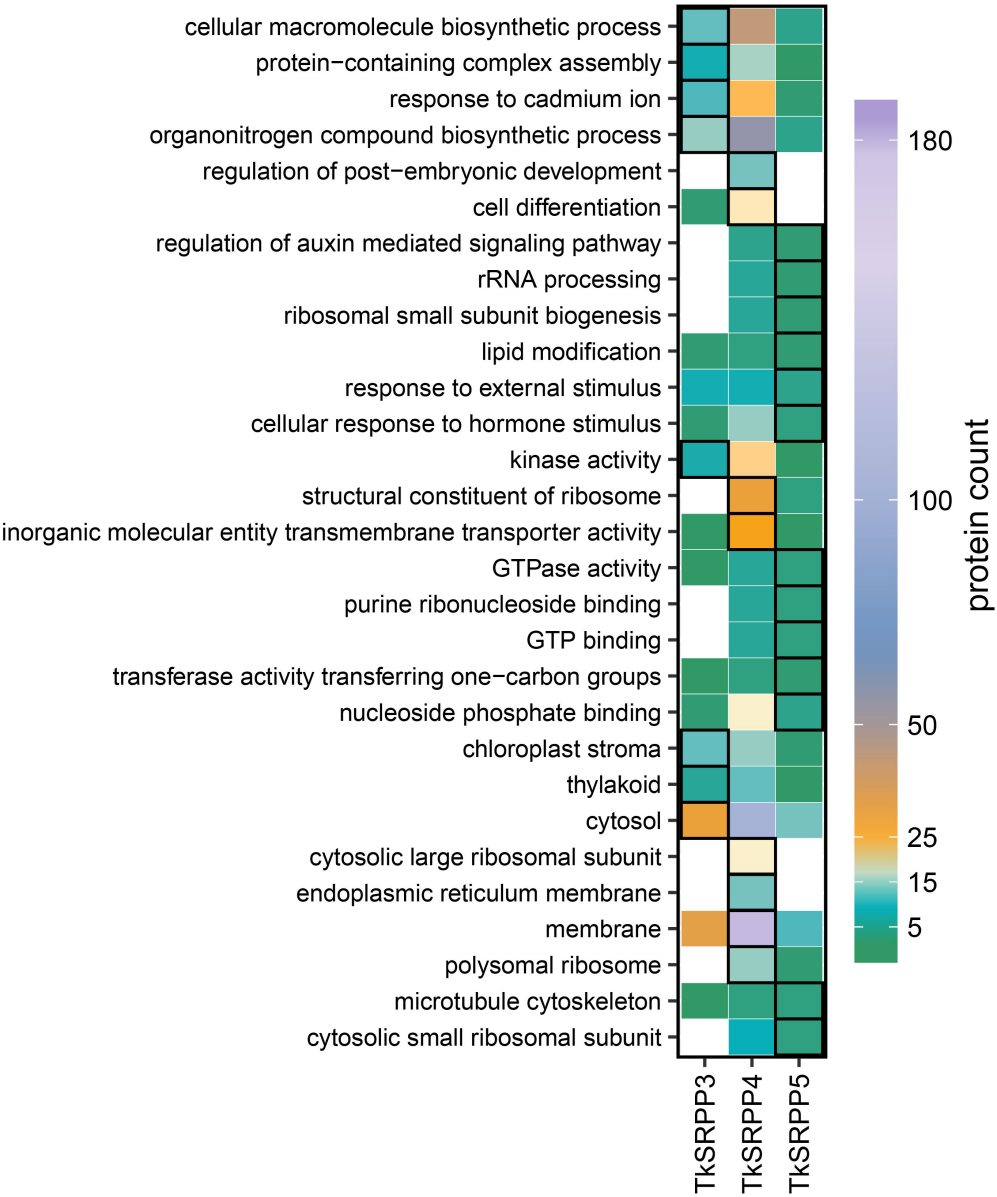
Exclusive TkSRPP3/4/5 interactors differ in their assigned GO terms. Heat map showing the number of interactors exclusive to one TkSRPP paralog assigned to a GO term. Framed boxes highlight GO terms significantly enriched (p-value < 0.05) among the exclusive interactors of one TkSRPP paralog compared to all TkSRPP3/4/5 interactors. Significance levels were calculated using a weighted Fisher’s exact test.

To gain insight into the specific functions of each TkSRPP in latex, we screened for GO terms enriched within each subset of paralog-specific interactors (**Figure 4**). Interestingly, ‘chloroplast stroma’ (12), ‘thylakoid’ (5) and ‘cytosol’ (30) associated proteins were significantly enriched in the CC category among the exclusive TkSRPP3 interactors (compared to all TkSRPP interactors) whereas the TkSRPP4 interactors were significantly enriched for ‘polysomal ribosome’ (15) and ‘cytosolic large ribosomal subunit’ (19), ‘ER membrane’ (13) and ‘membrane’ (181). Accordingly, in the molecular function (MF) category, ‘structural constituent of ribosome’ (30) was enriched along with ‘inorganic molecular entity transmembrane transporter activity’ (26). For the TkSRPP5 interactome, the enrichment of ‘cytosolic small ribosomal subunit’ (3) in the CC category aligns with the enrichment of ‘ribosomal small subunit biogenesis’ (2) and ‘rRNA processing’ (2) in the biological process (BP) category. GO analysis thus indicated that TkSRPP4 and TkSRPP5 are associated with cytosolic ribosomal processes. It is possible that the enrichment of ribosomal proteins was favored because of the assumed connection between TkSRPPs and the ER, the likely origin of rubber particles. But their detection may also reflect the high affinity of ribosomal proteins for the agarose beads (Keilhauer et al., 2015). For TkSRPP5 interactors, we further observed the enrichment of ‘microtubule cytoskeleton’ (3) in the CC category, as well as proteins related to responses to hormone and external stimuli (3, 4), ‘lipid modification’ (2) and ‘regulation of auxin mediated signaling pathway’ (2) in the BP category. For the TkSRPP3 interactors, the BP category terms ‘cellular macromolecule biosynthetic process’ (12), ‘protein-containing complex assembly’ (8), ‘response to cadmium ion’ (11) and ‘organonitrogen compound biosynthetic process’ (15) were enriched. The TkSRPP4 interactors were enriched for two more general BP terms relating to ‘regulation of post-embryonic development’ (13) and ‘cell differentiation’ (20). In the MF category, ‘kinase activity’ (7) was the only enriched term among the TkSRPP3 interactors, whereas the TkSRPP5 interactors were enriched for terms related to GTP, purine ribonucleoside and nucleoside phosphate binding (3, 3, 4), ‘GTPase activity’ (3) and ‘transferase activity’ (2).

Protein class analysis based on annotated UniProt IDs revealed similar distributions for all three interactomes, partially matching the enriched GO terms (**Figure 4**; **Supplementary Figure S3**). Prominent protein classes among the interactors included metabolite interconversion enzymes such as oxidoreductases, transferases and hydrolases, protein-modifying enzymes such as proteases, and protein-binding affinity modulators such as protease inhibitors and G-proteins.

We extracted data relating to the abundance of each interactor from our dataset in wild-type *T. koksaghyz* roots at 8, 12 and 24 weeks (Benninghaus et al., 2020), and clustered the interactors and corresponding bait TkSRPP according to their temporal accumulation profiles (**Supplementary Figure S4**). This identified interacting proteins with similar abundance profiles as their TkSRPP partners, which supports their status as interaction partners because gene co-expression is more likely for interacting proteins than random protein pairs (Ge et al., 2001; Jansen et al., 2002; von Mering et al., 2002). TkSRPPs 3/4/5 were all assigned to clusters with increasing protein levels over time. All three are already highly abundant after 8 weeks of growth, so that interactions with proteins from the same cluster may represent basal interactions rather than conditional interactions in response to particular stimuli.

To identify protein interactions related to NR synthesis, the interactors were correlated with proteins that are enriched or depleted when *TkCPT-like 1* (*TkCPTL1*) is downregulated in the latex of *T. koksaghyz* plants by RNA interference (RNAi) (data obtained by Niephaus et al., 2019) (**Table 2**). These *TkCPTL1*-RNAi plants produce significantly less NR than wild-type plants because TkCPTL1 is thought to form heterodimers with TkCPT1 and/or TkCPT2 and thus assemble into a *cis*PT complex that catalyzes the synthesis of poly(*cis*-1,4-isoprene) on the surface of rubber particles (Niephaus et al., 2019). We identified TkCPT1 as a TkSRPP3 interactor and TkCPT1 was significantly less abundant in the *TkCPTL1*-RNAi plants. An interaction between TkSRPP3 and TkCPT1 highlights the connection between TkSRPP3 and NR synthesis on the rubber particle surface, and is consistent with the reported interaction between *Hevea brasiliensis* SRPP and CPT6 (Brown et al., 2017). TkSRPP4 interacted with 10 proteins whose abundance changed in the NR-depleted transgenic plants, including the rate-limiting enzyme of the mevalonate (MVA) pathway: 3–hydroxy-3-methylglutaryl-CoA reductase (HMGR). The MVA pathway provides the C_5_ building block isopentenyl diphosphate (IPP) for NR synthesis, and was downregulated in the RNAi lines. Other interactors involved in isoprenoid metabolism, including squalene epoxidase 1 (SQE1) and germacrene oxidase (GAO), were more abundant in the *TkCPTL1*-RNAi lines. Another downregulated interactor was a homolog of a ricin B-like lectin, and additional lectin homologs were found in the TkSRPP3 and TkSRPP4 interactomes. One TkSRPP5 interactor similar to a lettuce (*Lactuca sativa*) putative methyltransferase was also downregulated in the RNAi plants.

**Table 2:** Interaction partners of TkSRPP3/4/5 differentially accumulated in the latex of *TkCPTL1-RNAi* plants compared to wild-type controls.

	ID	NCBI (non-redundant) Protein names (identity)	UniProtKB/Swiss-Prot Protein names (identity)	Log_2_FC				
				*TkCPTL1-*RNAi - WT	Latex	RP	IP	PP
TkSRPP3	evm.model.utg11341.6	cis-prenyltransferase CPT2 [*Taraxacum brevicorniculatum*] (98.05%)	Dehydrodolichyl diphosphate synthase 6 (Dedol-PP synthase 6) (EC 2.5.1.-) (52.80%)	-3.54	2.28		1.25	
TkSRPP4	evm.model.utg10104.22	3-hydroxy-3-methylglutaryl-CoA reductase 2, partial [*Taraxacum kok-saghyz*] (98.20%)	3-hydroxy-3-methylglutaryl coenzyme A reductase 2-A (HMG-CoA reductase 2) (Hydroxymethylglutaryl-CoA reductase) (PgHMGR2) (EC 1.1.1.34) (74.46%)	-1.96			2.68	
	evm.model.utg7969.4	hypothetical protein LSAT_6X38201 [*Lactuca sativa*] (83.07%)	Ricin B-like lectin R40G3 (Osr40g3) (57.52%)	-1.77			1.73	
	evm.model.utg2280.9	probable isoprenylcysteine alpha-carbonyl methylesterase ICMEL2 [*Lactuca sativa*] (77.08%)	Probable isoprenylcysteine alpha-carbonyl methylesterase ICMEL2 (EC 3.1.1.n2) (Isoprenylcysteine methylesterase-like protein 2) (61.10%)	-1.46			1.10	2.01
	evm.model.utg3903.5	CRAL-TRIO domain-containing protein YKL091C-like [*Lactuca sativa*] (81.48%)	Sec14 cytosolic factor (Phosphatidylinositol/phosphatidyl-choline transfer protein) (PI/PC TP) (Sporulation-specific protein 20) (31.12%)	-1.23			1.80	
	evm.model.utg29345.1	SEC14 cytosolic factor-like [*Lactuca sativa*] (87.05%)	CRAL-TRIO domain-containing protein YKL091C (26.29%)	-1.68			5.12	
	evm.model.utg29792.19	myo-inositol oxygenase 4 *[Artemisia annua*] (89.84%)	Inositol oxygenase 4 (EC 1.13.99.1) (Myo-inositol oxygenase 4) (AtMIOX4) (MI oxygenase 4) (74.13%)	-1.83				3.01
	evm.model.utg17642.9	probable glycerol-3-phosphate acyltransferase 8 isoform X2 *[Lactuca sativa*] (80.28%)	Glycerol-3-phosphate 2-O-acyltransferase 4 (AtGPAT4) (EC 2.3.1.198) (Glycerol-3-phosphate acyltransferase 4) (61.22%)	-5.65				3.86
	evm.model.utg24682.2	germacrene A oxidase [*Lactuca sativa*] (95.49%)	Germacrene A hydroxylase (EC 1.14.14.95) (Germacrene A oxidase) (LsGAO) (95.29%)	1.16			4.05	
	evm.model.utg16440.3	squalene epoxidase 1 [*Taraxacum kok-saghyz*] (98.12%)	Squalene monooxygenase SE1 (EC 1.14.14.17) (Squalene epoxidase 1) (PgSQE1) (SE) (SE1) (gse) (76.24%)	3.11			1.43	
	evm.model.utg8052.7	plastidial pyruvate kinase 2 isoform X2 [*Lactuca sativa*] (93.21%)	Plastidial pyruvate kinase 2 (PKp2) (EC 2.7.1.40) (Plastidial pyruvate kinase 1) (PKP1) (Pyruvate kinase III) (Pyruvate kinase isozyme B1, chloroplastic) (PKP-BETA1) (Plastidic pyruvate kinase beta subunit 1) (95.43%)	1.12			5.63	
TkSRPP5	evm.model.utg1886.1	putative methyltransferase DDB_G0268948 [*Lactuca sativa*] (91.83%)	Putative methyltransferase DDB_G0268948 (EC 2.1.1.-) (32.14%)	-1.30				5.34

The mean log_2_FC between transgenic and wild-type plants reported in an earlier study (Niephaus et al., 2019) are shown with the log_2_FC of AE-MS experiments and the corresponding latex fractions as determined in the current study.

### 3.4 Confirmation of selected TkSRPP3/4/5 interactors identified by AE-MS

We selected two of the 10 candidate interactors shared by TkSRPP3/4/5 for confirmation using a second method and characterized them in more detail. The first candidate (annotated as TkSRPP6) was selected because *TkSRPP6* is the only complete *TkSRPP* gene found outside the main cluster, and its function has not been studied thus far. The second candidate, annotated as a sterol 3–β-glucosyltransferase/UDP-glycosyltransferase (UGT) 80B1 family member, was designated TkUGT80B1. It was selected because glycosides are known to be involved in plant defense and stress responses, but the role of UGTs in latex has not been investigated. The proteins were enriched to different levels in different phases in the AE-MS datasets for TkSRPP3, 4 and 5 (**Table 3**).

**Table 3:** Enrichment of TkSRPP6 and TkUGT80B1 by TkSRPP3/4/5 based on AE-MS data.

Genome ID	Given name	Log_2_FC											
		TkSRPP3				TkSRPP4				TkSRPP5			
		L	RP	IP	PP	L	RP	IP	PP	L	RP	IP	PP
evm.model.utg2059.21	TkSRPP6		4.7	5.0		1.1						2.2	
evm.model.utg4564.7	TkUGT80B1				2.5			6.9				3.5	

Log_2_FC values are provided for each bait TkSRPP and latex fraction in which TkSRPP6 and TkUGT80B1 were significantly enriched. L, latex; RP, rubber phase; IP, interphase; PP, pellet phase.

**Figure 5:**
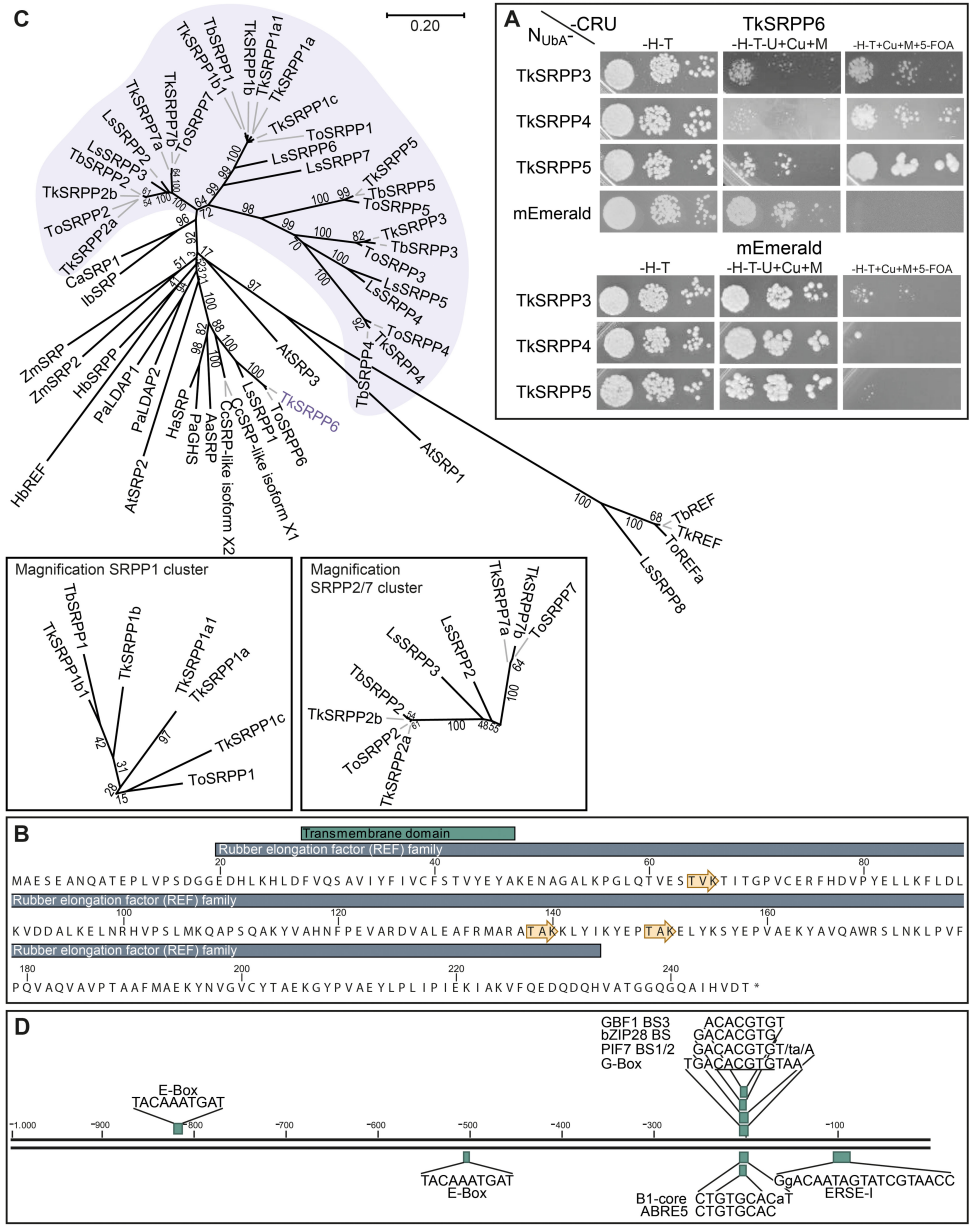
General characterization of TkSRPP6. **(A)** Split-ubiquitin yeast-two hybrid (SUY2H) indicating protein interactions between TkSRPP6 and TkSRPP4/TkSRPP5. Yeast expressing TkSRPP6 C-terminally fused to the C-terminal part of ubiquitin and URA3 as a reporter (CRU), and TkSRPP3/4/5 N-terminally fused to the N-terminal part of ubiquitin (N_UbA_), were dropped in three different dilutions on selective media and grown for 2–3 days. Medium lacking histidine and tryptophan (–H–T) was used as a control medium to select for the plasmid encoding the proteins of interest. Medium additionally lacking uracil but containing 50 µM CuSO_4_ and 300 µM methionine (–H–T–U+Cu+M) was used to select for URA3 activity. Medium containing uracil and 1 g/L 5-FOA (–H–T+Cu+M+5-FOA) was used to select for URA3 inactivity reflecting bait/prey interactions. The monomeric mEmerald fluorophore was used as a negative control. **(B)** TkSRPP6 protein sequence containing a REF domain and a short N-terminal transmembrane domain predicted by InterPro. Yellow arrows represent predicted phosphorylation sites. **(C)** Phylogenetic analysis reveals clustering of TkSRPP6 with stress-related REF family proteins from non-rubber plants, separated from the other TkSRPPs. Multiple sequences were aligned using CLUSTALW and the phylogenetic tree was constructed using the neighbor-joining algorithm and a bootstrap of 500. Values at branches indicate bootstrap values. The phylogenetic distance is indicated by the scale bar. Accession numbers: AaSRP, *Artemisia annua* stress-related protein (PWA88416.1); AtSRP1, *Arabidopsis thaliana* REF/SRPP-like protein At1g67360 (NP_176904.1); AtSRP2, *A. thaliana* REF/SRPP-like protein At2g47780 (NP_182299.1); AtSRP3, *A. thaliana* REF/SRPP-like protein At3g05500 (NP_187201.1); CaSRP1, ADI60300.1; CcSRPP-like isoform X1, *Cynara cardunculus* var. *scolymus* stress-related protein-like isoform X1 (XP_024981582.1); CcSRPP-like isoform X2, *C. cardunculus* var. *scolymus* stress-related protein-like isoform X2 (XP_024981583.1); HaSRP, *Helianthus annuus* putative stress-related protein (A0A251TGA8); HbREF, *Hevea brasiliensis* REF (P15252); HbSRPP, *H. brasiliensis* SRPP (O82803); IbSRP, *Ipomoea batatas* stress-related protein (ABP35522.1); LsSRPP1, *Lactuca sativa* SRPP1 (XP_023771881.1); LsSRPP2, (AJC97799.1); LsSRPP3, (AJC97800.1); LsSRPP4, (AJC97801.1); LsSPP5, (AJC97802.1); LsSRPP6, (AJC97803.1); LsSPP7, (AJC97804.1); LsSRPP8, (AJC97805.1); PaGHS, *Parthenium argentatum* rubber synthesis protein (AAQ11374.1); PaLDAP1, *Persea americana* lipid droplet-associated protein 1 (AGQ04593.1); PaLDAP2, *P. americana* lipid droplet-associated protein 2 (AGQ04594.1); TbREF, *Taraxacum brevicorniculatum* REF (A0A291LM03); TbSRPP1, *T. brevicorniculatum* SRPP1 (M9PNN1); TbSRPP2, (AGE89407.1); TbSRPP3, (M9PNQ7); TbSRPP4, (M9PNN3); TbSRPP5, (M9PNM8); TkREF, *Taraxacum koksaghyz* REF (GWHPBCHF036022); TkSRPP1a, GWHPAAAA010568; TkSRPP1a1, GWHPBCHF033216; TkSRPP1b, GWHPAAAA043688; TkSRPP1b1, GWHPBCHF033215; TkSRPP1c, GWHPAAAA010568; TkSRPP2: deduced from identified gene locus (**Supplementary Table S2**); TkSRPP2a, GWHPAAAA010566; TkSRPP3, GWHPAAAA015362; TkSRPP4, GWHPAAAA015361; TkSRPP5, GWHPAAAA015359; TkSRPP6, GWHPAAAA016929; TkSRPP7, GWHPBCHF033106; TkSRPP7a, GWHPBCHF033213; ZmSRP, *Zea mays* stress-related protein (ACG39345.1); ZmSRP2, *Z. mays* REF/SRPP-like protein (NP_001149834.1). TkSRPP sequence IDs originate from published genome data (Lin et al., 2018, 2022). **(D)** 1 kb promotor region of *TkSRPP6* containing different *cis*-acting regulatory elements connected to plant stress responses. Promotor region was extracted from the published *T. koksaghyz* genome (Lin et al., 2022) and regulatory elements were determined using NSITE-PL.

**Figure 6:**
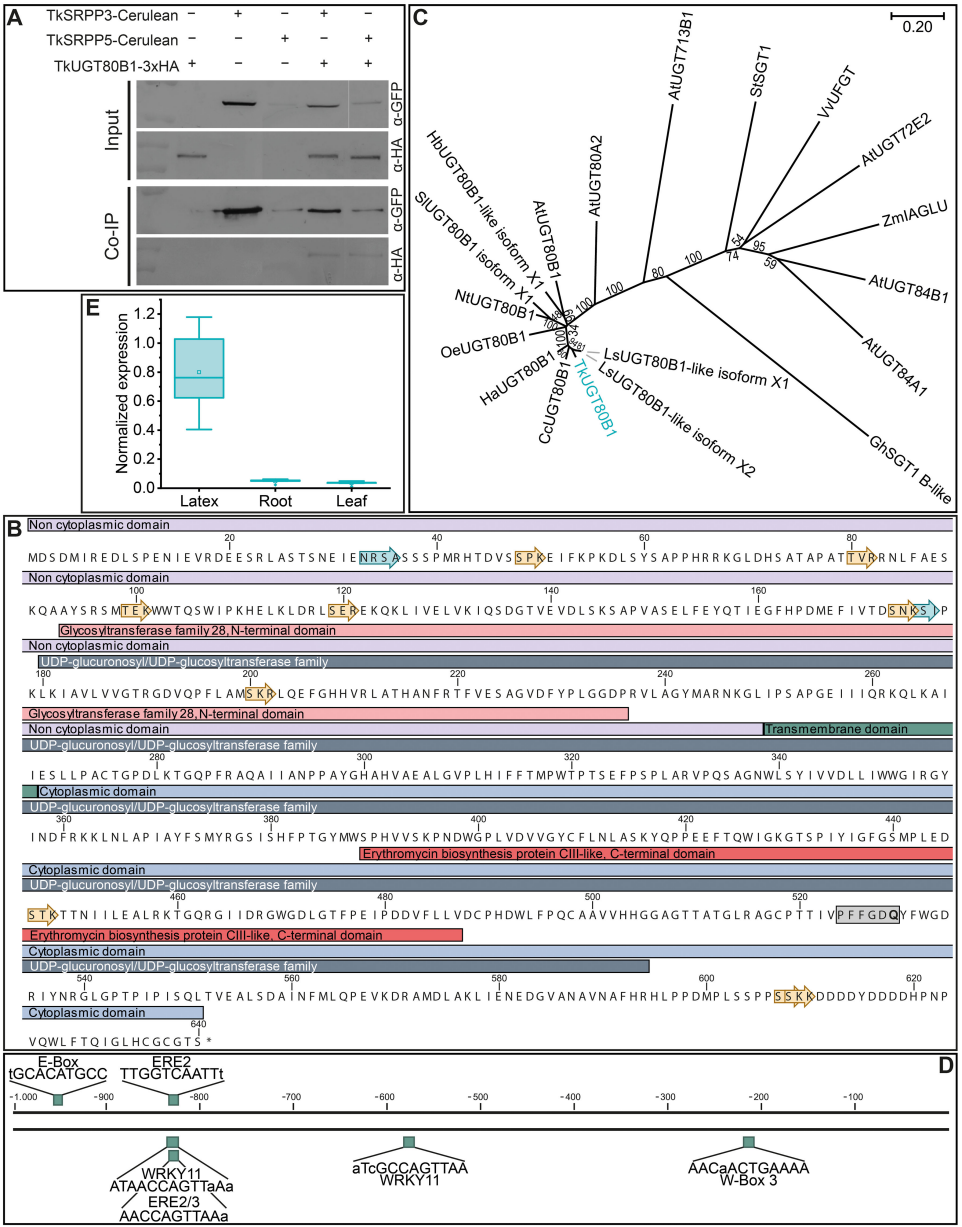
General characterization of TkUGT80B1. **(A)** Co-Immunoprecipitation (Co-IP) assay showing the interaction of TkUGT80B1-3xHA with TkSRPP3/5-Cerulean. The top two panels show the input samples and the bottom two panels protein detection after immunoprecipitation with an α-GFP antibody. Fusion proteins were extracted from yeast cells. **(B)** TkUGT80B1 protein sequence with assigned domains. The gray box highlights the UDPGT motif with glutamine in the last position, characteristic of UDP-glucosyltransferases. Yellow arrows represent phosphorylation and cyan arrows *N*-glycosylation sites predicted using CLC Main Workbench. **(C)** Phylogenetic analysis reveals clustering of TkUGT80B1 with UGT80B1 proteins from other Asteraceae. Multiple sequences were aligned using CLUSTALW and the phylogenetic tree was constructed using the neighbor-joining algorithm and a bootstrap of 500. Values at branches indicate bootstrap values. The phylogenetic distance is indicated by the scale bar. Accession numbers: AtUGT72E2, *Arabidopsis thaliana* UDP-glycosyltransferase superfamily protein UGT72E2 (NP_201470.1); AtUGT80A2, *A. thaliana* sterol 3-β -glucosyltransferase UGT80A2 (NP_566297); AtUGT80B1, *A. thaliana* sterol 3-β -glucosyltransferase UGT80B1 (NP_175027); AtUGT84A1, *A. thaliana* UDP-glycosyltransferase 84A1 (NP_193283.2); AtUGT713B1, *A. thaliana* glycosyltransferase UGT713B1 (NP_568452); CcUGT80B1, *Cynara cardunculus* var. *scolymus* sterol 3-β-glucosyltransferase UGT80B1 (XP_024976598.1); GhSGT1 B-like, *Gossypium hirsutum* sterol glucosyltransferase 1 homolog B-like (JN004107); HaUGT80B1, *Helianthus annuus* sterol 3-β-glucosyltransferase UGT80B1 (XP_035834958.1); HbUGT80B1-like isoform X1, *H. brasiliensis* sterol 3-β-glucosyltransferase UGT80B1-like isoform X1 (XP_021673215.1); LsUGT80B1-like isoform X1, *Lactuca sativa* sterol 3-β-glucosyltransferase UGT80B1-like isoform X1 (XP_023742443.1); LsUGT80B1-like isoform X2, *L. sativa* sterol 3-β-glucosyltransferase UGT80B1-like isoform X2 (XP_023742444.1); NtUGT80B1, *Nicotiana tomentosiformis* sterol 3-β-glucosyltransferase UGT80B1 (XP_009595972.1); OeUGT80B1, *Olea europaea* subsp. *europaea* sterol 3-β-glucosyltransferase UGT80B1 (CAA2989377.1); SlUGT80B1 isoform X1*, Solanum lycopersicum* sterol 3-β-glucosyltransferase UGT80B1 isoform X1 (XP_004237799.1); StSGT1, *Solanum tuberosum* UDP-galactose:solanidine galactosyltransferase (AB48444.2); TkUGT80B1, *Taraxacum koksaghyz* UDP-glycosyltransferase 80B1 (GWHPAAAA034502); VvUFGT, *Vitis vinifera*, UDP glucose:flavonoid 3-*O*-glucosyltransferase (AAB81683.1); ZmIAGLU, *Zea mays* indole-3-acetate β-glucosyltransferase (Q41819). *T. officinale* sequences were obtained from unpublished data. **(D)** *TkUGT80B1* 1-kb promoter region containing different *cis*-acting regulatory elements associated with plant stress responses. Promoter region was extracted from the published *T. koksaghyz* genome (Lin et al., 2022) and regulatory elements were determined using NSITE-PL. **(E)** *TkUGT80B1* is predominantly expressed in latex. Normalized gene expression levels in different tissues of 10-week-old wild-type *T. koksaghyz* plants. Box plots represent data from five individual plants. Expression levels were normalized against *elongation factor-1 α* (*TkEF1α*) and *ribosomal protein L27* (*TkRP*).

For SUY2H, TkSRPP3/4/5 baits were N-terminally fused to a modified N-terminal ubiquitin fragment (N_UbA_) with lower affinity for the ubiquitin C-terminus (C_Ub_), thus minimizing false positive results (Johnsson and Varshavsky, 1994). The N_UbA_ fusions were co-expressed with TkSRPP6 C-terminally fused to C_Ub_. Interactions reconstitute functional ubiquitin, leading to the cleavage and degradation of the URA3 reporter, thus conferring uracil auxotrophy and resistance to 5-FOA (Johnsson and Varshavsky, 1994; Reichel and Johnsson, 2005). We used the monomeric fluorescent protein mEmerald combined with the TkSRPPs as negative controls. Using this system, we were able to confirm that TkSRPP6 interacts with TkSRPP4 and TkSRPP5. For TkSRPP3 the growth pattern was indistinct but suggested weak interaction (**Figure 5A**). This supports the initial screens, but indicates that protein interactions are dependent on the experimental conditions and highlights the importance of independent confirmation. For interactions between TkSRPP3/4/5 and TkUGT80B1, we were able to pull down TkUGT80B1-3×HA with Cerulean-tagged TkSRPP3 and TkSRPP5 by Co-IP, but we could not confirm the interaction with TkSRPP4 in this experimental setup (**Figures 6A**; **Supplementary Figure S5**). In summary, additional methods confirmed four of six pairwise interactions indicated by AE-MS, two for each candidate, suggesting the unconfirmed interactions are restricted to specific native conditions or part of bigger complexes.

### 3.5 Sequence analysis of TkSRPP6 and TkUGT80B1

TkSRPP6 *in silico* analysis identified the REF domain common to all known dandelion SRPPs and REF proteins, as well as three potential phosphorylation sites (**Figure 5B**). Phylogenetic comparisons showed that TkSRPP6 has diverged from other TkSRPPs and is more closely related to other REF proteins (including those involved in stress responses in plants that do not produce NR) than to the tightly clustered TkSRPP1/2/3/4/5 and 7 (**Figure 5C**). This was supported by protein identities of ~60% between TkSRPPs 3/4/5, but only 37–49% when TkSRPP6 was compared to the other paralogs (**Table 1**). We therefore screened a 1-kb region of the *TkSRPP6* promoter for stress-responsive elements (**Figure 5D**; **Supplementary Table S5**), revealing E–box elements (CANNTG) at positions –200 and –500 bp relative to the start codon, and the core sequence of a G-box type E-box (CACGTG) and extended G-box elements at –800 bp (**Figure 5D**) (Galvāo et al., 2019; Nagao et al., 1993; Shahmuradov and Solovyev, 2015). The G-box recruits G-box binding factors (GBFs), which include bZIP and bHLH proteins such as MYC2 (Heim et al., 2003; Menkens et al., 1995; Sibéril et al., 2001; Williams et al., 1992; Zhang et al., 2019). G-box elements mediate the effects of hormones, light and temperature (Eyal et al., 1993; Guiltinan et al., 1990; Hong et al., 1995; Mason et al., 1993; Shaikhali et al., 2012; Toledo-Ortiz et al., 2014), whereas E-box elements regulate temperature-dependent and circadian expression in stress-responsive genes (Liu et al., 2015; Seitz et al., 2010). These findings suggest that *TkSRPP6* is transcriptionally regulated by different stress factors, in agreement with other data for *SRPP* genes (Cao et al., 2017; Dong et al., 2023a; Fricke et al., 2013; He et al., 2024; Hillebrand et al., 2012).

TkUGT80B1 was found to contain a UDP-glucuronosyltransferase/UDP-glucosyltransferase domain, an N-terminal domain similar to glycosyltransferase family 28, and a C-terminal domain resembling that of CIII-like, another glycosyltransferase, including the nucleotide diphosphate sugar binding site (**Figure 6B**) (Moncrieffe et al., 2012). The last amino acid in the so–called UDPGT motif differs between UDP-glucosyltransferases (where it is glutamine) and UDP-galactosyltransferases (histidine), so the presence of glutamine in TkUGT80B1 suggests it has UDP-glucose transferase activity (**Figure 6B**, gray box) (Kubo et al., 2004). Sequence analysis also predicted that the C-terminal domain is cytosolic, separated from the N-terminal part by a transmembrane domain of 19 amino acids. The protein contains nine putative phosphorylation sites and two *N*-glycosylation sites. Phylogenetic analysis supported the relationship between TkUGT80B1 and UGT80B1 enzymes from the family Asteraceae and other plants, as well as more distant relationships with other UGT families (**Figure 6C**). The *TkUGT80B1* promoter (**Figure 6D**) contains three elicitor response elements (ERE1-3), which contribute to fungal elicitor-mediated gene expression (Yang et al., 1998). Additionally, we found an E-box, two WRKY11-binding sites (one overlapping with ERE2/3) and one WRKY40-binding site (W-box). WRKY transcription factors are involved in plant defense (Javed and Gao, 2023), suggesting stress-responsive transcriptional regulation, which ties in with the role of glycosylated secondary metabolites in the plant defense system (Hussain et al., 2019; Louveau and Osbourn, 2019).

### 3.6 Gene expression profiles of TkSRPP3/4/5 and their interaction partners TkSRPP6 and TkUGT80B1

We had already determined the spatial expression profile of *TkSRPP6* when comparing SRPP paralogs (**Figure 1**). Applying the same approach to *TkUGT80B1* in 10-week-old wild-type *T. koksaghyz* plants, we observed strong expression in the latex (consistent with the AE-MS experiments) but low expression in roots and leaves (**Figure 6E**), similar to the expression profiles of *TkSRPP3/4/5*. The previous detection of TkUGT80B1 protein in roots may reflect the large amount of latex in this tissue (Benninghaus et al., 2020).

**Figure 7:**
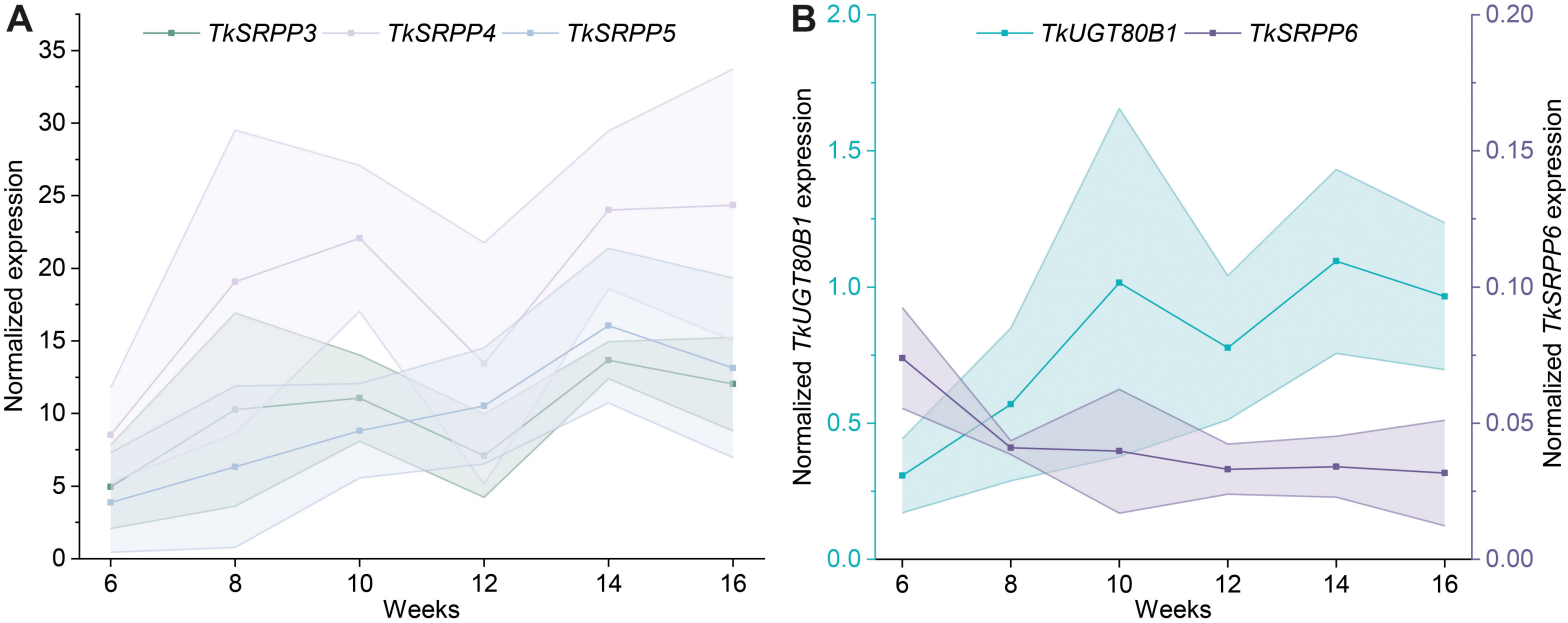
*TkSRPP4-5* and *TkUGT80B1* show similar temporal expression patterns in latex. Normalized gene expression levels of **(A)** *TkSRPP3/4/5* and **(B)** *TkUGT80B1* and *TkSRPP6* in *T. koksaghyz* wild-type latex over time. Data points are means of 4–7 individual plants. The shaded areas represent the areas within in the standard deviations. Expression levels were normalized against *elongation factor-1α* (*TkEF1α*) and *ribosomal protein L27* (*TkRP*).

Temporal expression profiling in latex revealed a steady increase in *TkSRPP3/4/5* mRNA levels during weeks 6–14 (**Figure 7**), as shown for root protein levels before (Benninghaus et al., 2020). However, transcript levels stayed constant or decreased slightly between weeks 14 and 16 (**Figure 7A**). *TkSRPP3* and *TkSRPP4* expression declined after 12 weeks but increased again after 14 weeks. Similarly, *TkUGT80B1* expression increased over time, declined slightly after 12 weeks, and stayed constant between weeks 14 and 16 (**Figure 7B**). *TkSRPP6* expression was constant at low levels throughout the experiment (**Figure 7B**). The expression data reflected the high level of heterogeneity between individuals reported earlier (McAssey et al., 2016; Nowicki et al., 2019; Panara et al., 2018; Wieghaus et al., 2022). Our data demonstrated comparable temporal expression patterns for *TkUGT80B1* and *TkSRPP3/4/5*, but not *TkSRPP6*.

### 3.7 Cellular localization of TkSRPP6 and TkUGT80B1

**Figure 8:**
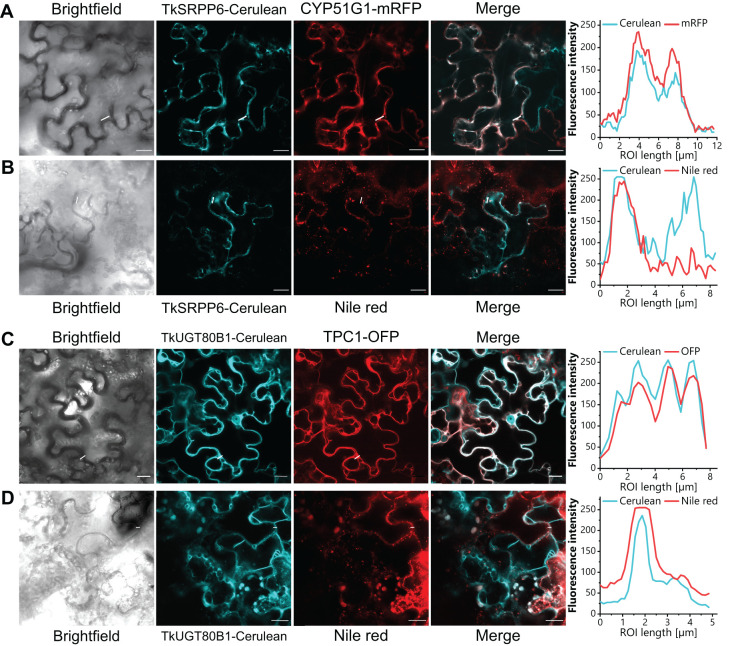
TkSRPP6 and TkUGT80B1 localize to the ER and tonoplast, respectively, and show affinity to LDs. *N. benthamiana* leaf epidermal cells expressing N-terminal Cerulean fusion constructs (cyan) and mRFP or OFP fusion subcellular markers are shown. **(A)** TkSRPP6-Cerulean and ER marker CYP51G1-mRFP. **(B)** TkSRPP6-Cerulean and Nile red signal representing LDs. **(C)** TkUGT80B1-Cerulean and tonoplast marker TPC1-OFP. **(D)** TkUGT80B1-Cerulean and Nile red signal representing LDs. For LD formation, Cerulean fusion constructs were co-expressed with *AtLEC2* and LDs were stained with the lipophilic fluorescent dye Nile red. Fluorescence intensities in regions of interest are depicted on the right. Scale bar = 20 µm.

The analysis of different latex phases by AE-MS provided crude data concerning the potential localization of TkSRPP6 and TkUGT80B1. For more detailed analysis, we expressed fusion proteins in *N. benthamiana* along with subcellular markers. We prepared constructs in which TkSRPP6 and TkUGT80B1 were C-terminally fused to the fluorescent reporter Cerulean, and transiently co-expressed them with ER and tonoplast markers. TkSRPP6-Cerulean fluorescence and the ER marker CYP51G1-mRFP (Bassard et al., 2012) overlapped almost completely (**Figure 8A**), whereas TkUGT80B1-Cerulean fluorescence largely coincided with the tonoplast marker TPC1-OFP (Batistič et al., 2010) (**Figure 8C**). Tk/TbSRPPs 3/4/5 were previously shown to be associated with rubber particles (Collins-Silva et al., 2012; Hillebrand et al., 2012), which are related to LDs, thus explaining the LD localization of TbSRPPs in *N. benthamiana* (Laibach et al., 2018). We therefore determined whether TkSRPP6 and TkUGT80B1 also associate with LDs by co-expressing the Cerulean fusion constructs with *AtLEC2*, encoding a transcription factor that promotes LD formation in leaves (Santos Mendoza et al., 2005). We then stained the LDs with the lipophilic fluorescent dye Nile red. We found that the Cerulean fluorescence profiles of TkSRPP6 and TkUGT80B1 described above included additional punctuate fluorescence that overlapped with the Nile red signal (**Figures 8B, D**). The affinity of these candidates for LDs, despite the absence of enrichment in the RP fraction in AE-MS experiments, suggests they interact with TkSRPP3/4/5 on the surface of rubber particles but in a conditional manner.

### 3.8 Glycosyltransferase activity of TkUGT80B1

**Figure 9:**
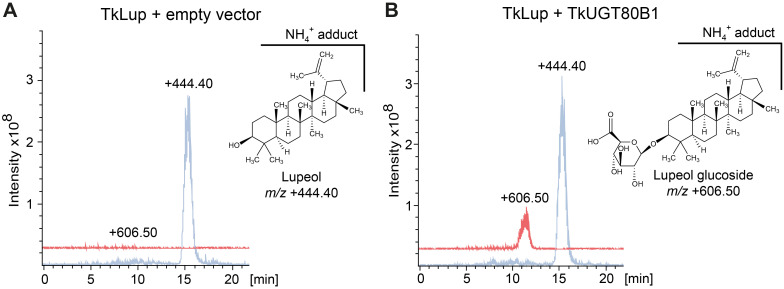
TkUGT80B1 glycosylates the triterpenoid lupeol in yeast. LC-MS chromatograms of extracts from yeast metabolically engineered for increased triterpenoid production (Bröker et al., 2018) expressing additionally **(A)** *lupeol synthase* (*TkLup*) and *TkUGT80B1* and **(B)** only *TkUGT80B1*. The signal at *m/z* 444.40 corresponds to the lupeol ammonium ion and *m/z* 606.50 to the ammonium ion of glucosylated lupeol that is only detectable when *TkLup* and *TkUGT80B1* are co-expressed. The chemical structure of lupeol and the predicted structure of the C_3_ glucosylated lupeol corresponding to *m/z* 606.50 are shown next to the chromatograms. Chromatograms of an additional control strain containing *TkLup* and an empty vector control are shown in **Supplementary Figure S5**.

Finally, we tested the predicted UGT activity of TkUGT80B1 in a yeast strain engineered for optimized pentacyclic triterpenoid synthesis and harboring a *T. koksaghyz* lupeol synthase gene (*TkLup*) (Bröker et al., 2018). Lupeol is a pentacyclic triterpenoid present in *T. koksaghyz* roots and NR, and is therefore a potential native substrate for TkUGT80B1 (Benninghaus et al., 2020; Pütter et al., 2019). Isoprenoid metabolites were extracted from yeast cultures and LC-MS chromatograms were compared to control strains either expressing *TkLUP* together with an empty vector or *TkUGT80B1* without *TkLup* (**Figure 9**; **Supplementary Figure S6**). We observed an additional peak (*m/z* +606.5) for yeast cells expressing *TkUGT80B1* and *TkLup* (**Figure 9B**). The mass corresponds to a positively charged lupeol hexose ammonium ion adduct, and thus indicates TkUGT80B1 has lupeol glycosylating activity. Based on the molecular structure of lupeol, we deduce that TkUGT80B1 is a C_3_-glycosyltransferase.

## 4 Discussion

The *T. koksaghyz* genome encodes 13 homology-based, full-length TkSRPPs (Lin et al., 2018, 2022), and their diverse sequences and expression profiles suggest non-redundant specialized functions in different tissues (**Figure 1**; **Table 1**). *TkSRPPs 3/4/5* are strongly expressed in the latex, so we sought interacting proteins that may contribute to NR biosynthesis and stress responses. The high constitutive levels of TkSRPP3/4/5 in latex indicate their requirement for basic processes without external stimuli, including rubber particle biogenesis, coating and stabilization. However, the presumably higher levels of TkSRPP3/4/5 protein following stress-induced transcriptional upregulation (Dong et al., 2023a; He et al., 2024; Laibach et al., 2018) indicate that the constitutive pool is insufficient to fulfil the extended functions needed in response to environmental changes, necessitating *de novo* protein synthesis. The presence of *N*-glycosylation and phosphorylation sites in TkSRPP3/4/5 indicates the proteins can be covalently modified, which may result in conformational and functional changes (Ha and Loh, 2012; Volkman et al., 2001). The different numbers of potential post-translational modification sites and distinct protein charges resulting from *TkSRPP* sequence divergence likely contribute to TkSRPP3/4/5 functional divergence represented by their separate interactomes.

### 4.1 TkSRPP3/4/5 interact with proteins related to isoprenoid and NR biosynthesis

**Figure 10:**
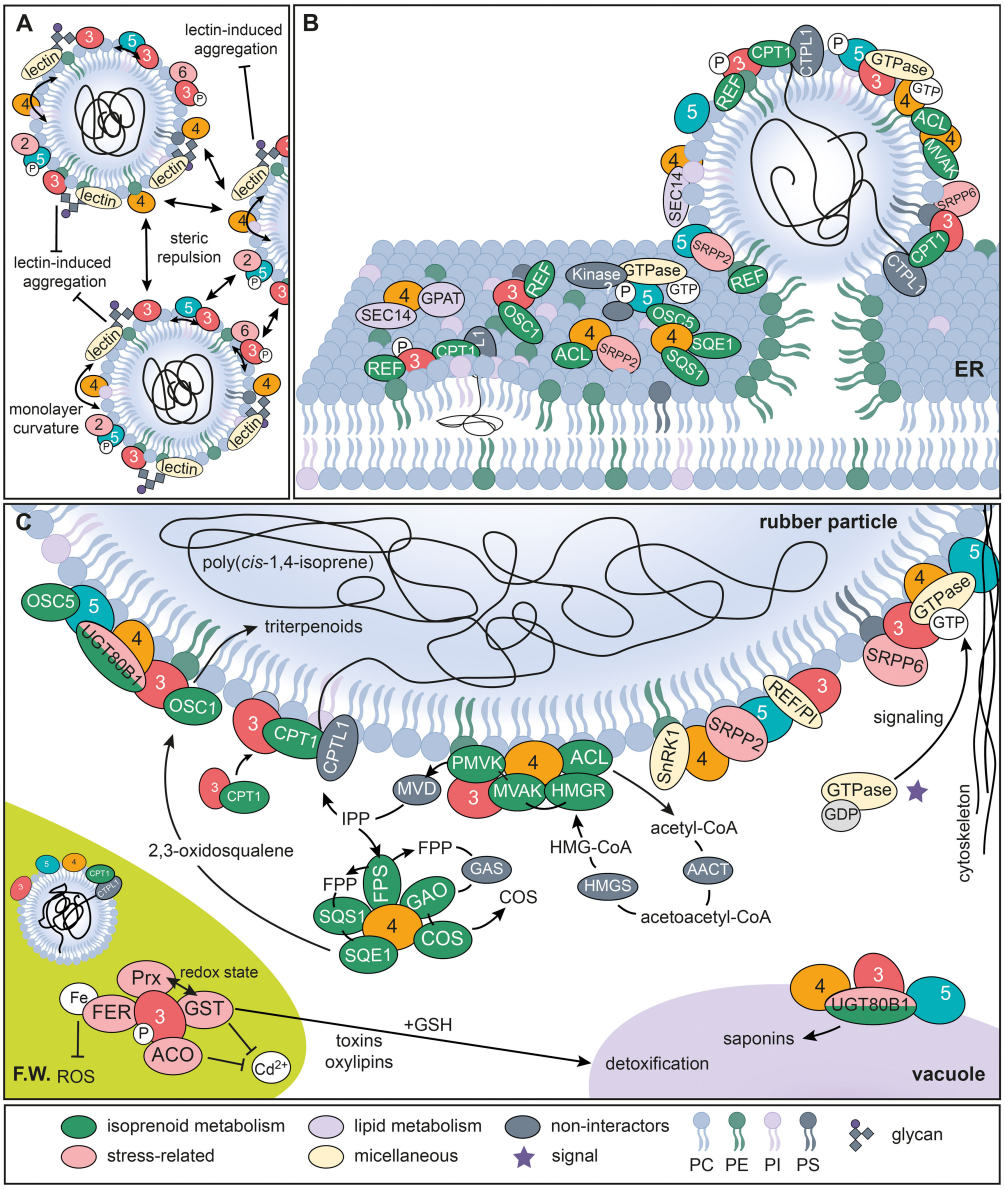
Models of TkSRPP3/4/5 potential protein interaction networks. **(A)** Model of processes involved in rubber particle stabilization and dispersity. **(B)** Illustration of potential TkSRPP3/4/5 protein interactions at the ER membrane initiating rubber particle formation. **(C)** Proposed protein interactions of TkSRPP3/4/5 at the surface of rubber particles and other cellular components. Black lines and arrows indicate molecule movement. T-shaped arrows indicate inhibitory effects. Two-sided arrows depict repulsion. TkSRPP3/4/5 names are shortened to their respective numbers. AACT, acetoacetyl-CoA thiolase; ACL, ATP-citrate synthase; COS, costunolid/costunolid synthase; CPT, *cis*-prenyltransferase; CPTL, *cis*-prenyltransferase-like; FER, ferritin; FPP, farnesyl diphosphate; FPS, farnesyl diphosphate synthase; FW, Frey-Wyssling complex; GAO, germacrene A oxidase; GDP, guanosine diphosphate; GPAT, glycerol-3-phosphate acyltransferase; GSH, glutathione sulfhydryl form; GST, glutathione S-transferase; HMGR, 3-hydroxy-3-methylglutaryl-CoA reductase; HMGS, 3-hydroxy-3-methylglutaryl-coenzyme A synthase; MVD, mevalonate diphosphate decarboxylase; MVK, mevalonate kinase; OSC, oxidosqualene cyclase; PC, phosphatidylcholine; PE, phosphatidylethanolamine; PI, phosphatidylinositol; Prx, peroxiredoxin; PS, phosphatidylserine; REF/PI, REF domain containing/perilipin-like related protein; PMVK, phosphomevalonate kinase; ROS, reactive oxygen species; SnRK1, Snf1-related protein kinase 1; SQS1, squalene synthase 1; SQE1, squalene epoxidase 1; UGT80B1, UDP-glycosyltransferase 80B1.

Our AE-MS experiments revealed distinct but overlapping interactomes for TkSRPP3/4/5 in whole latex and its three fractions. TkSRPP4 interacted with more proteins than the others and may function as a hub. TkSRPP3 and TkSRPP5 also interacted with each other, although enrichment was only observed from whole latex and IP, not from the RP fraction (**Supplementary Table S7**). SRPP heterodimers have also been reported for *T. brevicorniculatum* (Laibach et al., 2018). These findings suggest TkSRPPs can act cooperatively, in agreement with the additive effect of TbSRPPs 3/4/5 on artificial poly(*cis*-1,4-isoprene) body size and dispersity (Laibach et al., 2018). Lipid–protein interactions influence membrane composition (Harayama and Riezman, 2018) so the TkSRPP3/TkSRPP5 interaction may induce specific rearrangements in the lipid monolayer of rubber particles that promote the most stable lipid distribution, and/or enhance the steric repulsion assumed to be caused by SRPPs on the rubber particle surface (**Figure 10A**) (Hillebrand et al., 2012). TkSRPP3 and TkSRPP5 may also form complexes with their common interactors, including a REF family protein distantly related to a perilipin-4-like protein from the tobacco hawkmoth *Manduca sexta*, which was significantly less abundant in NR–depleted *T. koksaghyz* roots (Benninghaus et al., 2020). Perilipins are LD-associated proteins in animals that promote the formation and stability of LDs by regulating lipolysis (Griseti et al., 2024). Although this protein was not enriched from the RP, further analysis to determine its impact on rubber particles would be interesting, especially given its lack of interaction with TkSRPP4.

To understand the role of TkSRPP3/4/5 in NR biosynthesis beyond rubber particle stability, we screened the interactomes for further proteins related to NR and other isoprenoids (**Supplementary Table S7**). Many candidate TkSRPP interactors were found to differ in abundance when comparing proteins in the latex of *TkCPTL1*-RNAi plants and wild-type controls, suggesting a contribution to NR biosynthesis (Niephaus et al., 2019) (**Table 2**). Interestingly, TkSRPP3 interacted with TkCPT1 and TkREF, two well-known components of the NR biosynthesis machinery. The interaction with TkCPT1 supports the hypothesis that TkSRPPs affect TkCPT1 activity, causing the low NR content in *Tk/TbSRPP* RNAi plants (Collins-Silva et al., 2012; Hillebrand et al., 2012). Based on the proposed role of TbREF in rubber particle biogenesis (Laibach et al., 2015) and the recruitment of HbCPT6 from the cytosol to the ER by HbSRPP (Brown et al., 2017), TkSRPP3 may recruit TkCPT1 to ER sites where TkSRPP3 is in contact with other TkSRPPs and TkREF, thus modifying the lipid composition, helping TkCPT1 to channel nascent poly(*cis*-1,4-isoprene) chains between ER leaflets and favoring the formation of rubber particles (**Figure 10B**). This is supported by the reported interaction between HbSRPP and HbREF (Yamashita et al., 2016). The TkSRPP3/TkCPT1 interaction was detected from whole latex and the IP. Although NR-producing CPTs have mostly been identified in rubber particles (Dai et al., 2013; Schmidt et al., 2010b), the recruitment of TkCPT1 from the cytosol to the ER by TkSRPP3 may be the mechanism by which TkCPT1 becomes localized to this compartment.

TkSRPP3 and TkSRPP4 interacted with MVA pathway enzymes that provide the C_5_ building block IPP for NR polymerization (Pu et al., 2021; Salehi et al., 2021). This group comprised ATP-citrate synthase (TkACL1), TkHMGR, and mevalonate kinase (TkMVAK1) (all interacting with TkSRPP4), as well as TkMVAK10 (interacting with TkSRPP3) and phosphomevalonate kinase 3 (TkPMVK3, interacting with TkSRPPs 3 and 4). TkSRPP4 also interacted with an FPP synthase (TkFPS1), which provides the most likely starter molecule for NR polymerization *in vivo* (Puskas et al., 2006; Tanaka et al., 1996; Xie et al., 2008). TkSRPP3 and TkSRPP4 may therefore be required to ensure an efficient supply of metabolic precursors to the *cis*PT complex by forming the structural components of a NR metabolon (**Figure 10C**), although metabolic channeling experiments would be required for confirmation (Srere, 1972, 1987; Zhang and Fernie, 2021). This model is supported by the enrichment of TkMVAK1, a cytosolic enzyme (Cho et al., 2022; Niu et al., 2021; Simkin et al., 2011), from the RP by TkSRPP4 (**Supplementary Data S2** in **Supplementary Data Sheet 1**). TkSRPP3 may also stabilize the complex by interacting with TkSRPP5, which in turn interacts with multiple proteins related to the ‘microtubule cytoskeleton’ (**Figure 4**). TkHMGR was enriched from the IP, whereas HMGRs are usually found in the ER (Hampton et al., 1996; Leivar et al., 2005). However, the presence of eight *HMGR* paralogs in the *T. koksaghyz* genome (Lin et al., 2022) and the differential expression of *TbHMGRs* (Van Deenen et al., 2012) suggests there is scope for functional specialization, with at least one HMGR associated with NR synthesis (Campos and Boronat, 1995; Chappell, 1995; Leivar et al., 2005; Lin et al., 2022; McCaskill and Croteau, 1998). The involvement of this TkHMGR paralog in the supply of precursors for NR synthesis is supported by its depletion in *TkCPTL1*-RNAi plants (**Table 2**) (Niephaus et al., 2019).

Intriguingly, TkSRPP4 and TkSRPP5 also interacted with plastidial methylerythritol (MEP) pathway enzymes, an alternative route to IPP (Vranová et al., 2012). Specifically, TkSRPP4 interacted with 1-deoxy-d-xylulose-5-phosphate synthase (TkDXS8) whereas both TkSRPP4 and TkSRPP5 interacted with 4-hydroxy-3-methylbut-2-en-1-yl diphosphate reductase (TkHDS1). Although latex does not contain genuine chloroplasts, MEP pathway enzymes and low levels of corresponding mRNAs have been detected in *T. koksaghyz* latex before (Lin et al., 2018; Niephaus et al., 2019). Additionally, specialized plastids known as Frey-Wyssling (F.W.) complexes (Frey-Wyssling, 1929) have been described in *T. koksaghyz* and *H. brasiliensis* latex (Abdul Ghaffar, 2017; Dickenson, 1969; Gomez and Hamzah, 1989; Moir, 1959), and these compartments may comprise the MEP pathway in latex. However, TkDXS8 and TkHDS1 were enriched from the RP, IP and whole latex but not the PP where F.W. complexes would be presumed. This may reflect the disruption of F.W. complexes during processing or the liberation of MEP pathway enzymes by another mechanism, although we would also expect interactions with TkSRPP3 in this scenario due to the significant number of chloroplast-related TkSRPP3 interactors. Feeding experiments in *H. brasiliensis* showed that in this species the MEP pathway contributes to carotenoid rather than NR biosynthesis in latex (Sando et al., 2008). Still, the interactions of TkSRPP3/4/5 with different isoprenoid precursor pathways raise the possibility that they drive IPP flux towards NR synthesis and provide more evidence that TkSRPP4 is a hub protein whereas TkSRPP3 and TkSRPP5 are more specialized.

TkSRPP4 also appears to engage with isoprenoid pathways downstream of FPP by interacting with enzymes involved in sesquiterpene lactone and triterpenoid biosynthesis (González-Coloma et al., 2011; Huber et al., 2015, 2016; Padilla-Gonzalez et al., 2016), the latter including TkSQS1, TkSQE1 and oxidosqualene cyclase 5 (TkOSC5). TkSRPP3 and TkSRPP5 interacted with TkOSC1 and TkOSC5, respectively. Both are latex-specific enzymes and TkOSC1 produces at least four different triterpenoids from 2,3-oxidosqualene, most likely provided by TkSQE1 (Pütter et al., 2019; Unland et al., 2018), whereas TkOSC5 did not produce any triterpenoids in *N. benthamiana* (Pütter et al., 2019). The transcriptional co–regulation of *TkSQS1*, *TkSQE1* and *TkOSC1* facilitates tight metabolic coupling, and TkSQS1 colocalizes with TkSQE1 in the ER of *N. benthamiana* (Unland et al., 2018). Given the presence of transmembrane domains in both proteins, their interaction with TkSRPP4 in the IP may reflect their translocation caused by the phase separation procedure. TkOSC1 has yet to be detected in the ER following heterologous expression (Pütter, 2017). Therefore, TkSRPP3 and TkSRPP4 may cooperatively mediate the assembly of these three consecutive enzymes in the cytosol, ER or on rubber particles for the efficient synthesis of bioactive triterpenoids, given that other OSCs were shown to localize to LDs in yeast (Milla et al., 2003) and triterpenoids are the most abundant non-polyisoprenoid component in separated NR from *T. koksaghyz* (Pütter et al., 2019). TkOSC5 may need to interact with TkSRPPs to maintain stability or activity. TkSRPP3/4/5 could thus affect the quality of NR as an industrial raw material because the triterpenoid content is proposed to influence the physical properties of the polymer (Xu et al., 2017).

### 4.2 TkSRPP3/4/5 interactomes suggest their involvement in rubber particle biogenesis, integrity and dispersity

#### 4.2.1 TkSRPP4 protein interactions may contribute to rubber particle biogenesis from the ER

So far, TkSRPP3/4/5 have only been found associated with rubber particles in latex (Collins-Silva et al., 2012) but their presence in different latex phases and Tb/TkSRPP localization in *N. benthamiana* suggest they may also be present in the ER and cytosol (He et al., 2024; Laibach et al., 2018). In support of that, exclusive TkSRPP4 interactors were related to the ER membrane (**Figure 4**). TkSRPP4 is therefore likely to be the most important component of ER-related SRPP functions, whereas TkSRPP3 and TkSRPP5 cooperate with other TkSRPPs in the ER. These processes could include the transmembrane transport of inorganic molecules because a related GO term was also enriched among TkSRPP4 interactors (**Figure 4**). Glycerol-3-phosphate acyltransferase (GPAT) was enriched with TkSRPP4 from the PP and was downregulated in *TkCPTL1*-RNAi plants (Niephaus et al., 2019). GPATs catalyze the transfer of an acyl group to the *sn*-1 position of glycerol-3-phosphate leading to the formation of lysophosphatidic acid, and this can be acylated further to phosphatidic acid, the common precursor of other phospholipids and TAGs (Jayawardhane et al., 2018; Testerink and Munnik, 2011). Phosphatidic acid is also important for ROS signaling under biotic stress (Gong et al., 2024). The conversion of lysophosphatidic to phosphatidic acid may cause negative monolayer curvature that could play a role in rubber particle biogenesis (Kooijman et al., 2003). GPAT influences TAG biosynthesis (Gidda et al., 2009; Shockey et al., 2015) and confers tolerance against freezing stress, which often affects *T. koksaghyz* (Kasapoğlu et al., 2024; Sui et al., 2007a, 2007). Some GPATs also contain a phosphatase domain, and *sn*-2-monoacylglycerol was the major product of Arabidopsis GPATs (W. Yang et al., 2010). They have been found in different cellular compartments (Fernández-Santos et al., 2020; Gidda et al., 2009; Kasapoğlu et al., 2024; Sun et al., 2021) and contribute to LD formation, which was associated with their role in TAG biosynthesis (Gao et al., 2013; Wilfling et al., 2013). Rubber particles are not known to store TAGs, but GPAT may contribute to rubber particle budding from the ER via its interaction with TkSRPP4 (**Figure 10B**). A phosphatase activity and supply of phosphatidic acid could drive phospholipid synthesis and incorporation into the rubber particle or ER membrane. GPAT is therefore an interesting candidate for further analysis of lipid modifications that contribute to rubber particle formation and stress tolerance. Two SEC14 cytosolic factors containing CRAL-TRIOL domains prevalent in lipid-binding proteins (Panagabko et al., 2003) were also identified as TkSRPP4 interactors and were among the proteins downregulated in *TkCPTL1*-RNAi plants (Niephaus et al., 2019). SEC14 proteins are PI/phosphatidylserine transfer proteins that modulate membrane identity, including lipid raft formation (Curwin et al., 2013; Montag et al., 2023). TkSRPP4 may cooperate with these proteins in the ER to accumulate the proteins and lipids needed for rubber particle formation (**Figure 10B**).

#### 4.2.2 TkSRPP3 and TkSRPP4 protein interactions at the rubber particle may contribute to its integrity and dispersity

The identification of several lipid-modifying enzymes among the TkSRPP3 RP interactors indicates they act on monolayer lipids and the rubber particle lipid composition may be continuously modified and rearranged. The fact that all TkSRPP3 RP interactors are exclusive to this paralog demonstrates TkSRPP functional divergence and potential functional specialization of TkSRPP3 at the rubber particle, which could be mediated by its specific physicochemical properties. TkSRPP4 interactors enriched from the RP comprised several proteins associated with ubiquitination/de-ubiquitination and ubiquitin-dependent proteasomal degradation, suggesting a role in rubber particle protein homeostasis, which is probably required to maintain particle integrity and efficient NR biosynthesis. Several other TkSRPP4 RP interactors were related to sucrose non-fermenting 1 (Snf1) and its plant homolog Snf1-related protein kinase 1 (SnRK1), a major regulator of developmental plasticity including lipid biosynthesis (Jamsheer K et al., 2021). These proteins inactivate HMGR (Robertlee et al., 2017; Sugden et al., 1999) and a key enzyme in PC biosynthesis (Caldo et al., 2019), and also regulate TAG biosynthesis (Zhai et al., 2017). Therefore, the presence of these kinases on the rubber particle surface and their interactions with TkSRPP4 may also influence the lipid composition of the monolayer and the stored NR and triterpenoids.

Further, the interaction of TkSRPP4 with a lectin downregulated in NR-depleted *TkCPTL1*-RNAi plants could play a role in rubber particle dispersity (**Table 2**) (Niephaus et al., 2019). A latex lectin in *H. brasiliensis* that induces rubber particle aggregation is inhibited by binding to a glycosylated SRPP and the *N*-acetylglucosamine residue of the SRPP was necessary for binding, which is typical for lectins (Rüdiger and Gabius, 2002; Wititsuwannakul et al., 2008). The TkSRPP4 and TkSRPP3 interactomes also featured additional lectins, and the single *N*-glycosylation site found in TkSRPP3 and TkSRPP4 suggests a similar role in rubber particle dispersity that could synergize with the induced steric repulsion (**Figure 10A**).

The interaction of TkSRPP4 with a homolog of isoprenylcysteine α–carbonyl methylesterase-like 2 (ICMEL2) that was also downregulated in *TkCPTL1*-RNAi plants (**Table 2**) (Niephaus et al., 2019) may also play a role in rubber particle biogenesis and integrity. Proteins can be C-terminally prenylated by the addition of farnesyl or geranylgeranyl groups to a cysteine to increase their membrane affinity, usually followed by methylation in the ER, which can be reversed by ICMEs (Clarke, 1992; Crowell, 2000; Lan et al., 2010; Zhang and Casey, 1996). The methylation status can affect protein–lipid interactions and prenylation can affect protein–protein interactions (Crowell, 2000; Hancock et al., 1991; Kuroda et al., 1993; Sapperstein et al., 1994; Zhang and Casey, 1996). Such modifications may therefore be important for protein recruitment to the ER or rubber particles mediated by TkSRPP4/ICMEL2 complexes.

#### 4.2.3 TkSRPP5 interaction with GTPases may promote rubber particle formation

Proteins that undergo prenylation for membrane targeting include GTPases (Chavrier et al., 1991; Hancock et al., 1991; Kuroda et al., 1993), which were enriched among the exclusive TkSRPP5 interactors and part of TkSRPP3/4/5 RP interactomes (**Figure 4**; **Supplementary Data S1–S3** in **Supplementary Data Sheet 1**). GTPases regulate multiple cellular processes, especially signal transduction and vesicle transport, but also play a major role in immune responses (Kawano et al., 2014; Nielsen, 2020). They switch between inactive GDP-bound and active GTP-bound states, in which they are prenylated and associate with membranes, allowing them to engage with effector proteins (Grosshans et al., 2006; Kawano et al., 2014). TkSRPP5 interactions with GTPases may regulate GTP/GDP binding or membrane interactions, or the GTPases may recruit TkSRPP5 as an effector protein to change membrane lipid distribution. GTPases also recruit proteins that induce vesicle formation (Huang et al., 2001; Kawasaki et al., 2005; Spang, 2008; Springer et al., 1999) and the recruitment of TkSRPP5 and other proteins could thus induce rubber particle budding from the ER (**Figure 10B**), aligning with the identification of GTPases on rubber particles from *H. brasiliensis* (Nielsen et al., 2008; Yamashita et al., 2016). The interaction of TkSRPP5 with a putative methyltransferase that was downregulated in *TkCPTL1*-RNAi lines (Niephaus et al., 2019) could indicate a role in the methylation of prenylated proteins such as GTPases. Therefore, TkSRPP4 and TkSRPP5 may regulate the methylation/demethylation of prenylated latex proteins that affect membrane association and protein interactions. Further, TkSRPP5 interactions with lipid-modifying proteins (**Figure 4**) could help to establish the membrane conditions needed for rubber particle biogenesis. Beyond that, GO enrichment analysis revealed that exclusive TkSRPP5 interactors were enriched for the term ‘microtubule cytoskeleton’ (**Figure 4**). Such proteins are present on rubber particles in *H. brasiliensis* (Dai et al., 2013) and the interaction of TkSRPP5 with those proteins may facilitate the transport of rubber particles along the cytoskeleton to the vacuole, which contains many cytoskeleton-related proteins in the tonoplast (Carter et al., 2004) and stores rubber particles in *T. koksaghyz* (Abdul Ghaffar, 2017).

### 4.3 Further evidence for the involvement of TkSRPPs and TkSRPP heterodimers in stress responses

Common TkSRPP3/4/5 interactors were related to membrane and vesicular trafficking, lipid metabolism and stress responses (**Supplementary Table S6**). TkSRPP3/4/5 may therefore promote stress tolerance by affecting lipid modification, the proteolytic cleavage of pathogen-derived proteins, the inhibition of pathogen-derived proteases and/or cellular adaptations by membrane trafficking. Rab7 proteins, for example, are involved in vacuolar trafficking and improve abiotic stress tolerance when overexpressed (Mazel et al., 2004; Rodriguez-Furlan et al., 2023; Tripathy et al., 2017).

As the protein TkSRPP7 was enriched from whole latex and the IP by TkSRPP3/4/5, despite the relatively low *TkSRPP7* transcript levels in the latex of 10-week-old plants (**Figure 1B**) and the lack of evidence showing its association with rubber particles, this paralog may interact with TkSRPP3/4/5 elsewhere in the laticifers as part of stress-related processes. This may also explain why TkSRPP2 interacted with TkSRPP4 and TkSRPP5 in all latex fractions, and why TkSRPP1 interacted with TkSRPP3 in whole latex (**Supplementary Table S7**). The roles of these TkSRPPs in stress responses are supported by their transcriptional upregulation following treatment with MeJA or the overexpression of *TkMYC2* (He et al., 2024; Wu et al., 2024). TkSRPPs may also form multimers to fulfil their functions, as suggested for HbSRPP (Wititsuwannakul et al., 2008).

The overrepresentation of proteins associated with the chloroplast stroma and thylakoids among exclusive TkSRPP3 interactors was striking (**Figure 4**). TkSRPP3 and its interactors related to the chloroplast stroma and thylakoids may associate with the plastid-like F.W. complexes, explaining why most of these proteins were enriched from the PP. Notably, rubber particles were also observed within plastid-like structures in *T. koksaghyz* laticifers (Abdul Ghaffar, 2017), so TkSRPP3 and its chloroplast-related latex interactors may also be connected with such rubber particles. The presence of ferritins, peroxiredoxins, glutathione Ѕ-transferase (GST) and aconitate hydratase among those TkSRPP3 interactors indicate roles in antioxidant defense, redox regulation and detoxification (Briat, 1996; Dietz, 2003; Kumar et al., 2024; Kumar and Trivedi, 2018; Moeder et al., 2007; Pascual et al., 2021). Ferritin inhibits the formation of reactive oxygen species (ROS) by Fe (Halliwell and Gutteridge, 1984; Kroh and Pilon, 2020; Ravet et al., 2009), peroxiredoxins are antioxidants that mediate redox-dependent signaling (Liebthal et al., 2018; Sevilla et al., 2015), and GSTs counter oxidative stress by conjugating glutathione (Cozza et al., 2017; Dixon and Edwards, 2009; Labrou et al., 2015; Nianiou-Obeidat et al., 2017; Wagner et al., 2002). Overexpression of these genes confers abiotic and biotic stress tolerance (Deák et al., 1999; Lo Cicero et al., 2017; Roxas et al., 2000; Wang et al., 2023; Xiao et al., 2023; Zang et al., 2017), and their endogenous promoters are responsive to stress and phytohormones (Dellagi et al., 2005; García Mata et al., 2001; Horling et al., 2002, 2003; Marrs, 1996; Tiwari et al., 2016). GST and aconitate hydratase are also associated with cadmium stress tolerance, a significantly enriched process among TkSRPP3 interactors (**Figure 4**) that has also been experimentally linked to REF proteins (Dixit et al., 2011; Kim et al., 2011; Liu et al., 2013, 2016; Zhou et al., 2019). These interactors may therefore form a TkSRPP3-dependent network of stress tolerance effectors within F.W. complexes (Cerveau et al., 2016; Manevich et al., 2004), as illustrated in **Figure 10C**. The large number of kinases among the TkSRPP3 interactors suggests the effector network is regulated by kinase cascades, supported by multiple phosphorylation sites on TkSRPP3. Finally, the glutathione peroxidase activity of GSTs prevents lipid oxidation (Dixon et al., 2009; Dixon and Edwards, 2009; Ohkama-Ohtsu et al., 2011) and could be particularly valuable in latex that contains diverse lipids, many associated with bioactive properties (Bae et al., 2020; González-Coloma et al., 2011; Pütter et al., 2019).

The involvement of TkSRPP5 in stress response is supported by the enrichment of interactors associated with responses to different stimuli (**Figure 4**). The activation of TkSRPP5 in response to such stimuli could be achieved by phosphorylation at the six predicted phosphorylation sites or by *N*-glycosylation (**Figure 1E**).

### 4.4 TkSRPP6 forms heteromeric complexes with TkSRPP4 and TkSRPP5

TkSRPP6 was one of two candidate interactors that we chose for further analysis, due to its isolated genomic locus and phylogenetic clustering with stress-related proteins from non-rubber-producing plants rather than other TkSRPPs (**Figure 5C**). The *TkSRPP6* gene was also expressed at lower levels than *TkSRPP3/4/5* in all tissues (and in latex over time) (**Figures 1**, **7**). SUY2H results confirmed that TkSRPP6 interacted with TkSRPP4 and TkSRPP5, but not TkSRPP3 (**Figure 5A**). The interaction of TkSRPP6 with the abundant latex proteins TkSRPP4 and TkSRPP5, despite their different molecular characteristics, was striking. The identification of MYC2-binding sites in the *TkSRPP6* promoter suggested inducible expression, supported by the transcriptional induction observed after MeJA treatment and *TkMYC2* overexpression (**Figures 1**, **5D**) (*TkSRPP1* in He et al., 2024; *TkSRPP7* in Wu et al., 2024). This induction might be transient, as shown for MeJA (He et al., 2024), and would therefore not show up in our qPCR data for wild-type plants. Stress-induced expression is a common feature of the REF family and is supported by the homology of TkSRPP6 to stress-related proteins (**Figure 5C**). The heterologous expression of TkSRPP6 in *N. benthamiana* resulted in localization to the ER and LDs, matching its enrichment from the RP by TkSRPP3 (**Figures 8A, B**; **Table 3**). A recent study (He et al., 2024) suggested TkSRPP6 (named TkSRPP1 therein) was localized to the cytosol, plasma membrane and chloroplast, but the authors did not induce LD formation nor did they use plasma membrane and ER markers to confirm their assumptions. We observed no plastid signals for our TkSRPP6-Cerulean fusion protein (**Figures 8A, B**). TbSRRPs were found to be localized to the cytosol (Laibach et al., 2018) and TkSRPP6 enrichment from the IP supports a cytosolic localization. We conclude that TkSRPP6 may have affinities for different cellular compartments that may change depending on specific conditions.

The localization of TbSRPPs 4 and 5 and TkSRPP6 together with indications of inducible gene expression suggest that TkSRPP6 may interact at basal levels with TkSRPP4 and TkSRPP5 on the surface of the ER and rubber particles, but predominantly after its short-term transcriptional induction in response to various stimuli. These interactions most likely play a role in stress tolerance, and TkSRPP6 could engage with established complexes formed by TkSRPP4/TkSRPP5. TkSRPP6 may respond to stress not only in the latex but also in green tissues as its transcript levels were comparable low in all tissues.

### 4.5 TkSRPP3 and TkSRPP5 interact with TkUGT80B1 potentially contributing to plant stress tolerance by producing triterpenoid saponins

TkUGT80B1 was the second candidate selected for in depth characterization because glycosyltransferases have not been described in dandelion latex before and glycosides are involved in stress responses (Augustin et al., 2011; Rogowska and Szakiel, 2020). An independent co-IP assay confirmed that TkUGT80B1 interacts with TkSRPP3 and TkSRPP5 (**Figure 6A**). The interactions were further corroborated by the similar temporal expression patterns of *TkUGT80B1* and *TkSRPP3/4/5* in latex (**Figure 7**) (Ge et al., 2001; Jansen et al., 2002; von Mering et al., 2002).

The predicted glycosyltransferase activity of TkUGT80B1 was confirmed for the C_3_ position of the triterpenoid lupeol in yeast, with UDP-glucose as the most likely sugar donor (**Figure 9**). This was supported by the presence of glutamine in the C-terminal UDPGT motif, which is conserved among glucosyltransferases but not galactosyltransferases (**Figure 6D**) (Kubo et al., 2004). Accordingly, we have identified the first enzyme from the latex of *T. koksaghyz* that produces a triterpenoid saponin and have provided first evidence for the presence of these compounds in dandelions. Given the low substrate specificities of UGTs (Vogt and Jones, 2000), the glycosylation of additional, structurally similar triterpenoids in *T. koksaghyz* latex is likely. The heterologous expression of TkUGT80B1 did not result in the glycosylation of yeast sterols when sterol synthesis was repressed (**Figure 9**). It is unclear whether TkUGT80B1 can also utilize phytosterol substrates, as described for its homologs (Stucky et al., 2014), or other lipids in latex. The analysis of Arabidopsis UGTs indicated that AtUGT80B1 is not required for the synthesis of major steryl glucosides but rather for the production of minor glucosides (Stucky et al., 2014). Accordingly, TkUGT80B1 may glycosylate triterpenoids rather than major membrane sterols in accordance with its predominant expression in latex (**Figure 6E**). Adaptation to freezing stress was inhibited in *atugt80b1* knockout plants, and was potentially related to low levels of sterol glycosides, the products of AtUGT80B1 (Mishra et al., 2015). It would be interesting to determine whether triterpenoid saponins have a similar positive effect on freezing tolerance in *T. koksaghyz* because the lipid–rich latex in its roots has already been proposed to act as an anti-freezing protectant during extremely cold winters, which are common in its native habitat.

The presence of several stress-related *cis*-acting regulatory elements in the *TkUGT80B1* promoter, including binding sites for WRKY transcription factors, suggests the gene is transcriptionally regulated in response to biotic stress (**Figure 6D**). WRKY transcription factors can affect defense response positively as well as negatively (Javed and Gao, 2023; Wani et al., 2021), but given the reported positive correlation between SRPPs and stress tolerance (Kim et al., 2016, 2012; Laibach et al., 2018; Seo et al., 2010), TkUGT80B1 is also likely to improve stress tolerance, although this should be investigated in more detail.

Saponins are stored in the vacuole (Kesselmeier and Urban, 1983; Mylona et al., 2008; Urban et al., 1983). *TkUGT80B1* expression in *N. benthamiana* suggested tonoplast localization (**Figure 8C**) and the enrichment of TkUGT80B1 from the PP by TkSRPP3 supports this finding. Enrichment from the IP by TkSRPP4 and TkSRPP5 may reflect the rupture of vacuoles during phase separation. Tonoplast localization may allow the immediate storage of nascent saponins in the vacuole. TkSRPP3 and TkSRPP5 could mediate the transfer of saponins from their biosynthetic enzyme to a transporter by linking both proteins. The transport of saponins to the vacuole may be mediated by ABC-type transporters (Kato et al., 2022; Ramilowski et al., 2013) and two proteins with homology to ABC-type transporters were identified as TkSRPP4 interactors, one of which also interacted with TkSRPP5. Interactions between TkSRPP4/TkSRPP5 and these transporters could also promote saponin efflux from the vacuole to promote stress tolerance. Localization studies in *N. benthamiana* showed that TkUGT80B1 can also accumulate in LDs, suggesting it might be located on the surface of rubber particles and interact with TkSRPPs there. The interaction between TkSRPPs and TkUGT80B1 may also promote the metabolic flux towards triterpenoid saponin synthesis by linking the triterpenoid synthesizing TkOSC1/5 with the glycosylating TkUGT80B1. TkSRPP3 and TkSRPP5 could further recruit either TkUGT80B1 or specific lipid substrates to direct glycoside synthesis and ultimately modify the composition of membranes in response to environmental conditions.

## 5 Conclusion

Our study sheds light on the *SRPP* gene family in *T. koksaghyz* and presents a comprehensive analysis of the protein interaction partners of the major latex proteins TkSRPP3/4/5. We identified protein interactions that suggest TkSRPP3/4/5 contribute directly to increased stress tolerance as well as rubber particle biogenesis and integrity. Two candidates were characterized at the molecular level, revealing the first evidence for saponin synthesis in *T. koksaghyz* latex and linking it with TkSRPP3 and TkSRPP5. Our data contribute to the functional differentiation between TkSRPP paralogs and demonstrate unexpected interactions that will help to further identify the network of proteins linking TkSRPPs, stress responses and NR biosynthesis. These new insights into the complexity of latex will eventually help to establish commercially feasible rubber crops.

## Data Availability

MS raw data are available for review at the following URL (https://repository.jpostdb.org/preview/48789265366a7c7a166d94) and will be made publicly available under the identifier JPST003234 upon publication. All other data presented in this report are available either in the supplemental tables, figures or data, or in publicly available databases.

## References

[B1] Abdul Ghaffar M. A. B. (2017). Rubber Particle Ontogeny in Taraxacum kok-saghyz (USA: Ohio State University). Available at: http://rave.ohiolink.edu/etdc/view?acc_num=osu1512031318000982.

[B2] Agatep R. Kirkpatrick R. D. Parchaliuk D. L. Woods R. A. Gietz R. D. (1998). Transformation of Saccharomyces cerevisiae by the lithium acetate/single-stranded carrier DNA/polyethylene glycol protocol. Tech. Tips Online 3, 133–137. doi: 10.1016/s1366-2120(08)70121-1

[B3] Alexa A. Rahnenfuhrer J. (2022). *topGO: Enrichment Analysis for Gene Ontology* (R package version 2.50.0).

[B4] Augustin J. M. Kuzina V. Andersen S. B. Bak S. (2011). Molecular activities, biosynthesis and evolution of triterpenoid saponins. Phytochemistry 72, 435–457. doi: 10.1016/J.PHYTOCHEM.2011.01.015 21333312

[B5] Bae S. W. Jung S. Choi S. C. Kim M. Y. Ryu S. B. (2020). Lipid Composition of Latex and Rubber Particles in Hevea brasiliensis and Taraxacum kok-saghyz. Mol. (Basel Switzerland) 25, 5110. doi: 10.3390/molecules25215110 PMC7662343 33153210

[B6] Bassard J. E. Mutterer J. Duval F. Werck-Reichhart D. (2012). A novel method for monitoring the localization of cytochromes P450 and other endoplasmic reticulum membrane associated proteins: A tool for investigating the formation of metabolons. FEBS J. 279, 1576–1583. doi: 10.1111/j.1742-4658.2011.08312.x 21851555

[B7] Batistič O. Waadt R. Steinhorst L. Held K. Kudla J. (2010). CBL-mediated targeting of CIPKs facilitates the decoding of calcium signals emanating from distinct cellular stores. Plant J. 61, 211–222. doi: 10.1111/J.1365-313X.2009.04045.X 19832944

[B8] Benninghaus V. A. Van Deenen N. Müller B. Roelfs K. U. Lassowskat I. Finkemeier I.. (2020). Comparative proteome and metabolome analyses of latex-exuding and non-exuding Taraxacum koksaghyz roots provide insights into laticifer biology. J. Exp. Bot. 71, 1278. doi: 10.1093/JXB/ERZ512 31740929 PMC7031084

[B9] Berthelot K. Lecomte S. Estevez Y. Peruch F. (2014). Hevea brasiliensis REF (Hev b 1) and SRPP (Hev b 3): An overview on rubber particle proteins. Biochimie 106, 1–9. doi: 10.1016/J.BIOCHI.2014.07.002 25019490

[B10] Briat J. F. (1996). Roles of ferritin in plants. J. Plant Nutr. 19, 1331–1342. doi: 10.1080/01904169609365202

[B11] Bröker J. N. Müller B. van Deenen N. Prüfer D. Schulze Gronover C. (2018). Upregulating the mevalonate pathway and repressing sterol synthesis in Saccharomyces cerevisiae enhances the production of triterpenes. Appl. Microbiol. Biotechnol. 102, 6923–6934. doi: 10.1007/s00253-018-9154-7 29948122 PMC6096838

[B12] Brown D. Feeney M. Ahmadi M. Lonoce C. Sajari R. Di Cola A.. (2017). Subcellular localization and interactions among rubber particle proteins from Hevea brasiliensis. J. Exp. Bot. 68, 5045–5055. doi: 10.1093/JXB/ERX331 29036360 PMC5853894

[B13] Caldo K. M. P. Xu Y. Falarz L. Jayawardhane K. Acedo J. Z. Chen G. (2019). Arabidopsis CTP:phosphocholine cytidylyltransferase 1 is phosphorylated and inhibited by sucrose nonfermenting 1–related protein kinase 1 (SnRK1). J. Biol. Chem. 294, 15862. doi: 10.1074/JBC.RA119.008047 31439667 PMC6816107

[B14] Campos N. Boronat A. (1995). Targeting and topology in the membrane of plant 3-hydroxy-3-methylglutaryl coenzyme A reductase. Plant Cell 7, 2163–2174. doi: 10.1105/TPC.7.12.2163 8718626 PMC161070

[B15] Cantalapiedra C. P. Herņandez-Plaza A. Letunic I. Bork P. Huerta-Cepas J. (2021). eggNOG-mapper v2: Functional Annotation, Orthology Assignments, and Domain Prediction at the Metagenomic Scale. Mol. Biol. Evol. 38, 5825–5829. doi: 10.1093/MOLBEV/MSAB293 34597405 PMC8662613

[B16] Cao X. W. Yan J. Lei J. L. Li J. Zhu J. B. Zhang H. Y. (2017). *De novo* Transcriptome Sequencing of MeJA-Induced Taraxacum koksaghyz Rodin to Identify Genes Related to Rubber Formation. Sci. Rep. 7, 1–13. doi: 10.1038/s41598-017-14890-z 29146946 PMC5691164

[B17] Carter C. Pan S. Zouhar J. Avila E. L. Girke T. Raikhel N. V. (2004). The vegetative vacuole proteome of Arabidopsis thaliana reveals predicted and unexpected proteins. Plant Cell 16, 3285. doi: 10.1105/TPC.104.027078 15539469 PMC535874

[B18] Cerveau D. Kraut A. Stotz H. U. Mueller M. J. Couté Y. Rey P. (2016). Characterization of the Arabidopsis thaliana 2-Cys peroxiredoxin interactome. Plant Sci. 252, 30–41. doi: 10.1016/J.PLANTSCI.2016.07.003 27717466

[B19] Chappell J. (1995). Biochemistry and molecular biology of the isoprenoid biosynthetic pathway in plants. Annu. Rev. Plant Physiol. Plant Mol. Biol. 46, 521–547. doi: 10.1146/annurev.pp.46.060195.002513

[B20] Chavrier P. Gorvel J. P. Stelzer E. Simons K. Gruenberg J. Zerial M. (1991). Hypervariable C-termmal domain of rab proteins acts as a targeting signal. Nature 353, 769–772. doi: 10.1038/353769a0 1944536

[B21] Cho S. H. Tóth K. Kim D. Vo P. H. Lin C. H. Handakumbura P. P.. (2022). Activation of the plant mevalonate pathway by extracellular ATP. Nat. Commun. 13, 450. doi: 10.1038/S41467-022-28150-W 35064110 PMC8783019

[B22] Clarke S. (1992). Protein isoprenylation and methylation at carboxyl-terminal cysteine residues. Annu. Rev. Biochem. 61, 355–386. doi: 10.1146/ANNUREV.BI.61.070192.002035/CITE/REFWORKS 1497315

[B23] Collins-Silva J. Nural A. T. Skaggs A. Scott D. Hathwaik U. Woolsey R.. (2012). Altered levels of the Taraxacum kok-saghyz (Russian dandelion) small rubber particle protein, TkSRPP3, result in qualitative and quantitative changes in rubber metabolism. Phytochemistry 79, 46–56. doi: 10.1016/j.phytochem.2012.04.015 22609069

[B24] Cornish K. Wood D. F. Windle J. J. (1999). Rubber particles from four different species, examined by transmission electron microscopy and electron-paramagnetic-resonance spin labeling, are found to consist of a homogeneous rubber core enclosed by a contiguous, monolayer biomembrane. Planta 210, 85–96. doi: 10.1007/s004250050657 10592036

[B25] Cox J. Mann M. (2008). MaxQuant enables high peptide identification rates, individualized p.p.b.-range mass accuracies and proteome-wide protein quantification. Nat. Biotechnol. 26, 1367–1372. doi: 10.1038/nbt.1511 19029910

[B26] Cozza G. Rossetto M. Bosello-Travain V. Maiorino M. Roveri A. Toppo S.. (2017). Glutathione peroxidase 4-catalyzed reduction of lipid hydroperoxides in membranes: The polar head of membrane phospholipids binds the enzyme and addresses the fatty acid hydroperoxide group toward the redox center. Free Radical Biol. Med. 112, 1–11. doi: 10.1016/J.FREERADBIOMED.2017.07.010 28709976

[B27] Crowell D. N. (2000). Functional implications of protein isoprenylation in plants. Prog. Lipid Res. 39, 393–408. doi: 10.1016/S0163-7827(00)00010-2 11082505

[B28] Curwin A. J. LeBlanc M. A. Fairn G. D. McMaster C. R. (2013). Localization of lipid raft proteins to the plasma membrane is a major function of the phospholipid transfer protein Sec14. PloS One 8, e55388. doi: 10.1371/JOURNAL.PONE.0055388 23383173 PMC3559501

[B29] Dai L. Kang G. Li Y. Nie Z. Duan C. Zeng R. (2013). In-depth proteome analysis of the rubber particle of Hevea brasiliensis (para rubber tree). Plant Mol. Biol. 82, 155–168. doi: 10.1007/s11103-013-0047-y 23553221

[B30] Deák M. Horváth G. V. Davletova S. Török K. Sass L. Vass I.. (1999). Plants ectopically expressing the ironbinding protein, ferritin, are tolerant to oxidative damage and pathogens. Nat. Biotechnol. 17, 192–196. doi: 10.1038/6198 10052358

[B31] Dellagi A. Rigault M. Segond D. Roux C. Kraepiel Y. Cellier F.. (2005). Siderophore-mediated upregulation of Arabidopsis ferritin expression in response to Erwinia chrysanthemi infection. Plant J. 43, 262–272. doi: 10.1016/S0021-9258(18)51510-6 15998312

[B32] Dennis M. S. Light D. R. (1989). Rubber elongation factor from Hevea brasiliensis. Identification, characterization, and role in rubber biosynthesis. J. Biol. Chem. 264, 18608–18617. doi: 10.1111/J.1365-313X.2005.02451.X 2681199

[B33] Dickenson P. B. (1969). Electron microscopial studies of latex vessel system of Hevea brasiliensis. J. Rubber Res. 21, 543–559.

[B34] Dietz K. J. (2003). Plant peroxiredoxins. Annu. Rev. Plant Biol. 54, 93–107. doi: 10.1146/ANNUREV.ARPLANT.54.031902.134934/CITE/REFWORKS 14502986

[B35] Dixit P. Mukherjee P. K. Ramachandran V. Eapen S. (2011). Glutathione Transferase from Trichoderma virens Enhances Cadmium Tolerance without Enhancing Its Accumulation in Transgenic Nicotiana tabacum. PloS One 6, e16360. doi: 10.1371/JOURNAL.PONE.0016360 21283689 PMC3024989

[B36] Dixon D. P. Edwards R. (2009). Selective binding of glutathione conjugates of fatty acid derivatives by plant glutathione transferases. J. Biol. Chem. 284, 21249–21256. doi: 10.1074/jbc.M109.020107 19520850 PMC2755848

[B37] Dixon D. P. Hawkins T. Hussey P. J. Edwards R. (2009). Enzyme activities and subcellular localization of members of the Arabidopsis glutathione transferase superfamily. J. Exp. Bot. 60, 1207–1218. doi: 10.1093/JXB/ERN365 19174456 PMC2657551

[B38] Dong G. Fan M. Wang H. Leng Y. Sun J. Huang J.. (2023a). Functional characterization of TkSRPP promoter in response to hormones and wounding stress in transgenic tobacco. Plants 12, 252. doi: 10.3390/PLANTS12020252 36678964 PMC9866153

[B39] Dong G. Wang H. Qi J. Leng Y. Huang J. Zhang H.. (2023b). Transcriptome analysis of Taraxacum kok-saghyz reveals the role of exogenous methyl jasmonate in regulating rubber biosynthesis and drought tolerance. Gene 867, 147346. doi: 10.1016/J.GENE.2023.147346 36898514

[B40] Epping J. van Deenen N. Niephaus E. Stolze A. Fricke J. Huber C.. (2015). A rubber transferase activator is necessary for natural rubber biosynthesis in dandelion. Nat. Plants 1. doi: 10.1038/nplants.2015.48

[B41] Eyal Y. Meller Y. Lev-Yadun S. Fluhr R. (1993). A basic-type PR-1 promoter directs ethylene responsiveness, vascular and abscission zone-specific expression. Plant J. 4, 225–234. doi: 10.1046/J.1365-313X.1993.04020225.X 8220480

[B42] Fernández-Santos R. Izquierdo Y. López A. Muñiz L. Martínez M. Cascón T.. (2020). Protein Profiles of Lipid Droplets during the Hypersensitive Defense Response of Arabidopsis against Pseudomonas Infection. Plant Cell Physiol. 61, 1144–1157. doi: 10.1093/PCP/PCAA041 32219438

[B43] Frey-Wyssling A. (1929). Microscopic investigations on the occurrence of resins in Hevea latex. Arch. Rubbercult 13, 392–294.

[B44] Fricke J. Hillebrand A. Twyman R. M. Prüfer D. Schulze Gronover C. (2013). Abscisic acid-dependent regulation of small rubber particle protein gene expression in Taraxacum brevicorniculatum is mediated by TbbZIP1. Plant Cell Physiol. 54, 448–464. doi: 10.1093/pcp/pcs182 23303876

[B45] Galvāo V. C. Fiorucci A. S. Trevisan M. Franco-Zorilla J. M. Goyal A. Schmid-Siegert E.. (2019). PIF transcription factors link a neighbor threat cue to accelerated reproduction in Arabidopsis. Nat. Commun. 10, 1–10. doi: 10.1038/s41467-019-11882-7 31488833 PMC6728355

[B46] Gao Q. Shang Y. Huang W. Wang C. (2013). Glycerol-3-phosphate acyltransferase contributes to triacylglycerol biosynthesis, lipid droplet formation, and host invasion in Metarhizium robertsii. Appl. Environ. Microbiol. 79, 7646–7653. doi: 10.1128/AEM.02905-13/SUPPL_FILE/ZAM999104940SO1.PDF 24077712 PMC3837804

[B47] García Mata C. Lamattina L. Cassia R. O. (2001). Involvement of iron and ferritin in the potato-Phytophthora infestans interaction. Eur. J. Plant Pathol. 107, 557–562. doi: 10.1023/A:1011228317709/METRICS

[B48] Ge H. Liu Z. Church G. M. Vidal M. (2001). Correlation between transcriptome and interactome mapping data from Saccharomyces cerevisiae. Nat. Genet. 29, 482–486. doi: 10.1038/ng776 11694880

[B49] Gidda S. K. Park S. Pyc M. Yurchenko O. Cai Y. Wu P.. (2016). Lipid droplet-associated proteins (LDAPs) are required for the dynamic regulation of neutral lipid compartmentation in plant cells. Plant Physiol. 170, 2052–2071. doi: 10.1104/pp.15.01977 26896396 PMC4825156

[B50] Gidda S. K. Shockey J. M. Rothstein S. J. Dyer J. M. Mullen R. T. (2009). Arabidopsis thaliana GPAT8 and GPAT9 are localized to the ER and possess distinct ER retrieval signals: Functional divergence of the dilysine ER retrieval motif in plant cells. Plant Physiol. Biochem. 47, 867–879. doi: 10.1016/J.PLAPHY.2009.05.008 19539490

[B51] Gidda S. K. Watt S. C. Collins-Silva J. Kilaru A. Arondel V. Yurchenko O.. (2013). Lipid droplet-associated proteins (LDAPs) are involved in the compartmentalization of lipophilic compounds in plant cells. Plant Signal. Behav. 8, e27141–1–e27141-4. doi: 10.4161/psb.27141 24305619 PMC4091607

[B52] Gomez J. B. Hamzah S. (1989). Frey-Wyssling complex in hevea latex - uniqueness of the organelle. J. Natural Prod. 4, 75–85. doi: 10.1007/978-3-319-59379-1%0

[B53] Gong Q. Yao S. Wang X. Li G. (2024). Fine-tuning phosphatidic acid production for optimal plant stress responses. Trends Biochem. Sci 49 (8), 663–666. doi: 10.1016/j.tibs.2024.05.008 38908926 PMC11316626

[B54] González-Coloma A. López-Balboa C. Santana O. Reina M. Fraga B. M. (2011). Triterpene-based plant defenses. Phytochem. Rev. 10, 245–260. doi: 10.1007/S11101-010-9187-8/TABLES/4

[B55] Griseti E. Bello A. A. Bieth E. Sabbagh B. Iacovoni J. S. Bigay J.. (2024). Molecular mechanisms of perilipin protein function in lipid droplet metabolism. FEBS Lett. 598, 1170–1198. doi: 10.1002/1873-3468.14792 38140813

[B56] Grosshans B. L. Ortiz D. Novick P. (2006). Rabs and their effectors: Achieving specificity in membrane traffic. Proc. Natl. Acad. Sci. United States America 103, 11821–11827. doi: 10.1073/PNAS.0601617103/SUPPL_FILE/01617FIG8.PDF PMC1567661 16882731

[B57] Guiltinan M. J. Marcotte W. R. Quatrano R. S. (1990). A plant leucine zipper protein that recognizes an abscisic acid response element. Sci. (New York N.Y.) 250, 267–271. doi: 10.1126/SCIENCE.2145628 2145628

[B58] Guo D. Li H. L. Tang X. Peng S. Q. (2014). Molecular and functional characterization of the HbSRPP promoter in response to hormones and abiotic stresses. Transgenic Res. 23, 331–340. doi: 10.1007/S11248-013-9753-0/FIGURES/4 24043397

[B59] Ha J. H. Loh S. N. (2012). Protein conformational switches: from nature to design. Chem. (Weinheim an Der Bergstrasse Germany) 18, 7984. doi: 10.1002/CHEM.201200348 PMC3404493 22688954

[B60] Halliwell B. Gutteridge J. M. C. (1984). Oxygen toxicity, oxygen radicals, transition metals and disease. Biochem. J. 219, 1. doi: 10.1042/BJ2190001 6326753 PMC1153442

[B61] Hampton R. Y. Koning A. Wright R. Rine J. (1996). *In vivo* examination of membrane protein localization and degradation with green fluorescent protein. Proc. Natl. Acad. Sci. United States America 93, 828. doi: 10.1073/PNAS.93.2.828 PMC40142 8570643

[B62] Hancock J. F. Cadwallader K. Marshall1 C. J. (1991). Methylation and proteolysis are essential for efficient membrane binding of prenylated p21K-ras(B). EMBO J. 10, 641–646. doi: 10.1002/J.1460-2075.1991.TB07992.X 2001678 PMC452695

[B63] Harayama T. Riezman H. (2018). Understanding the diversity of membrane lipid composition. Nat. Rev. Mol. Cell Biol. 19, 281–296. doi: 10.1038/nrm.2017.138 29410529

[B64] He H. Wang J. Meng Z. Dijkwel P. P. Du P. Shi S.. (2024). Genome-Wide Analysis of the SRPP/REF Gene Family in Taraxacum kok-saghyz Provides Insights into Its Expression Patterns in Response to Ethylene and Methyl Jasmonate Treatments. Int. J. Mol. Sci. 25, 6864. doi: 10.3390/IJMS25136864 38999970 PMC11241686

[B65] Heberle H. Meirelles V. G. da Silva F. R. Telles G. P. Minghim R. (2015). InteractiVenn: A web-based tool for the analysis of sets through Venn diagrams. BMC Bioinf. 16, 1–7. doi: 10.1186/s12859-015-0611-3 PMC4455604 25994840

[B66] Heim M. A. Jakoby M. Werber M. Martin C. Weisshaar B. Bailey P. C. (2003). The basic helix–loop–helix transcription factor family in plants: a genome-wide study of protein structure and functional diversity. Mol. Biol. Evol. 20, 735–747. doi: 10.1093/MOLBEV/MSG088 12679534

[B67] Herman E. M. (2008). Endoplasmic reticulum bodies: solving the insoluble. Curr. Opin. Plant Biol. 11, 672–679. doi: 10.1016/j.pbi.2008.08.004 18824401

[B68] Hillebrand A. Post J. J. Wurbs D. Wahler D. Lenders M. Krzyzanek V.. (2012). Down-regulation of small rubber particle protein expression affects integrity of rubber particles and rubber content in Taraxacum brevicorniculatum. PloS One 7, e41874. doi: 10.1371/journal.pone.0041874 22911861 PMC3402443

[B69] Hong J. C. Cheong Y. H. Nagao R. T. Bahk J. D. Key J. L. Cho M. J. (1995). Isolation of two soybean G-box binding factors which interact with a G-box sequence of an auxin-responsive gene. Plant J. 8, 199–211. doi: 10.1046/J.1365-313X.1995.08020199.X 7670504

[B70] Horling F. König J. Dietz K. J. (2002). Type II peroxiredoxin C, a member of the peroxiredoxin family of Arabidopsis thaliana: its expression and activity in comparison with other peroxiredoxins. Plant Physiol. Biochem. 40, 491–499. doi: 10.1016/S0981-9428(02)01396-7

[B71] Horling F. Lamkemeyer P. König J. Finkemeier I. Kandlbinder A. Baier M.. (2003). Divergent light-, ascorbate-, and oxidative stress-dependent regulation of expression of the peroxiredoxin gene family in Arabidopsis. Plant Physiol. 131, 317–325. doi: 10.1104/PP.010017 12529539 PMC166811

[B72] Horn P. J. James C. N. Gidda S. K. Kilaru A. Dyer J. M. Mullen R. T.. (2013). Identification of a new class of lipid droplet-associated proteins in plants. Plant Physiol. 162, 1926–1936. doi: 10.1104/pp.113.222455 23821652 PMC3729771

[B73] Huang A. H. C. (2018). Plant lipid droplets and their associated proteins: Potential for rapid advances. Plant Physiol. 176, 1894–1918. doi: 10.1104/pp.17.01677 29269574 PMC5841732

[B74] Huang M. Weissman J. T. Béraud-Dufour S. Luan P. Wang C. Chen W.. (2001). Crystal structure of Sar1-GDP at 1.7 A resolution and the role of the NH2 terminus in ER export. J. Cell Biol. 155, 937–948. doi: 10.1083/JCB.200106039 11739406 PMC2150902

[B75] Huber M. Epping J. Schulze Gronover C. Fricke J. Aziz Z. Brillatz T.. (2016). A latex metabolite benefits plant fitness under root herbivore attack. PloS Biol. 14, e1002332. doi: 10.1371/journal.pbio.1002332 26731567 PMC4701418

[B76] Huber M. Triebwasser-Freese D. Reichelt M. Heiling S. Paetz C. Chandran J. N.. (2015). Identification, quantification, spatiotemporal distribution and genetic variation of major latex secondary metabolites in the common dandelion (Taraxacum officinale agg.). Phytochemistry 115, 89–98. doi: 10.1016/J.PHYTOCHEM.2015.01.003 25682510

[B77] Hussain M. Debnath B. Qasim M. Bamisile B. S. Islam W. Hameed M. S.. (2019). Role of saponins in plant defense against specialist herbivores. Molecules 24, 2067. doi: 10.3390/molecules24112067 31151268 PMC6600540

[B78] Jach G. Pesch M. Richter K. Frings S. Uhrig J. F. (2006). An improved mRFP1 adds red to bimolecular fluorescence complementation. Nat. Methods 3, 597–600. doi: 10.1038/nmeth901 16862132

[B79] Jamsheer K M. Kumar M. Srivastava V. (2021). SNF1-related protein kinase 1: the many-faced signaling hub regulating developmental plasticity in plants. J. Exp. Bot. 72, 6042–6065. doi: 10.1093/JXB/ERAB079 33693699

[B80] Jansen R. Greenbaum D. Gerstein M. (2002). Relating whole-genome expression data with protein-protein interactions. Genome Res. 12, 37–46. doi: 10.1101/GR.205602 11779829 PMC155252

[B81] Javed T. Gao S. J. (2023). WRKY transcription factors in plant defense. Trends Genet. 39, 787–801. doi: 10.1016/J.TIG.2023.07.001 37633768

[B82] Jayawardhane K. N. Singer S. D. Weselake R. J. Chen G. (2018). Plant sn-glycerol-3-phosphate acyltransferases: biocatalysts involved in the biosynthesis of intracellular and extracellular lipids. Lipids 53, 469–480. doi: 10.1002/LIPD.12049 29989678

[B83] Jekat S. B. Ernst A. M. von Bohl A. Zielonka S. Twyman R. M. Noll G. A.. (2013). P-proteins in Arabidopsis are heteromeric structures involved in rapid sieve tube sealing. Front. Plant Sci. 4. doi: 10.3389/FPLS.2013.00225 PMC3700381 23840197

[B84] Johnsson N. Varshavsky A. (1994). Split ubiquitin as a sensor of protein interactions *in vivo* . Proc. Natl. Acad. Sci. U S A. 91, 10340–10344. doi: 10.1073/pnas.91.22.10340 7937952 PMC45015

[B85] Kasapoğlu A. G. Muslu S. Aygören A. S. Öner B. M. Güneş E. İlhan E.. (2024). Genome-wide characterization of the GPAT gene family in bean (Phaseolus vulgaris L.) and expression analysis under abiotic stress and melatonin. Genet. Resour. Crop Evol. 71, 4549–4569. doi: 10.1007/S10722-024-01899-3

[B86] Kato K. Horiba A. Hayashi H. Mizukami H. Terasaka K. (2022). Characterization of triterpene saponin glycyrrhizin transport by Glycyrrhiza glabra. Plants 11, 1250. doi: 10.3390/PLANTS11091250/S1 35567251 PMC9102456

[B87] Kawano Y. Kaneko-Kawano T. Shimamoto K. (2014). Rho family GTPase-dependent immunity in plants and animals. Front. Plant Sci. 5. doi: 10.3389/FPLS.2014.00522 PMC4196510 25352853

[B88] Kawasaki M. Nakayama K. Wakatsuki S. (2005). Membrane recruitment of effector proteins by Arf and Rab GTPases. Curr. Opin. Struct. Biol. 15, 681–689. doi: 10.1016/J.SBI.2005.10.015 16289847

[B89] Keilhauer E. C. Hein M. Y. Mann M. (2015). Accurate protein complex retrieval by affinity enrichment mass spectrometry (AE-MS) rather than affinity purification mass spectrometry (AP-MS). Mol. Cell. Proteomics 14, 120–135. doi: 10.1074/MCP.M114.041012 25363814 PMC4288248

[B90] Kesselmeier J. Urban B. (1983). Subcellular localization of saponins in green and etiolated leaves and green protoplasts of oat (Avena sativa L.). Protoplasma 114, 133–140. doi: 10.1007/BF01279877

[B91] Kim E. Y. Park K. Y. Seo Y. S. Kim W. T. (2016). Arabidopsis small rubber particle protein homolog SRPs play dual roles as positive factors for tissue growth and development and in drought stress responses. Plant Physiol. 170, 2494–2510. doi: 10.1104/pp.16.00165 26903535 PMC4825120

[B92] Kim E. Y. Seo Y. S. Lee H. Kim W. T. (2010). Constitutive expression of CaSRP1, a hot pepper small rubber particle protein homolog, resulted in fast growth and improved drought tolerance in transgenic Arabidopsis plants. Planta 232, 71–83. doi: 10.1007/s00425-010-1149-2 20361337

[B93] Kim I. J. Ryu S. B. Kwak Y. S. Kang H. (2004). A novel cDNA from Parthenium argentatum Gray enhances the rubber biosynthetic activity *in vitro**. J. Exp. Bot. 55, 377–385. doi: 10.1093/jxb/erh039 14718497

[B94] Kim I. S. Shin S. Y. Kim S. H. Yoon H. S. (2012). Ectopic expression of sweet potato MuS1 increases acquired stress tolerance and fermentation yield in Saccharomyces cerevisiae. J. Microbiol. 50, 544–546. doi: 10.1007/s12275-012-2043-3 22752921

[B95] Kim Y. N. Kim J. S. Seo S. G. Lee Y. Baek S. W. Kim I. S.. (2011). Cadmium resistance in tobacco plants expressing the MuSI gene. Plant Biotechnol. Rep. 5, 323–329. doi: 10.1007/S11816-011-0186-Z/FIGURES/3 22031812 PMC3191292

[B96] Kooijman E. E. Chupin V. de Kruijff B. Burger K. N. J. (2003). Modulation of membrane curvature by phosphatidic acid and lysophosphatidic acid. Traffic 4, 162–174. doi: 10.1034/J.1600-0854.2003.00086.X 12656989

[B97] Kretzschmar F. K. Doner N. M. Krawczyk H. E. Scholz P. Schmitt K. Valerius O.. (2020). Identification of low-abundance lipid droplet proteins in seeds and seedlings. Plant Physiol. 182, 1326. doi: 10.1104/PP.19.01255 31826923 PMC7054876

[B98] Kroh G. E. Pilon M. (2020). Regulation of iron homeostasis and use in chloroplasts. Int. J. Mol. Sci. 21, 3395. doi: 10.3390/IJMS21093395 32403383 PMC7247011

[B99] Kubo A. Arai Y. Nagashima S. Yoshikawa T. (2004). Alteration of sugar donor specificities of plant glycosyltransferases by a single point mutation. Arch. Biochem. Biophys. 429, 198–203. doi: 10.1016/J.ABB.2004.06.021 15313223

[B100] Kumar M. Kesawat M. S. Du X. Siddique K. H. M. Kant S. Chung S. M. (2024). In silico analysis and expression profiling reveal the presence of abiotic stress and developmental stage specific Aconitase genes in rice (Oryza sativa L.). Plant Stress 11, 100416. doi: 10.1016/J.STRESS.2024.100416

[B101] Kumar S. Trivedi P. K. (2018). Glutathione S-transferases: role in combating abiotic stresses including arsenic detoxification in plants. Front. Plant Sci. 9. doi: 10.3389/FPLS.2018.00751 PMC5999759 29930563

[B102] Kuroda Y. Suzuki N. Kataoka T. (1993). The effect of posttranslational modifications on the interaction of Ras2 with adenylyl cyclase. Science 259, 683–686. doi: 10.1126/SCIENCE.8430318 8430318

[B103] Labrou N. E. Papageorgiou A. C. Pavli O. Flemetakis E. (2015). Plant GSTome: structure and functional role in xenome network and plant stress response. Curr. Opin. Biotechnol. 32, 186–194. doi: 10.1016/J.COPBIO.2014.12.024 25614070

[B104] Laibach N. Hillebrand A. Twyman R. M. Prüfer D. Schulze Gronover C. (2015). Identification of a *Taraxacum brevicorniculatum* rubber elongation factor protein that is localized on rubber particles and promotes rubber biosynthesis. Plant J. 82, 609–620. doi: 10.1111/tpj.12836 25809497

[B105] Laibach N. Schmidl S. Müller B. Bergmann M. Prüfer D. Schulze Gronover C. (2018). Small rubber particle proteins from *Taraxacum brevicorniculatum* promote stress tolerance and influence the size and distribution of lipid droplets and artificial poly( *cis* -1,4-isoprene) bodies. Plant J. 93, 1045–1061. doi: 10.1111/tpj.13829 29377321

[B106] Lan P. Li W. Wang H. Ma W. (2010). Characterization, sub-cellular localization and expression profiling of the isoprenylcysteine methylesterase gene family in Arabidopsis thaliana. BMC Plant Biol. 10, 212–212. doi: 10.1186/1471-2229-10-212 20868530 PMC3017835

[B107] Lassowskat I. Hartl M. Hosp F. Boersema P. J. Mann M. Finkemeier I. (2017). Dimethyl-labeling-based quantification of the lysine acetylome and proteome of plants. Methods Mol. Biol. (Clifton N.J.) 1653, 65–81. doi: 10.1007/978-1-4939-7225-8_5 28822126

[B108] Lazar C. Burger T. (2022). *imputeLCMD: A Collection of Methods for Left-Censored Missing Data Imputation* (R package version 2.1). Available online at: https://cran.r-project.org/package=imputeLCMD.

[B109] Leivar P. González V. M. Castel S. Trelease R. N. López-Iglesias C. Arró M.. (2005). Subcellular localization of Arabidopsis 3-hydroxy-3-methylglutaryl-coenzyme A reductase. Plant Physiol. 137, 57–69. doi: 10.1104/PP.104.050245 15618432 PMC548838

[B110] Liebthal M. Maynard D. Dietz K. J. (2018). Peroxiredoxins and redox signaling in plants. Antioxid. Redox Signaling 28, 609–624. doi: 10.1089/ARS.2017.7164/ASSET/IMAGES/LARGE/FIGURE7.JPEG PMC5806080 28594234

[B111] Lin T. Xu X. Du H. Fan X. Chen Q. Hai C.. (2022). Extensive sequence divergence between the reference genomes of Taraxacum kok-saghyz and Taraxacum mongolicum. Sci. China Life Sci. 65, 515–528. doi: 10.1007/S11427-021-2033-2/METRICS 34939160

[B112] Lin T. Xu X. Ruan J. Liu S. Wu S. Shao X.. (2018). Genome analysis of Taraxacum kok-saghyz Rodin provides new insights into rubber biosynthesis. Natl. Sci. Rev. 5, 78–87. doi: 10.1093/nsr/nwx101

[B113] Liu D. Liu Y. Rao J. Wang G. Li H. Ge F.. (2013). Overexpression of the glutathione S-transferase gene from Pyrus pyrifolia fruit improves tolerance to abiotic stress in transgenic tobacco plants. Mol. Biol. 47, 515–523. doi: 10.1134/S0026893313040109 24466748

[B114] Liu M. Qiu W. He X. Zheng L. Song X. Han X.. (2016). Functional characterization of a gene in Sedum alfredii hance resembling rubber elongation factor endowed with functions associated with cadmium tolerance. Front. Plant Sci. 7. doi: 10.3389/fpls.2016.00965 PMC4925709 27446189

[B115] Liu Y. Ji X. Nie X. Qu M. Zheng L. Tan Z.. (2015). Arabidopsis AtbHLH112 regulates the expression of genes involved in abiotic stress tolerance by binding to their E-box and GCG-box motifs. New Phytol. 207, 692–709. doi: 10.1111/NPH.13387 25827016

[B116] Lo Cicero L. Catara V. Strano C. P. Bella P. Madesis P. Lo Piero A. R. (2017). Over-expression of CsGSTU promotes tolerance to the herbicide alachlor and resistance to Pseudomonas syringe pv. tabaci in transgenic tobacco. Biol. Plant. 61, 169–177. doi: 10.1007/S10535-016-0659-6/METRICS

[B117] Louveau T. Osbourn A. (2019). The sweet side of plant-specialized metabolism. Cold Spring Harbor Perspect. Biol. 11, a034744. doi: 10.1101/CSHPERSPECT.A034744 PMC6886449 31235546

[B118] Manevich Y. Feinstein S. I. Fisher A. B. (2004). Activation of the antioxidant enzyme 1-CYS peroxiredoxin requires glutathionylation mediated by heterodimerization with πGST. Proc. Natl. Acad. Sci. U S A. 101, 3780–3785. doi: 10.1073/PNAS.0400181101 15004285 PMC374321

[B119] Marrs K. A. (1996). The functions and regulation of glutathione s-transferases in plants. Annu. Rev. Plant Physiol. Plant Mol. Biol. 47, 127–158. doi: 10.1146/ANNUREV.ARPLANT.47.1.127 15012285

[B120] Mason H. S. DeWald D. B. Mullet J. E. (1993). Identification of a methyl jasmonate-responsive domain in the soybean vspB promoter. Plant Cell 5, 241–251. doi: 10.1105/TPC.5.3.241 8467221 PMC160266

[B121] Mazel A. Leshem Y. Tiwari B. S. Levine A. (2004). Induction of salt and osmotic stress tolerance by overexpression of an intracellular vesicle trafficking protein AtRab7 (AtRabG3e). Plant Physiol. 134, 118–128. doi: 10.1104/PP.103.025379 14657401 PMC316292

[B122] McAssey E. V. Gudger E. G. Zuellig M. P. Burke J. M. (2016). Population genetics of the rubber-producing Russian dandelion (Taraxacum kok-saghyz). PloS One 11, e0146417. doi: 10.1371/JOURNAL.PONE.0146417 26727474 PMC4703197

[B123] McCaskill D. Croteau R. (1998). Some caveats for bioengineering terpenoid metabolism in plants. Trends Biotechnol. 16, 349–355. doi: 10.1016/S0167-7799(98)01231-1

[B124] Menkens A. E. Schindler U. Cashmore A. R. (1995). The G-box: a ubiquitous regulatory DNA element in plants bound by the GBF family of bZIP proteins. Trends Biochem. Sci. 20, 506–510. doi: 10.1016/S0968-0004(00)89118-5 8571452

[B125] Milla P. Viola F. Oliaro-Bosso S. Rocco F. Cattel L. Joubert B. M.. (2003). Subcellular localization of oxidosqualene cyclases from Arabidopsis thaliana, Trypanosoma cruzi, and Pneumocystis carinii expressed in yeast. Lipids 37, 1171–1176. doi: 10.1007/S11745-002-1017-9/METRICS 12617471

[B126] Mishra M. K. Singh G. Tiwari S. Singh R. Kumari N. Misra P. (2015). Characterization of Arabidopsis sterol glycosyltransferase TTG15/UGT80B1 role during freeze and heat stress. Plant Signal. Behav. 10, e1075682. doi: 10.1080/15592324.2015.1075682 26382564 PMC4854349

[B127] Moeder W. Del Pozo O. Navarre D. A. Martin G. B. Klessig D. F. (2007). Aconitase plays a role in regulating resistance to oxidative stress and cell death in Arabidopsis and Nicotiana benthamiana. Plant Mol. Biol. 63, 273–287. doi: 10.1007/S11103-006-9087-X/TABLES/1 17013749

[B128] Mofidi S. S. H. Naghavi M. R. Sabokdast M. Jariani P. Zargar M. Cornish K. (2024). Effect of drought stress on natural rubber biosynthesis and quality in Taraxacum kok-saghyz roots. PloS One 19, e0295694. doi: 10.1371/JOURNAL.PONE.0295694 38252676 PMC10802950

[B129] Moir G. F. J. (1959). Ultracentrifugation and staining of hevea latex. Nature 184, 1626–1628. doi: 10.1038/1841626a0

[B130] Moncrieffe M. C. Fernandez M. J. Spiteller D. Matsumura H. Gay N. J. Luisi B. F.. (2012). Structure of the glycosyltransferase EryCIII in complex with its activating P450 homologue EryCII. J. Mol. Biol. 415, 92–101. doi: 10.1016/J.JMB.2011.10.036 22056329 PMC3391682

[B131] Montag K. Ivanov R. Bauer P. (2023). Role of SEC14-like phosphatidylinositol transfer proteins in membrane identity and dynamics. Front. Plant Sci. 14. doi: 10.3389/FPLS.2023.1181031 PMC10225987 37255567

[B132] Müller B. Noll G. A. Ernst A. M. Rüping B. Groscurth S. Twyman R. M.. (2010). Recombinant artificial forisomes provide ample quantities of smart biomaterials for use in technical devices. Appl. Microbiol. Biotechnol. 88, 689–698. doi: 10.1007/s00253-010-2771-4 20665019

[B133] Murphy D. J. (2011). The dynamic roles of intracellular lipid droplets: from archaea to mammals. Protoplasma 249, 541–585. doi: 10.1007/S00709-011-0329-7 22002710

[B134] Mylona P. Owatworakit A. Papadopoulou K. Jenner H. Qin B. Findlay K.. (2008). Sad3 and Sad4 are required for saponin biosynthesis and root development in oat. Plant Cell 20, 201–212. doi: 10.1105/TPC.107.056531 18203919 PMC2254932

[B135] Nagao R. T. Goekjian V. H. Hong J. C. Key J. L. (1993). Identification of protein-binding DNA sequences in an auxin-regulated gene of soybean. Plant Mol. Biol. 21, 1147–1162. doi: 10.1007/BF00023610 8490133

[B136] Nianiou-Obeidat I. Madesis P. Kissoudis C. Voulgari G. Chronopoulou E. Tsaftaris A.. (2017). Plant glutathione transferase-mediated stress tolerance: functions and biotechnological applications. Plant Cell Rep. 36, 791–805. doi: 10.1007/S00299-017-2139-7 28391528

[B137] Nielsen E. (2020). The small GTPase superfamily in plants: A conserved regulatory module with novel functions. Annu. Rev. Plant Biol. 71, 247–272. doi: 10.1146/ANNUREV-ARPLANT-112619-025827/1 32442390

[B138] Nielsen E. Cheung A. Y. Ueda T. (2008). The regulatory RAB and ARF GTPases for vesicular trafficking. Plant Physiol. 147, 1516–1526. doi: 10.1104/PP.108.121798 18678743 PMC2492611

[B139] Niephaus E. Müller B. van Deenen N. Lassowskat I. Bonin M. Finkemeier I.. (2019). Uncovering mechanisms of rubber biosynthesis in Taraxacum koksaghyz – role of cis-prenyltransferase-like 1 protein. Plant J. 100, 591–609. doi: 10.1111/tpj.14471 31342578

[B140] Niu M. Xiong Y. Yan H. Zhang X. Li Y. da Silva J. A. T.. (2021). Cloning and expression analysis of mevalonate kinase and phosphomevalonate kinase genes associated with the MVA pathway in Santalum album. Sci. Rep. 11, 1–13. doi: 10.1038/s41598-021-96511-4 34413433 PMC8376994

[B141] Nowicki M. Zhao Y. Boggess S. L. Fluess H. Payá-Milans M. Staton M. E.. (2019). Taraxacum kok-saghyz (rubber dandelion) genomic microsatellite loci reveal modest genetic diversity and cross-amplify broadly to related species. Sci. Rep. 9, 1–17. doi: 10.1038/s41598-019-38532-8 30760810 PMC6374447

[B142] Oh S. K. Kang H. Shin D. H. Yang J. Chow K.-S. Yeang H. Y.. (1999). Isolation, Characterization, and Functional Analysis of a Novel cDNA Clone Encoding a Small Rubber Particle Protein from Hevea brasiliensis. J. Biol. Chem. 274, 17132–17138. doi: 10.1074/JBC.274.24.17132 10358068

[B143] Ohkama-Ohtsu N. Sasaki-Sekimoto Y. Oikawa A. Jikumaru Y. Shinoda S. Inoue E.. (2011). 12-oxo-phytodienoic acid–glutathione conjugate is transported into the vacuole in Arabidopsis. Plant Cell Physiol. 52, 205–209. doi: 10.1093/PCP/PCQ181 21097476

[B144] Padilla-Gonzalez G. F. dos Santos F. A. Da Costa F. B. (2016). Sesquiterpene lactones: more than protective plant compounds with high toxicity. Crit. Rev. Plant Sci. 35, 18–37. doi: 10.1080/07352689.2016.1145956

[B145] Panagabko C. Morley S. Hernandez M. Cassolato P. Gordon H. Parsons R.. (2003). Ligand specificity in the CRAL-TRIO protein family. Biochemistry 42, 6467–6474. doi: 10.1021/BI034086V 12767229

[B146] Panara F. Lopez L. Daddiego L. Fantini E. Facella P. Perrotta G. (2018). Comparative transcriptomics between high and low rubber producing Taraxacum kok-saghyz R. plants. BMC Genomics 19, 1–14. doi: 10.1186/S12864-018-5287-4 30514210 PMC6280347

[B147] Pascual J. Rahikainen M. Angeleri M. Alegre S. Gossens R. Shapiguzov A.. (2021). ACONITASE 3 is part of theANAC017 transcription factor-dependent mitochondrial dysfunction response. Plant Physiol. 186, 1859. doi: 10.1093/PLPHYS/KIAB225 34618107 PMC8331168

[B148] Paysan-Lafosse T. Blum M. Chuguransky S. Grego T. Pinto B. L. Salazar G. A.. (2023). InterPro in 2022. Nucleic Acids Res. 51, D418–D427. doi: 10.1093/NAR/GKAC993 36350672 PMC9825450

[B149] Post J. van Deenen N. Fricke J. Kowalski N. Wurbs D. Schaller H.. (2012). Laticifer-specific cis-prenyltransferase silencing affects the rubber, triterpene, and inulin content of Taraxacum brevicorniculatum. Plant Physiol. 158, 1406–1417. doi: 10.1104/pp.111.187880 22238421 PMC3291264

[B150] Pu X. Dong X. Li Q. Chen Z. Liu L. (2021). An update on the function and regulation of methylerythritol phosphate and mevalonate pathways and their evolutionary dynamics. J. Integr. Plant Biol. 63, 1211–1226. doi: 10.1111/JIPB.13076/SUPPINFO 33538411

[B151] Puskas J. E. Gautriaud E. Deffieux A. Kennedy J. P. (2006). Natural rubber biosynthesis—A living carbocationic polymerization? Prog. Polymer Sci. 31, 533–548. doi: 10.1016/J.PROGPOLYMSCI.2006.05.002

[B152] Pütter K. M. (2017). Isoprenoid pathway engineering in the rubber-producing genus Taraxacum (Germany: Westphalian Wilhelms-University Münster).

[B153] Pütter K. M. van Deenen N. Müller B. Fuchs L. Vorwerk K. Unland K.. (2019). The enzymes OSC1 and CYP716A263 produce a high variety of triterpenoids in the latex of Taraxacum koksaghyz. Sci. Rep. 9, 1–13. doi: 10.1038/s41598-019-42381-w 30976052 PMC6459903

[B154] Ramilowski J. A. Sawai S. Seki H. Mochida K. Yoshida T. Sakurai T.. (2013). Glycyrrhiza uralensis transcriptome landscape and study of phytochemicals. Plant Cell Physiol. 54, 697–710. doi: 10.1093/pcp/pct057 23589666

[B155] Ravet K. Touraine B. Boucherez J. Briat J. F. Gaymard F. Cellier F. (2009). Ferritins control interaction between iron homeostasis and oxidative stress in Arabidopsis. Plant J. 57, 400–412. doi: 10.1111/J.1365-313X.2008.03698.X 18826427

[B156] Reichel C. Johnsson N. (2005). The split-ubiquitin sensor: measuring interactions and conformational alterations of proteins *in vivo* . Methods Enzymol. 399, 757–776. doi: 10.1016/S0076-6879(05)99050-2 16338394

[B157] Robertlee J. Kobayashi K. Suzuki M. Muranaka T. (2017). AKIN10, a representative Arabidopsis SNF1-related protein kinase 1 (SnRK1), phosphorylates and downregulates plant HMG-CoA reductase. FEBS Lett. 591, 1159–1166. doi: 10.1002/1873-3468.12618 28263378

[B158] Rodriguez-Furlan C. Borna R. Betz O. (2023). RAB7 GTPases as coordinators of plant endomembrane traffic. Front. Plant Sci. 14. doi: 10.3389/FPLS.2023.1240973 PMC10470000 37662169

[B159] Rogowska A. Szakiel A. (2020). The role of sterols in plant response to abiotic stress. Phytochem. Rev. 19, 1525–1538. doi: 10.1007/S11101-020-09708-2

[B160] Roxas V. P. Lodhi S. A. Garrett D. K. Mahan J. R. Allen R. D. (2000). Stress tolerance in transgenic tobacco seedlings that overexpress glutathione S-transferase/glutathione peroxidase. Plant Cell Physiol. 41, 1229–1234. doi: 10.1093/PCP/PCD051 11092907

[B161] Rüdiger H. Gabius H. J. (2002). Plant lectins: Occurrence, biochemistry, functions and applications. Glycoconjugate J. 18, 589–613. doi: 10.1023/A:1020687518999 12376725

[B162] Salehi M. Cornish K. Bahmankar M. Naghavi M. R. (2021). Natural rubber-producing sources, systems, and perspectives for breeding and biotechnology studies of Taraxacum kok-saghyz. Ind. Crops Prod. 170, 113667. doi: 10.1016/J.INDCROP.2021.113667

[B163] Sando T. Takeno S. Watanabe N. Okumoto H. Kuzuyama T. Yamashita A.. (2008). Cloning and characterization of the 2- C -methyl- D -erythritol 4-phosphate (MEP) pathway genes of a natural-rubber producing plant, *Hevea brasiliensis* . Biosci. Biotechnol. Biochem. 72, 2903–2917. doi: 10.1271/bbb.80387 18997428

[B164] Santos Mendoza M. Dubreucq B. Miquel M. Caboche M. Lepiniec L. (2005). LEAFY COTYLEDON 2 activation is sufficient to trigger the accumulation of oil and seed specific mRNAs in *Arabidopsis* leaves. FEBS Lett. 579, 4666–4670. doi: 10.1016/j.febslet.2005.07.037 16107256

[B165] Sapperstein S. Berkower C. Michaelis S. (1994). Nucleotide sequence of the yeast STE14 gene, which encodes farnesylcysteine carboxyl methyltransferase, and demonstration of its essential role in a-factor export. Mol. Cell. Biol. 14, 1438–1449. doi: 10.1128/MCB.14.2.1438 8289819 PMC358499

[B166] Schmidt T. Hillebrand A. Wurbs D. Wahler D. Lenders M. Schulze Gronover C.. (2010a). Molecular cloning and characterization of rubber biosynthetic genes from Taraxacum koksaghyz. Plant Mol. Biol. Rep. 28, 277–284. doi: 10.1007/s11105-009-0145-9

[B167] Schmidt T. Lenders M. Hillebrand A. van Deenen N. Munt O. Reichelt R.. (2010b). Characterization of rubber particles and rubber chain elongation in Taraxacum koksaghyz. BMC Biochem. 11. doi: 10.1186/1471-2091-11-11 PMC2836272 20170509

[B168] Seitz S. B. Weisheit W. Mittag M. (2010). Multiple roles and interaction factors of an E-Box element in Chlamydomonas reinhardtii. Plant Physiol. 152, 2243. doi: 10.1104/PP.109.149195 20154097 PMC2850036

[B169] Seo S. G. Kim J. S. Yang Y. S. Jun B. K. Kang S. W. Lee G. P.. (2010). Cloning and characterization of the new multiple stress responsible gene i (MuSI) from sweet potato. Genes Genomics 32, 544–552. doi: 10.1007/s13258-010-0093-7

[B170] Sevilla F. Camejo D. Ortiz-Espín A. Calderón A. Lázaro J. J. Jiménez A. (2015). The thioredoxin/peroxiredoxin/sulfiredoxin system: current overview on its redox function in plants and regulation by reactive oxygen and nitrogen species. J. Exp. Bot. 66, 2945–2955. doi: 10.1093/JXB/ERV146 25873657

[B171] Shahmuradov I. A. Solovyev V. V. (2015). Nsite, NsiteH and NsiteM computer tools for studying transcription regulatory elements. Bioinformatics 31, 3544. doi: 10.1093/BIOINFORMATICS/BTV404 26142184 PMC4612222

[B172] Shaikhali J. Norén L. De Dios Barajas-López J. Srivastava V. König J. Sauer U. H.. (2012). Redox-mediated mechanisms regulate DNA binding activity of the G-group of basic region leucine zipper (bZIP) transcription factors in Arabidopsis. J. Biol. Chem. 287, 27510–27525. doi: 10.1074/jbc.M112.361394 22718771 PMC3431687

[B173] Shockey J. Regmi A. Cotton K. Adhikari N. Browse J. Bates P. D. (2015). Identification of Arabidopsis GPAT9 (At5g60620) as an essential gene involved in triacylglycerol biosynthesis. Plant Physiol. 170, 163–179. doi: 10.1104/PP.15.01563 26586834 PMC4704598

[B174] Sibéril Y. Doireau P. Gantet P. (2001). Plant bZIP G-box binding factors. Eur. J. Biochem. 268, 5655–5666. doi: 10.1046/J.0014-2956.2001.02552.X 11722549

[B175] Sikorski R. S. Hieter P. (1989). A system of shuttle vectors and yeast host strains designed for efficient manipulation of DNA in Saccharomyces ceratisiae. Genetics 122, 19–27. Available at: https://www.ncbi.nlm.nih.gov/pmc/articles/PMC1203683/. 2659436 10.1093/genetics/122.1.19 PMC1203683

[B176] Simkin A. J. Guirimand G. Papon N. Courdavault V. Thabet I. Ginis O.. (2011). Peroxisomal localization of the final steps of the mevalonic acid pathway in planta. Planta 234, 903–914. doi: 10.1007/S00425-011-1444-6/FIGURES/5 21655959

[B177] Slocombe S. P. Cornah J. Pinfield-Wells H. Soady K. Zhang Q. Gilday A.. (2009). Oil accumulation in leaves directed by modification of fatty acid breakdown and lipid synthesis pathways. Plant Biotechnol. J. 7, 694–703. doi: 10.1111/J.1467-7652.2009.00435.X 19702756

[B178] Spang A. (2008). Membrane traffic in the secretory pathway: The life cycle of a transport vesicle. Cell. Mol. Life Sci. 65, 2781–2789. doi: 10.1007/S00018-008-8349-Y/METRICS 18726180 PMC11131685

[B179] Springer S. Spang A. Schekman R. (1999). A primer on vesicle budding. Cell 97, 145–148. doi: 10.1016/S0092-8674(00)80722-9 10219233

[B180] Srere P. A. (1972). “Is there an organization of Krebs cycle enzymes in the mitochondrial matrix?” In Energy metabolism and the regulation of metabolic processes in mitochondria. Mehlman M. A. Hanson R. W. (Eds.), (Academic Press) 79–91. doi: 10.1016/B978-0-12-487850-1.50011-7

[B181] Srere P. A. (1987). Complexes of sequential metabolic enzymes. Annu. Rev. Biochem. 56, 89–124. doi: 10.1146/ANNUREV.BI.56.070187.000513 2441660

[B182] Stucky D. F. Arpin J. C. Schrick K. (2014). Functional diversification of two UGT80 enzymes required for steryl glucoside synthesis in Arabidopsis. J. Exp. Bot. 66, 189–201. doi: 10.1093/JXB/ERU410 25316063 PMC4265157

[B183] Sugden C. Donaghy P. G. Halford N. G. Hardie D. G. (1999). Two SNF1-related protein kinases from spinach leaf phosphorylate and inactivate 3-hydroxy-3-methylglutaryl-coenzyme a reductase, nitrate reductase, and sucrose phosphate synthase *in vitro* . Plant Physiol. 120, 257–274. doi: 10.1104/PP.120.1.257 10318703 PMC59258

[B184] Sui N. Li M. Liu X. Y. Wang N. Fang W. Meng Q. W. (2007a). Response of xanthophyll cycle and chloroplastic antioxidant enzymes to chilling stress in tomato over-expressing glycerol-3-phosphate acyltransferase gene. Photosynthetica 45, 447–454. doi: 10.1007/S11099-007-0074-5

[B185] Sui N. Li M. Zhao S. J. Li F. Liang H. Meng Q. W. (2007b). Overexpression of glycerol-3-phosphate acyltransferase gene improves chilling tolerance in tomato. Planta 226, 1097–1108. doi: 10.1007/S00425-007-0554-7/FIGURES/6 17541789

[B186] Sun L. P. Ouyang L. L. Bao H. Liu J. G. Sun Z. Zhou Z. G. (2021). Comparison between two isoforms of glycerol-3-phosphate acyltransferase in microalga Myrmecia incisa: Subcellular localization and role in triacylglycerol synthesis. Algal Res. 54, 102172. doi: 10.1016/J.ALGAL.2020.102172

[B187] Tamura K. Stecher G. Kumar S. (2021). MEGA11: molecular evolutionary genetics analysis version 11. Mol. Biol. Evol. 38, 3022–3027. doi: 10.1093/MOLBEV/MSAB120 33892491 PMC8233496

[B188] Tanaka Y. Aik-Hwee E. Ohya N. Nishiyama N. Tangpakdee J. Kawahara S.. (1996). Initiation of rubber biosynthesis in Hevea brasiliensis: characterization of initiating species by structural analysis. Phytochemistry 41, 1501–1505. doi: 10.1016/0031-9422(95)00817-9

[B189] Team R. C. (2021). R: A language and environment for statistical computing (RStudio 2022.12.0). R Foundation for Statistical Computing. Available at: https://www.r-project.org/ Under the given URL one can download R-.

[B190] Testerink C. Munnik T. (2011). Molecular, cellular, and physiological responses to phosphatidic acid formation in plants. J. Exp. Bot. 62, 2349–2361. doi: 10.1093/JXB/ERR079 21430291

[B191] Thomas P. D. Ebert D. Muruganujan A. Mushayahama T. Albou L. P. Mi H. (2022). PANTHER: Making genome-scale phylogenetics accessible to all. Protein Sci. 31, 8–22. doi: 10.1002/pro.4218 34717010 PMC8740835

[B192] Tiwari V. Patel M. K. Chaturvedi A. K. Mishra A. Jha B. (2016). Functional Characterization of the Tau Class Glutathione-S-Transferases Gene (SbGSTU) Promoter of Salicornia brachiata under Salinity and Osmotic Stress. PloS One 11, e0148494. doi: 10.1371/JOURNAL.PONE.0148494 26885663 PMC4757536

[B193] Toledo-Ortiz G. Johansson H. Lee K. P. Bou-Torrent J. Stewart K. Steel G.. (2014). The HY5-PIF regulatory module coordinates light and temperature control of photosynthetic gene transcription. PloS Genet. 10, e1004416. doi: 10.1371/JOURNAL.PGEN.1004416 24922306 PMC4055456

[B194] Tripathy M. K. Tiwari B. S. Reddy M. K. Deswal R. Sopory S. K. (2017). Ectopic expression of PgRab7 in rice plants (Oryza sativa L.) results in differential tolerance at the vegetative and seed setting stage during salinity and drought stress. Protoplasma 254, 109–124. doi: 10.1007/S00709-015-0914-2/FIGURES/11 26666551

[B195] Tyanova S. Temu T. Cox J. (2016). The MaxQuant computational platform for mass spectrometry-based shotgun proteomics. Nat. Protoc. 11, 2301–2319. doi: 10.1038/nprot.2016.136 27809316

[B196] Unland K. Pütter K. M. Vorwerk K. van Deenen N. Twyman R. M. Prüfer D.. (2018). Functional characterization of squalene synthase and squalene epoxidase in Taraxacum koksaghyz. Plant Direct 2, 1–15. doi: 10.1002/pld3.63 PMC6508512 31245726

[B197] Urban B. Laudenbach U. Kesselmeier J. (1983). Saponin distribution in the etiolated leaf tissue and subcellular localization of steroidal saponins in etiolated protoplasts of oat (Avena sativa L.). Protoplasma 118, 121–123. doi: 10.1007/BF01293068/METRICS

[B198] Van Deenen N. Bachmann A. L. Schmidt T. Schaller H. Sand J. Prü fer D.. (2012). Molecular cloning of mevalonate pathway genes from Taraxacum brevicorniculatum and functional characterization of the key enzyme 3-hydroxy-3-methylglutaryl-coenzyme A reductase. Mol. Biol. Rep. 39, 4337–4339. doi: 10.1007/S11033-011-1221-4/TABLES/3 21833516

[B199] Vogt T. Jones P. (2000). Glycosyltransferases in plant-natural product synthesis: Characterization of a supergene family. Trends Plant Sci. 5, 380–386. doi: 10.1016/S1360-1385(00)01720-9 10973093

[B200] Volkman B. F. Lipson D. Wemmer D. E. Kern D. (2001). Two-state allosteric behavior in a single-domain signaling protein. Science 291, 2429–2433. doi: 10.1126/SCIENCE.291.5512.2429 11264542

[B201] von Mering C. Krause R. Snel B. Cornell M. Oliver S. G. Fields S.. (2002). Comparative assessment of large-scale data sets of protein–protein interactions. Nature 417, 399–403. doi: 10.1038/nature750 12000970

[B202] Voorrips R. E. (2002). MapChart: software for the graphical presentation of linkage maps and QTLs. J. Hered. 93, 77–78. doi: 10.1093/JHERED/93.1.77 12011185

[B203] Vranová E. Coman D. Gruissem W. (2012). Structure and dynamics of the isoprenoid pathway network. Mol. Plant 5, 318–333. doi: 10.1093/mp/sss015 22442388

[B204] Wagner U. Edwards R. Dixon D. P. Mauch F. (2002). Probing the diversity of the Arabidopsis glutathione S-transferase gene family. Plant Mol. Biol. 49, 515–532. doi: 10.1023/A:1015557300450/METRICS 12090627

[B205] Wahler D. Colby T. Kowalski N. A. Harzen A. Wotzka S. Y. Hillebrand A.. (2012). Proteomic analysis of latex from the rubber-producing plant Taraxacum brevicorniculatum. Proteomics 12, 901–905. doi: 10.1002/pmic.201000778 22539439

[B206] Wang J. Song J. Qi H. Zhang H. Wang L. Zhang H.. (2023). Overexpression of 2-Cys Peroxiredoxin alleviates the NaHCO3 stress-induced photoinhibition and reactive oxygen species damage of tobacco. Plant Physiol. Biochem. 201, 107876. doi: 10.1016/J.PLAPHY.2023.107876 37413942

[B207] Wani S. H. Anand S. Singh B. Bohra A. Joshi R. (2021). WRKY transcription factors and plant defense responses: latest discoveries and future prospects. Plant Cell Rep. 40, 1071–1085. doi: 10.1007/S00299-021-02691-8 33860345

[B208] Wei R. Wang J. Su M. Jia E. Chen S. Chen T.. (2018). Missing value imputation approach for mass spectrometry-based metabolomics data. Sci. Rep. 8, 663. doi: 10.1038/S41598-017-19120-0 29330539 PMC5766532

[B209] Wickham H. (2016). ggplot2: Elegant Graphics for Data Analysis (Springer-Verlag).

[B210] Wieghaus A. Roelfs K. U. Twyman R. M. Prüfer D. Schulze Gronover C. (2022). Comparative transcriptome analysis in Taraxacum koksaghyz to identify genes that determine root volume and root length. Front. Genet. 12. doi: 10.3389/FGENE.2021.784883/FULL PMC8819189 35140739

[B211] Wilfling F. Haas J. T. Walther T. C. Farese R. V. Jr. (2014). Lipid droplet biogenesis. Curr. Opin. Cell Biol. 29, 39–45. doi: 10.1016/j.ceb.2014.03.008 24736091 PMC4526149

[B212] Wilfling F. Wang H. Haas J. T. Krahmer N. Gould T. J. Uchida A.. (2013). Triacylglycerol synthesis enzymes mediate lipid droplet growth by relocalizing from the ER to lipid droplets. Dev. Cell 24, 384–399. doi: 10.1016/j.devcel.2013.01.013 23415954 PMC3727400

[B213] Williams M. E. Foster R. Chua N. H. (1992). Sequences flanking the hexametric G-box core CACGTG affect the specificity of protein binding. Plant Cell 4, 485–496. doi: 10.1105/TPC.4.4.485 1498606 PMC160147

[B214] Wititsuwannakul R. Rukseree K. Kanokwiroon K. Wititsuwannakul D. (2008). A rubber particle protein specific for Hevea latex lectin binding involved in latex coagulation. Phytochemistry 69, 1111–1118. doi: 10.1016/J.PHYTOCHEM.2007.12.007 18226821

[B215] Wood D. F. Cornish K. (2000). Microstructure of purified rubber particles. Int. J. Plant Sci. 161, 435–445. doi: 10.1086/314269 10817979

[B216] Wu Y. Dong G. Luo F. Xie H. Li X. Yan J. (2024). TkJAZs-TkMYC2-TkSRPP/REF regulates the biosynthesis of natural rubber in Taraxacum kok-saghyz. Plants 13, 2034. doi: 10.3390/plants13152034 39124151 PMC11314035

[B217] Xiao G. Zhao M. Liu Q. Zhou J. Cheng Z. Wang Q.. (2023). TaBAS1 encoding a typical 2-Cys peroxiredoxin enhances salt tolerance in wheat. Front. Plant Sci. 14. doi: 10.3389/FPLS.2023.1152375/BIBTEX PMC10043318 36998677

[B218] Xie W. McMahan C. M. DeGraw A. J. Distefano M. D. Cornish K. Whalen M. C.. (2008). Initiation of rubber biosynthesis: *In vitro* comparisons of benzophenone-modified diphosphate analogues in three rubber-producing species. Phytochemistry 69, 2539–2545. doi: 10.1016/J.PHYTOCHEM.2008.07.011 18799172

[B219] Xu T. Jia Z. Wu L. Chen Y. Luo Y. Jia D.. (2017). Effect of acetone extract from natural rubber on the structure and interface interaction in NR/CB composites. RSC Adv. 7, 26458–26467. doi: 10.1039/C7RA03354K

[B220] Yamashita S. Yamaguchi H. Waki T. Aoki Y. Mizuno M. Yanbe F.. (2016). Identification and reconstitution of the rubber biosynthetic machinery on rubber particles from Hevea brasiliensis. ELife 5, e19022. doi: 10.7554/eLife.19022 27790974 PMC5110245

[B221] Yang Q. Grimmig B. Matern U. (1998). Anthranilate N-hydroxycinnamoyl/benzoyltransferase gene from carnation: Rapid elicitation of transcription and promoter analysis. Plant Mol. Biol. 38, 1201–1214. doi: 10.1023/A:1006003731919/METRICS 9869425

[B222] Yang W. Pollard M. Li-Beisson Y. Beisson F. Feig M. Ohlrogge J. (2010). A distinct type of glycerol-3-phosphate acyltransferase with sn-2 preference and phosphatase activity producing 2-monoacylglycerol. Proc. Natl. Acad. Sci. United States America 107, 12040–12045. doi: 10.1073/PNAS.0914149107/SUPPL_FILE/SAPP.PDF PMC2900678 20551224

[B223] Zang X. Geng X. Wang F. Liu Z. Zhang L. Zhao Y.. (2017). Overexpression of wheat ferritin gene TaFER-5B enhances tolerance to heat stress and other abiotic stresses associated with the ROS scavenging. BMC Plant Biol. 17, 1–13. doi: 10.1186/S12870-016-0958-2/FIGURES/9 28088182 PMC5237568

[B224] Zhai Z. Liu H. Shanklin J. (2017). Phosphorylation of WRINKLED1 by KIN10 results in its proteasomal degradation, providing a link between energy homeostasis and lipid biosynthesis. Plant Cell 29, 871. doi: 10.1105/TPC.17.00019 28314829 PMC5435435

[B225] Zhang F. L. Casey P. J. (1996). Protein prenylation: Molecular mechanisms and functional consequences. Annu. Rev. Biochem. 65, 241–269. doi: 10.1146/ANNUREV.BI.65.070196.001325/CITE/REFWORKS 8811180

[B226] Zhang N. McHale L. K. Finer J. J. (2019). Changes to the core and flanking sequences of G-box elements lead to increases and decreases in gene expression in both native and synthetic soybean promoters. Plant Biotechnol. J. 17, 724. doi: 10.1111/PBI.13010 30191675 PMC6419578

[B227] Zhang Y. Fernie A. R. (2021). Metabolons, enzyme–enzyme assemblies that mediate substrate channeling, and their roles in plant metabolism. Plant Commun. 2, 100081. doi: 10.1016/J.XPLC.2020.100081 33511342 PMC7816073

[B228] Zhou Q. Yang Y. Yang Z. (2019). Molecular dissection of cadmium-responsive transcriptome profile in a low-cadmium-accumulating cultivar of Brassica parachinensis. Ecotoxicol. Environ. Saf. 176, 85–94. doi: 10.1016/J.ECOENV.2019.03.077 30921700

