# Supplementary material for: The interaction networks of small rubber particle proteins in the latex of Taraxacum koksaghyz reveal diverse functions in stress responses and secondary metabolism
Source: Front Plant Sci. 2024 Dec 13;15:1498737. doi: 10.3389/fpls.2024.1498737 (PMC11671276; doi:10.3389/fpls.2024.1498737)
Supplement: Supplementary file 1 [file DataSheet1.zip › Suppl Methods, Figures, Tables S1-5, 7.DOCX]

Supplementary Material

# Supplementary Data

## Supplementary Methods

### Supplementary Methods S1. Latex phase separation

Protease inhibitor (cOmplete Protease Inhibitor Cocktail; Roche, Switzerland) was dissolved in 2 mL double-distilled water and diluted 1:10 in ice-cold REB. Latex was collected from 11 plants in six 1.5-mL reaction tubes containing 800 µL REB and kept on ice. After centrifugation (5000 *g*, 5 min, 4 °C), latex was re-suspended and separated from the pellet. For the whole latex sample, 200 µL of latex was transferred to a fresh 1.5-mL reaction tube and diluted with 200 µL REB. Residual latex was mixed with 200 µL REB and separated into phases by centrifugation (14,000 *g*, 10 min, 4 °C). The interphase (IP) was removed with a syringe and pooled in a fresh tube. The remaining rubber phase (RP) was separated from the pellet phase (PP), pooled in a fresh tube and resuspended in 400 µL REB. The PP was resuspended and pooled in 600 µL REB. To remove contaminations of other phases, samples were centrifuged again and unwanted phases were removed. Further centrifugation steps were performed to abolish phase contaminations. The IP and PP yield was low, so IP and PP obtained during RP sample purification were added to the initial IP and PP samples. Finally, RP and PP were suspended in 400 µL REB.

### Supplementary Methods S2. LC-MS/MS-based quantitative proteomics

Peptides were separated on 17-cm fritless silica emitters (0.75 µm inner diameter; New Objective, USA), packed in-house with reversed-phase ReproSil-Pur C_18_ AQ 1.9 µm resin (Dr. Maisch, Germany). The column was constantly kept at 50 °C. Peptides were eluted in 115 min by applying a segmented linear gradient of 100% to 2% solvent A (0.1% formic acid in water) and 0% to 98% solvent B (0.1% formic acid in 80% acetonitrile in water) at a flowrate of 300 nL/min. Mass spectra were acquired in data-dependent acquisition mode according to a TOP15 method and collected in the Orbitrap analyzer over the *m/z* range 300–1759 at a resolution of 60,000 full width at half maximum (FWHM). The maximum injection time was 55 ms with a target value of 3×10^6^ ions. Precursors were selected with an isolation window of 1.3 *m/z*, and heated capillary dissociation fragmentation was applied at a normalized collision energy of 25 V. MS/MS spectra were acquired with a target value of 10^5^ ions at a resolution of 15,000 FWHM, maximum injection time of 55 ms and a fixed first mass of *m/z* = 100. Peptides with a charge of +1, > 6, or with unassigned charge state were excluded from fragmentation for MS^2^. Dynamic exclusion for 30 s prevented repeated selection of precursors.

### Supplementary Methods S3. RNA extraction, cDNA synthesis and quantitative PCR

RNA extraction, cDNA synthesis and qPCR were carried out as previously described (Niephaus *et al.*, 2019) with slight modifications. REB for latex harvesting contained 20 mM DTT. RNA extraction included the optional on-column DNA digest. For cDNA synthesis, 500 ng RNA or the maximum amount of 8 µL was used, if RNA concentrations were low. To assess RNA purity and cDNA quantity, cDNA was used as a template for control PCRs targeting the *TkGAPDH* gene with intron-spanning primers (Table S2), followed by agarose gel electrophoresis. The qPCR protocol was as previously described (Laibach *et al.*, 2015) with modifications. For spatial expression analysis, four or five individual plants were analyzed in three technical replicates for each tissue using KAPA SYBR FAST (Merck, Germany). The intron-spanning primers were tested for off-target binding by BLAST searches against the *T. koksaghyz* genomes (Lin *et al.*, 2018, 2022). Primer sequences and efficiencies are listed in Table S2. Two reference genes already shown to be suitable for *T. koksaghyz* were used for expression normalization: *elongation factor-1α* (*TkEF1α*) and *ribosomal protein L27* (*TkRP*) (Niephaus *et al.*, 2019). We applied a two-step cycling program using CFX96 and CFX Opus 96 real-time PCR systems (Bio-Rad Laboratories, USA), with a denaturation temperature of 95 °C and annealing/elongation temperature of 66 °C followed by melt curve profiling (58–95 °C). C_q_ values were determined using Bio-Rad CFX Maestro 2.3 software and single thresholds. Prior to qPCR experiments, primer efficiencies were determined using serial dilutions of a cDNA mixture obtained from different *T. koksaghyz* tissues. All primers were 90–110% efficient and the specificity of amplification was assessed by monitoring melting temperatures, amplicon sequences and the performance of no-target controls.

# Supplementary Figures and Tables

## Supplementary Figures


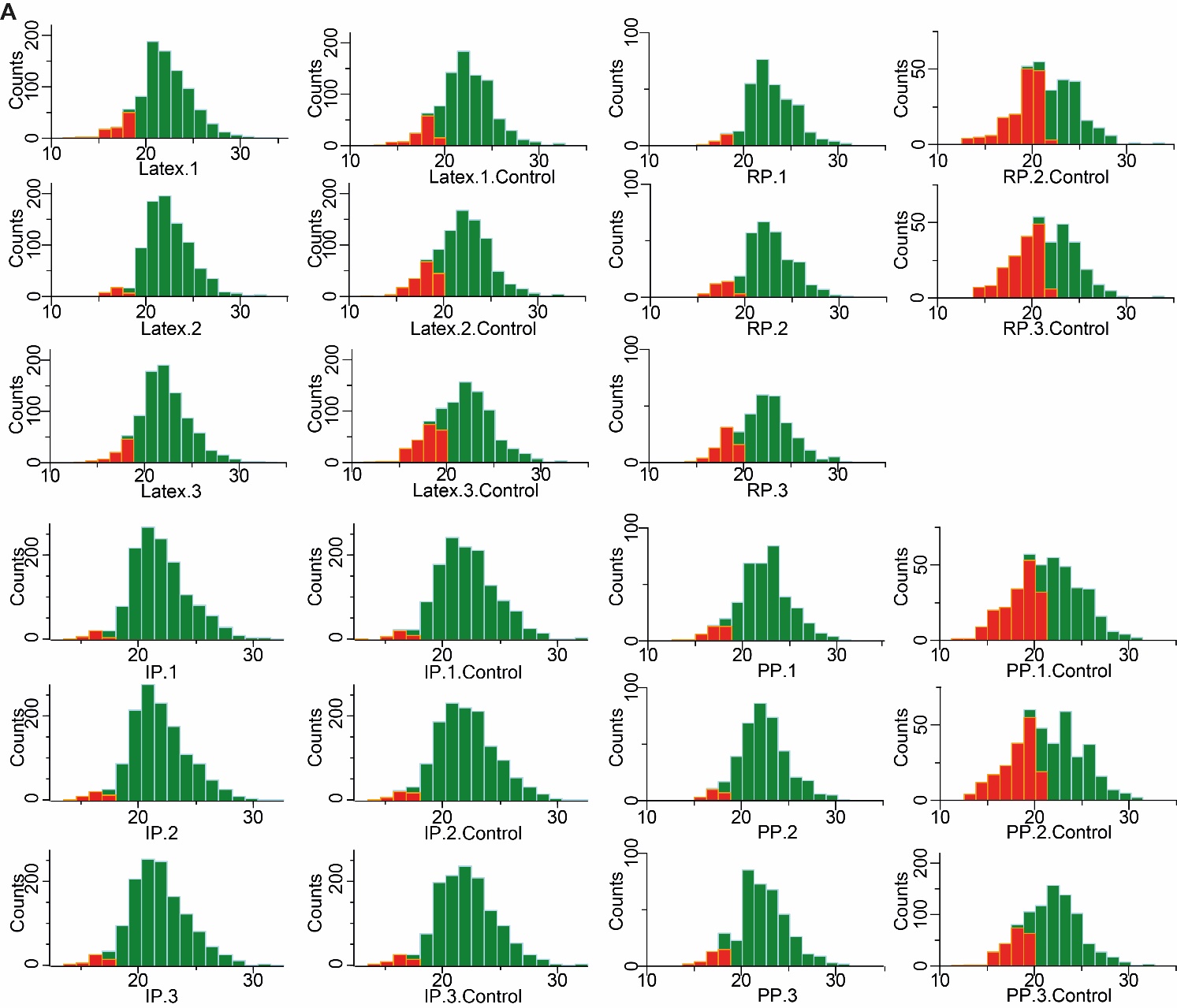

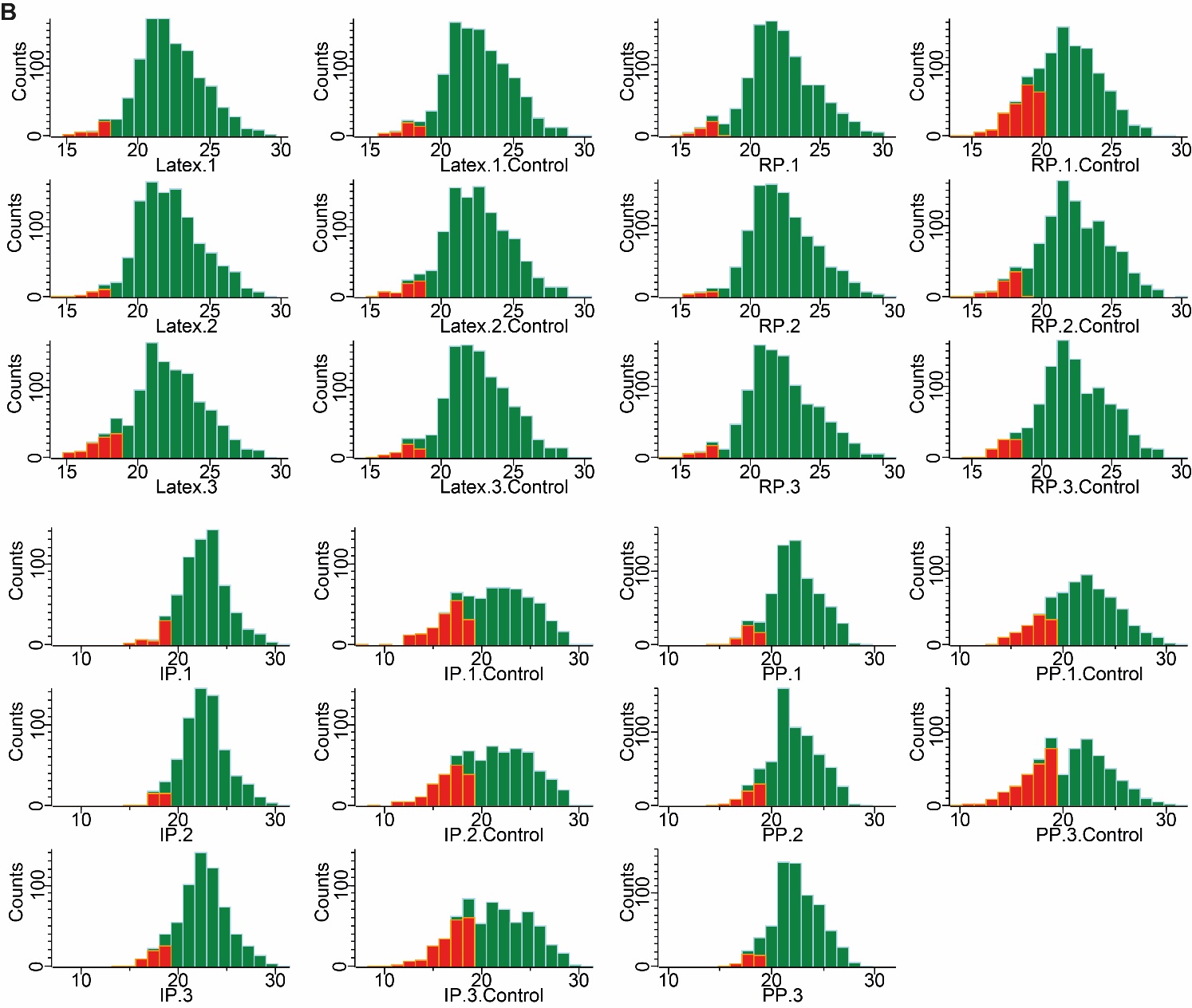

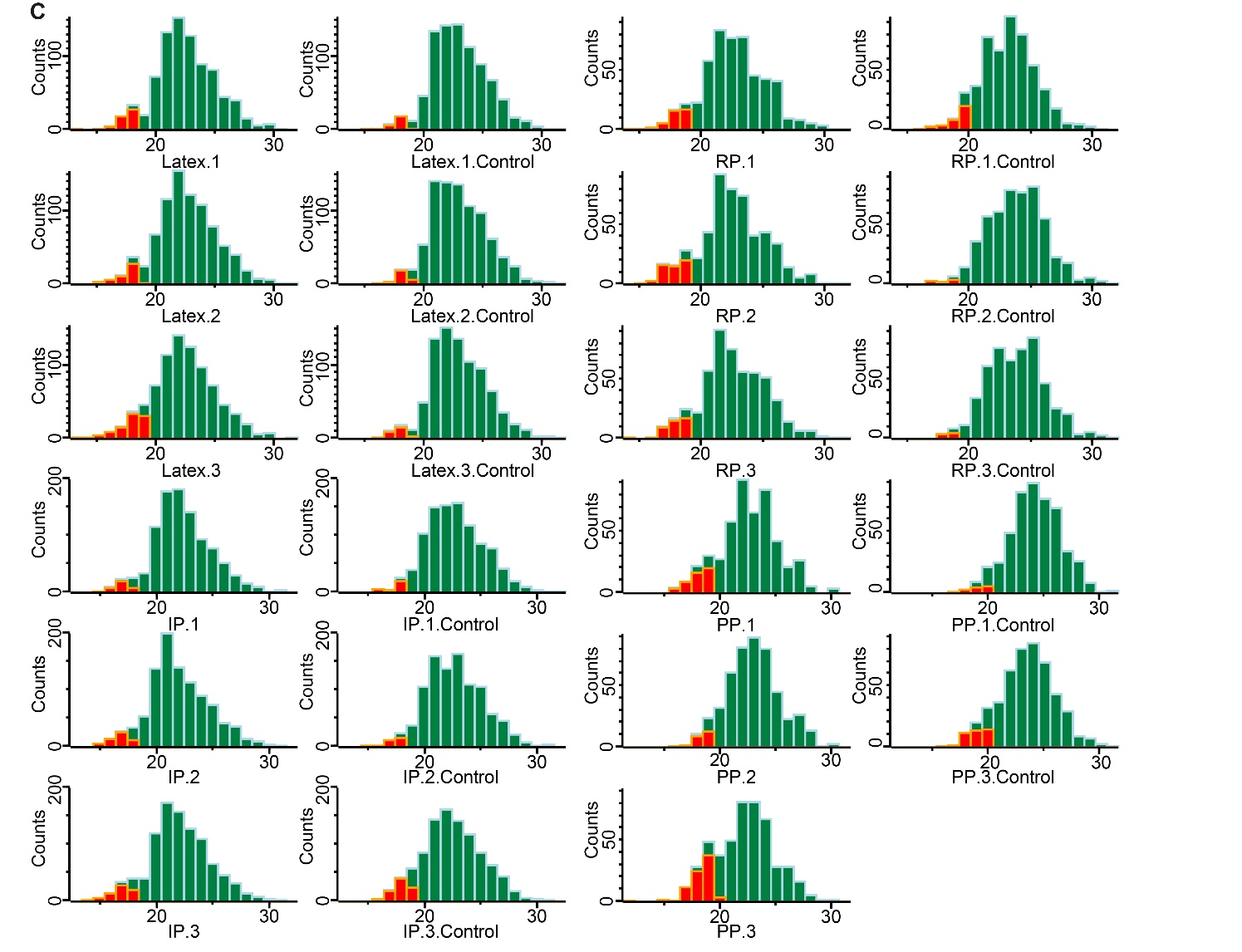


Supplementary Figure S1. Histograms of AE-MS samples. LFQ values are normally distributed across TkSRPP3 (A), TkSRPP4 (B) and TkSRPP5 (C) samples. Red bins represent values obtained by imputation.
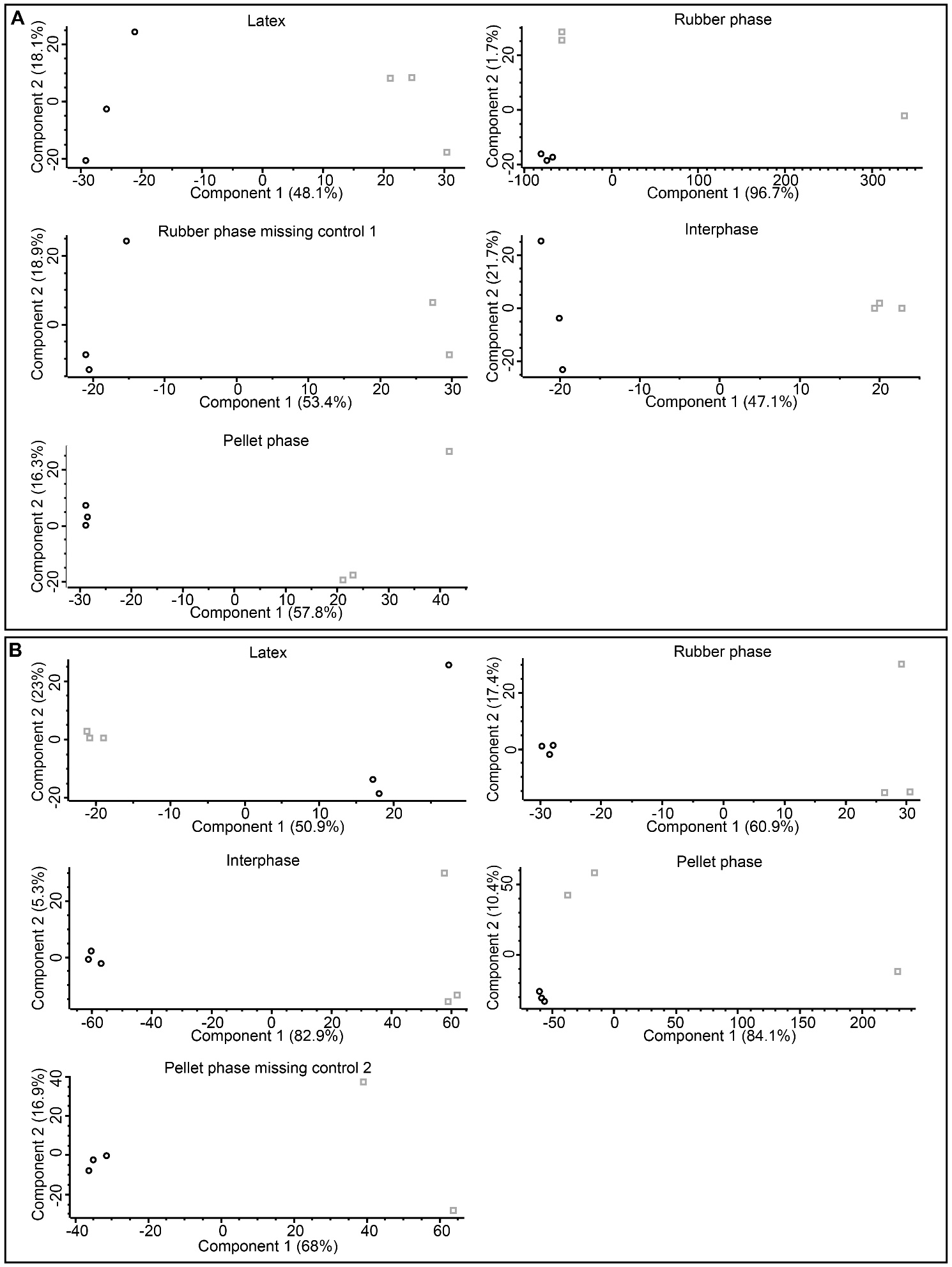

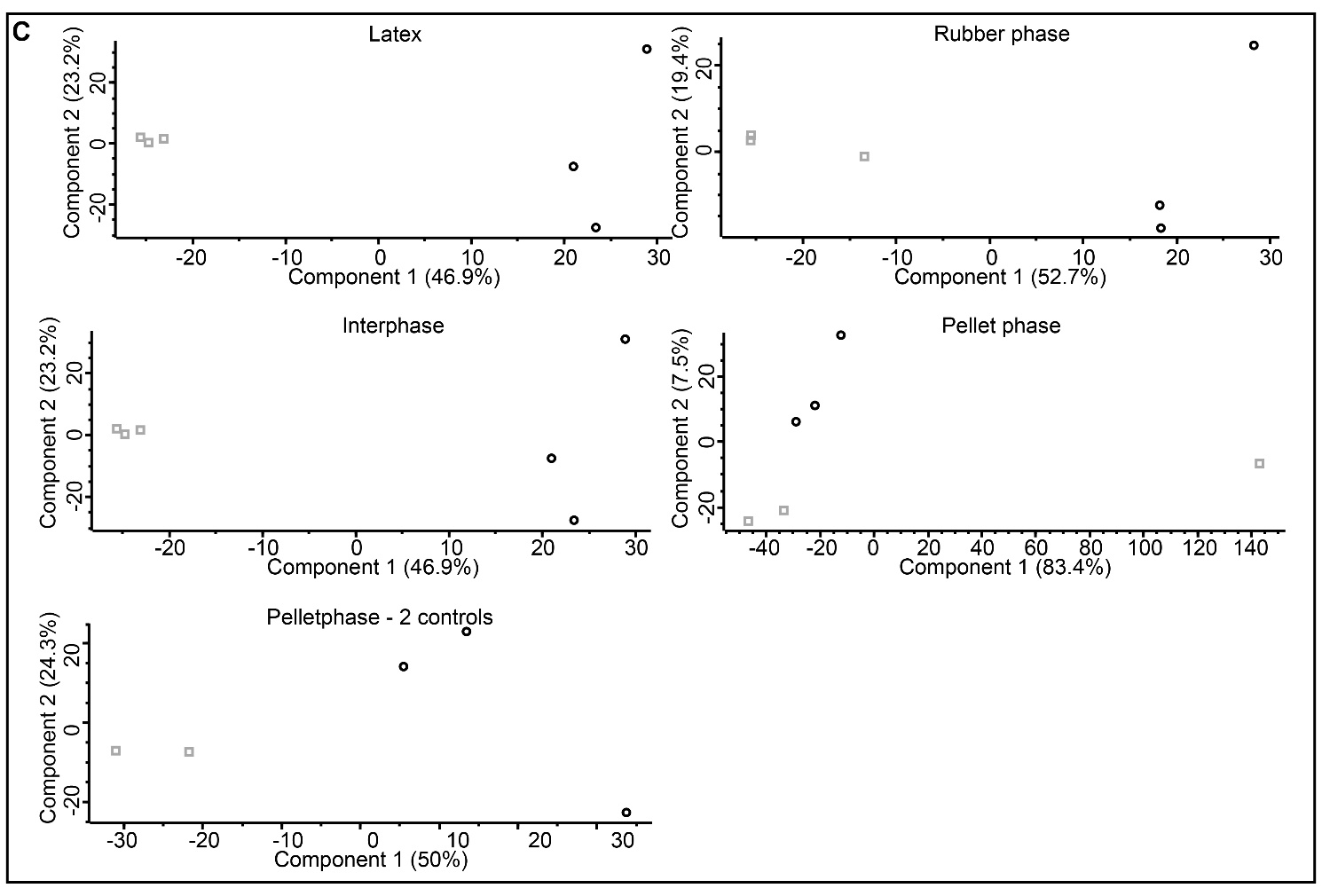


Supplementary Figure S2. Principal component analysis (PCA) of AE-MS samples. PCA plots of the TkSRPP3 (A), TkSRPP4 (B) and TkSRPP5 (C) AE-MS samples from the four different latex fractions and the corresponding no-bait controls. Black circles represent the three independent AE-MS replicates and gray boxes the three independent no-bait replicates. RP control 1 in the TkSRPP3 run and PP control 2 in the TkSRPP4/5 run separated from the other respective replicates and were therefore excluded from further analysis. PCA plots with and without these samples are shown.


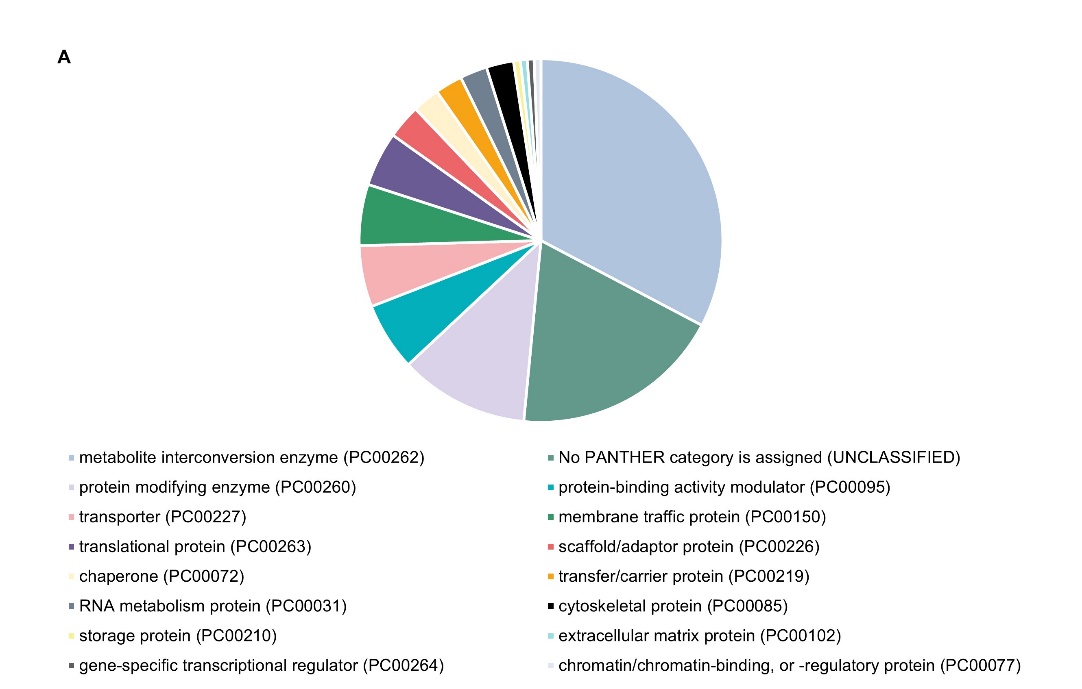

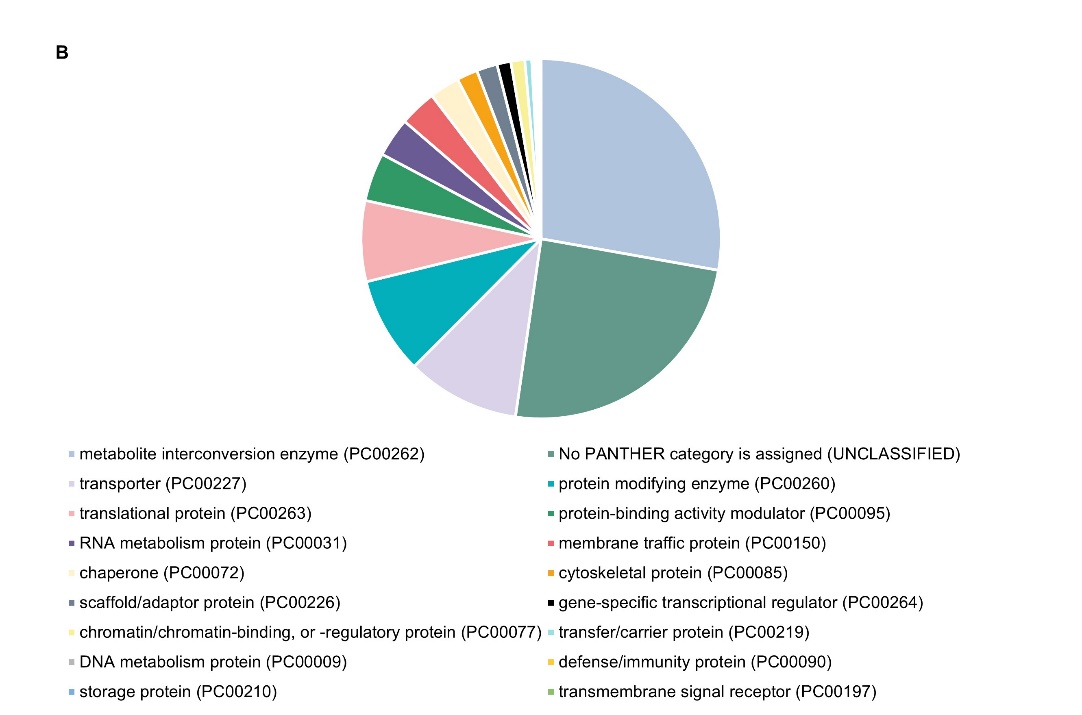

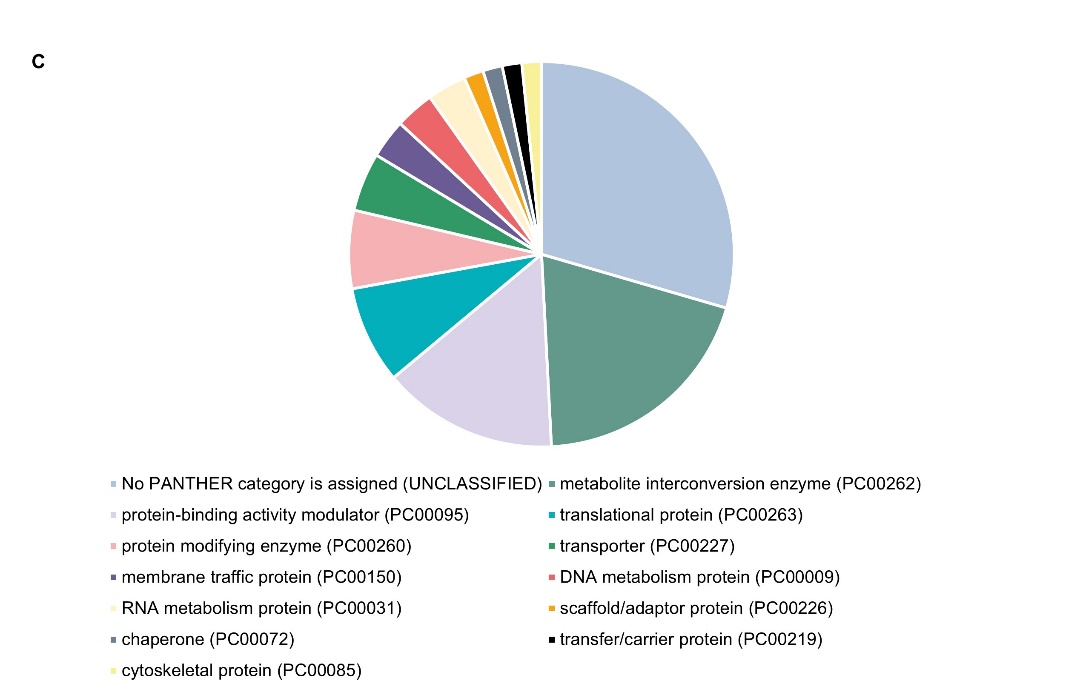


Supplementary Figure S3. Protein classes among TkSRPP interactors. Distribution of protein classes among all interactors of TkSRPP3 (A), TkSRPP4 (B) and TkSRPP5 (C) based on annotated UniProt IDs. Protein classes were determined using PANTHER.


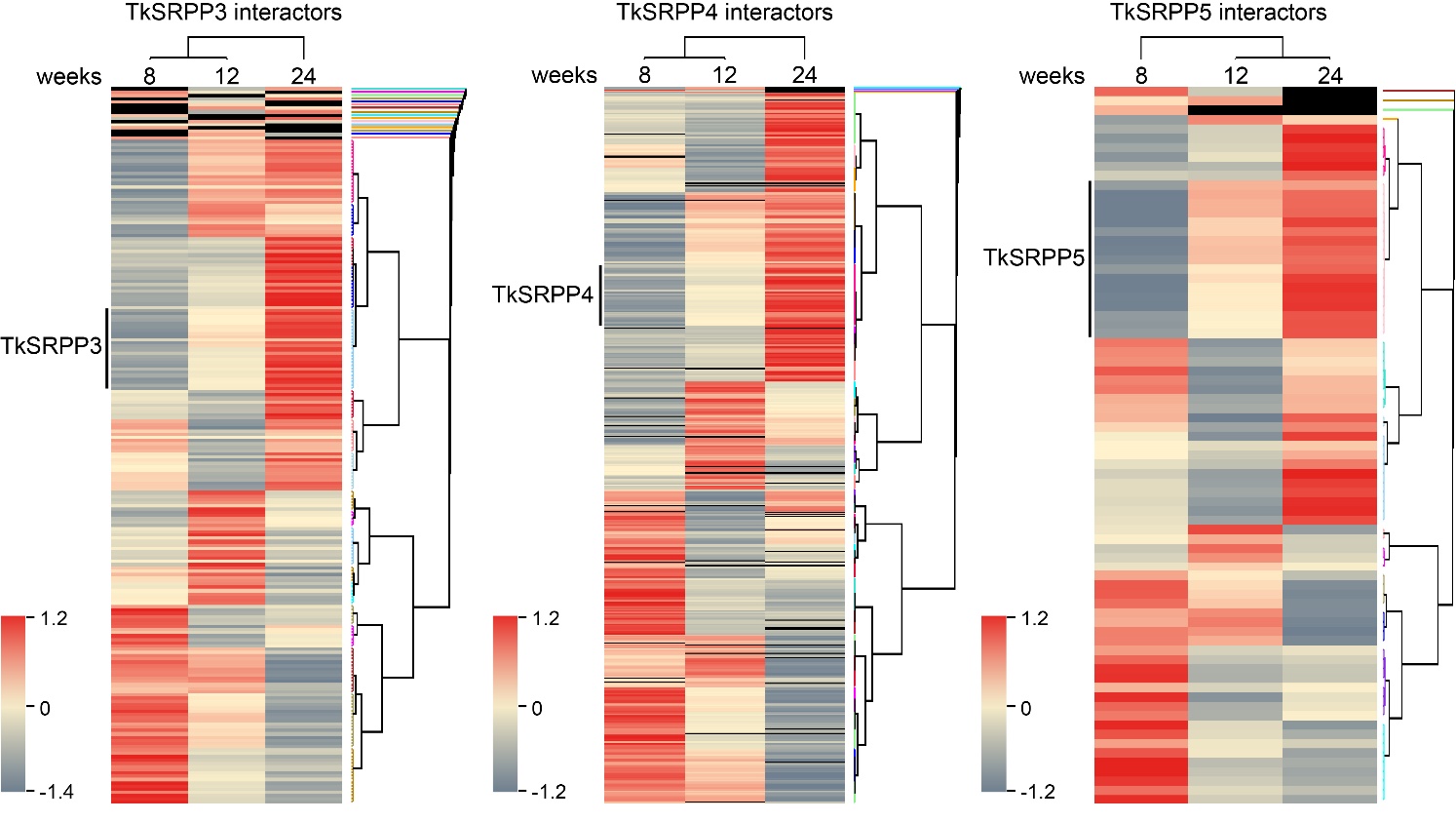


Supplementary Figure S4. Heat maps of TkSRPP3–5 interactor abundances in wild-type *T. koksaghyz* roots over time. Protein abundance data were obtained from Benninghaus *et al.* (2020). Heat maps were generated based on Pearson correlation distance and display z-transformed log_2_(LFQ values). Missing values are displayed in black. In each heat map, the cluster comprising the respective interacting TkSRPP is marked.


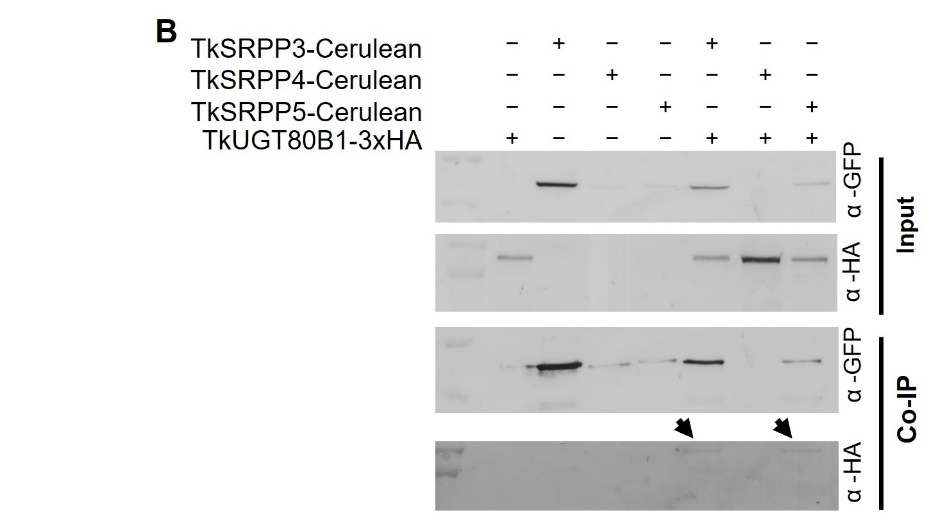

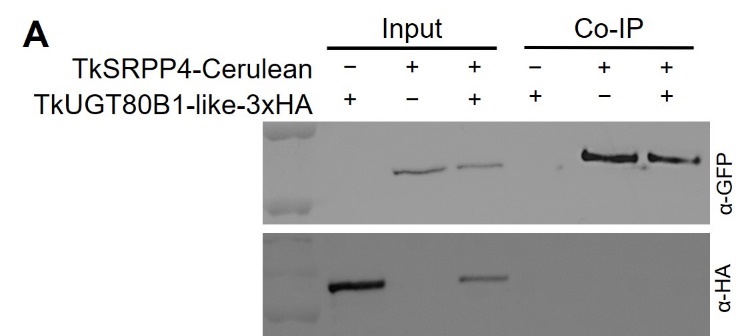


Figure S5: Co-Immunoprecipitation (Co-IP) assay with TkUGT80B1-3xHA. (A) Co-IP of TkUGT80B1-3xHA and TkSRPP4-Cerulean expressed in yeast at 20 °C. Pulldown of TkUGT80B1 with TkSRPP4 was not detectable in this setup. (B) Original western blot images of Co-IP of TkUGT80B1-3xHA and TkSRPP3­5-Cerulean expressed in yeast at 30 °C. TkSRPP4 expression was not detectable in this setup. Input samples and protein detection after immunoprecipitation with an α-GFP antibody are shown.


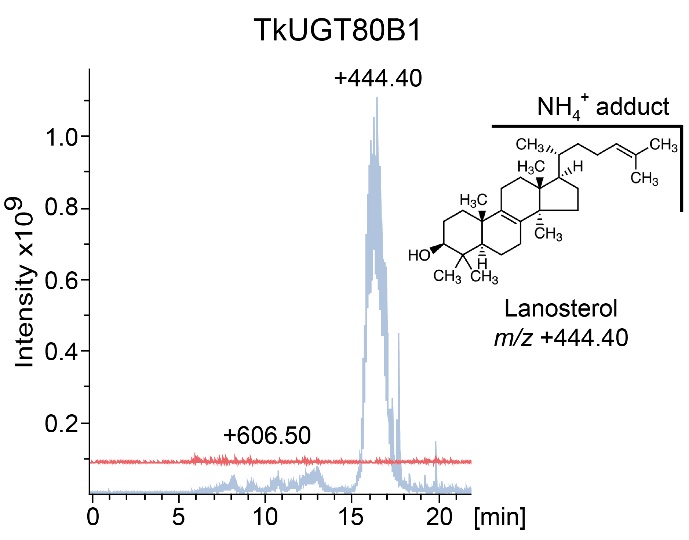


Figure S6. LC-MS chromatogram of extracts from yeast metabolically engineered for increased triterpenoid production (Bröker et al., 2018) expressing *TkUGT80B1* without *TkLup*. This yeast strain served as control compared to a strain expressing *TkLup* and *TkUGT80B1*. Here, the *m/z* +444.40 value corresponds only to the lanosterol ammonium ion, which has the same mass as lupeol. No peak for *m/z* +606.50 corresponding to the ammonium ion of glucosylated lanosterol is present indicating TkUGT80B1 does not use lanosterol as a substrate.

## Supplementary Tables

Supplementary Table S1. Codon-optimized *TkSRPP3–5* sequences for cloning into pET23a(+).

| ***TkSRPP3* codon-optimized for *E. coli* with flanking BamHI and XhoI sites** |
| --- |
| GGATCCATGACCGATGCAGCAAGCGTTACCGAAGAACCGGAAAAAGTTCAGAGCCAAGAAGAAAAACTGAAACACCTGGATTTTGTGGAAGATGGTGTTAAACAGGCAGTTGGTTATGCAAGCAAAGCATATGATTACGCCAAAGATAAAAGCGGTCCGCTGAAACCGCATGTTGAAACCCTGGAAGGCACCATTAAACCGATTGTTGGTCCGGCATATGATAAATTTCAGGATGCACATACCGGTGTGCTGAAATTTGTTGATCAGTTTGATGAAGTTATGCCTCCGGCAGTTAAAGATGCAACCACCACCGCACGTAGCCTGAGCACCAATGTTGCAAGCGAAGTTAAAAACAATGGTGTTCTGGGCACCGCAAAAGAACTGCTGGTTAAAATTGAACCGGTTGCCGAAGAATATGCCAGCAGCGCATGGAAAACCTTTAACTATGTTCCGTATGTTACCACCTTTGCAAAAGCAGTTGCACCGACCGCAAGCTATTATAGCGAAAAATACAATGAAACCGTTCAGCAGACCGCAGATGAAGGTTATAAAGTTAGCAGCTATCTGCCGCTGGTTCCGACCGATAAAATTGCACGTGTTTTTAGCATTCCGGAACCGGAATCAGCAGGTCCGGGTGGTGAAGCAGAAGATAAAGAAGTTCCTGGCGGAGGCGAAGGTGGCGAAGCACTCGAG |
| ***TkSRPP4* codon-optimized for *E. coli* with flanking BamHI and XhoI sites** |
| GGATCCATGGCAGATGTTGCACCGGTTACCGATGAACCGGAACAGAAAGTTCAGAGCGAAAAAGAAACCCTGAAACATCTGGGTTTTGTGGAAGAAGGTATTCAGCAGGTTGCAGGTTATGCAAGCAAAGTTTATGATTACGCCAAAGAAAATGCAGGTACACTGAAACCGGGTGTGGAAACCATTGAAACCACCGTTAAAACCTATGGTGGTCCGGCATATGATACCCTGAATGGTGTTCTGAAATTCGCCGATAATAAAGTTATTGCACAGCTGGATAGCGTTCTGCCTCCGAGCGTTAAAGAAACCGCAAAAAGCCTGAGCACCACCGTTGTTAGTGATGTTAAAAATGTTGGTGTGGTGGAAACCGTGAAAGAACTGCTGATTAAAATGGAACCGACCGTTGAAGATTATGCCAGCAGCGGTTGGAAAACCATTAGCGATCTGCCGCTGGTTGCAAAACTGGCAGCAGCAATTGCACCGCTGGCAACCTATATTACCGAAAAATACAATGGCACCGTTCAGCAGACCGCAGATGAAGGTTATAAAGTTAGCAGCTATCTGCCTCTGGTGCCGACCGATAAACTGGGTCGTATTTTTAGCACCAGCACCAAAGAAGATGAAGAGGTTCCGGGTGGTCTGGGTGGTGAAGAAGCAACCGAAGTTCCGCTCGAG |
| ***TkSRPP5* codon-optimized for *E. coli* with flanking BamHI and XhoI sites** |
| GGATCCATGGCAGATGCAGCAAGCGTTACCGATGAACCGCAGGTTCAGACCGAAGGTGAAAAACTGAAATATCTGGAATTTGTGGAAGAAGCAATTACCCAGGCAATTGATTATGCCAGCAAAGTGTATGAATTTGCCAAAGAAAAAAGCGGTCCGCTGAAACCGGGTGTGGAAACCATTGAAACCACCCTGAAAACCGTTGTTGGTCCGGCATATGAAAAATATCATGATGTTCCGGTTGTGGTGCTGAAATTTGTTGATCGTAAAGTTGATGAAAGCGTGACCCAGATTGATGGTGTTCTGCCTCCGATTGTTAAAGATGCAACCAAAGTTGGTGTTGTGGAAACGGCAAAAGAATTTCTGGAAAAAATTGATCCGGTGGCCGAAAAATATGCAAGCAGCGCTTGGAGCACCGTTAATCAGCTGCCGCTGGTTGCAAGCACCGTGAAAGCACTGACCCCGAGCGCAGCACTGGTTACAGAAAAATACAATCAGACGGTGAAAGAAAACGCAAGCTTTCTGCCTCTGGTTCCGACCGATAAAATTGCACGTGTTTTTAGCATTCCGGAAAAAGATGCAGAAAAACCGGAACCGGCAGTTGTTCCGGGTGGTGAAGAAGAAGCAGCCGAAGAGGTTGCCGGTGGTGGTGGCGCAGAACTCGAG |

Supplementary Table S2. Oligonucleotides used in this study.

| **Target** | **Primer** | **Sequence [5′→3′]** | **Primer efficiency for qPCR (%)** |
| --- | --- | --- | --- |
| *TkSRPP1* | TkSRPP1qRTfw | gccgataacgctgttcctgtt | 107.10% |
|  | TkSRPP1qRTrv | ggcttgctttgctgcttcttg |  |
| *TkSRPP2* | TkSRPP2qRTfw | cgatgctcctgttactaatcaacc | 98.20% |
|  | TkSRPP2qRTrv | catgaatcgccgccacttgaacaaa |  |
| *TkSRPP3* | TkSRPP3qRTfw | cgacgctgcttctgttactga | 99.10% |
|  | TkSRPP3qRTrv | ataaccaactgcttgctttactcc |  |
|  | TkSRPP3 NcoI fw | AAACCATGggcACCGACGCTGCTTC |  |
|  | TkSRPP3 XhoI rv | AAACTCGAGCTAAGCTTCTCCCCCTTC |  |
|  | TkSRPP3ns XhoI rv | AAACTCGAGTAAGCTTCTCCCCCTTC |  |
|  | TkSRPP3 fwd seq | AAAAGAAAAGGTTCAGAGCCAAG |  |
| *TkSRPP4* | TkSRPP4 qPCR fw 1 | caattgcacctctagcaac | 100.0% |
|  | TkSRPP4 qPCR rv | catcttcctttgtagaagtgc |  |
|  | TkSRPP4 NcoI fw | AAACCATGGCCGATGTTGCACC |  |
|  | TkSRPP4 XhoI rv | ATACTCGAGTTAAGGAACCTCCGTAGC |  |
|  | TkSRPP4ns XhoI rv | AAACTCGAGTAAGGAACCTCCGTAGC |  |
|  | TkSRPP4 rv seq | AAATTAAGGAACCTCCGTAGCTTC |  |
| *TkSRPP5* | TkSRPP5qRTfw | gatgaaccacaggtccagac | 97.0% |
|  | TkSRPP5qRTrv | ccttagcaaactcatatacttttgatgc |  |
|  | TkSRPP5 NcoI fw | AAACCATGGCCGACGCTGCTTC |  |
|  | TkSRPP5 XhoI rv | TTTCTCGAGTTACTCCGCTCCACCACC |  |
|  | TkSRPP5ns XhoI rv | AAACTCGAGTACTCCGCTCCACCACC |  |
|  | TkSRPP5 rv seq | AAATTACTCCGCTCCACCACCAC |  |
| *TkSRPP6* | TkSRPP6qPCRfw3 | gaagccaatcaagcgactg | 105.5% |
|  | TkSRPP6qPCRrv3 | catgaaacctctcgcaaacc |  |
|  | TkSRPP6 BamHI fw | AAAggatccATGGCGGAATCTGAAGCC |  |
|  | TkSRPP6 XhoI rv | AAActcgagTGTATCAACATGAATAGCTTG |  |
| *TkSRPP7* | TkSRPP7 qPCR fw | tgttgtgggccctgctt | 98.0% |
|  | TkSRPP7 qPCR rv | tgtacgcggttttggct |  |
| *TkRP* | TkRP qPCR fw | cgtcgatctcaaggatgttgtc | 95.7% |
|  | TkRP qPCR rv | ggagctttgagaagaaccaacg |  |
| *TkEf1α* | TkEF1α qPCR fw | cgagagattcgagaaggaagc | 104.8% |
|  | TkEF1α qPCR rv | ctgtgcagtagtacttggtgg |  |
| *TkUGT80B1* | TkUGT80B1 qPCR fw 1b | ccatcatcgcaaatcctccc | 101.5% |
|  | TkUGT80B1 qPCR rv 1 | gtgctaagggggaagggaactc |  |
|  | Contig16188 KpnI fw | AAAggtacctcATGACCGAAAAATGGTGGAC |  |
|  | utg4564.7 BamHI fw | aaaGGATCCtaATGGATAGTGATATGATTAGGGAG |  |
|  | Contig16188 NotI rv | AAAgcggccgcTCAAGATGTACCACAGCC |  |
|  | Contig16188 ns NotI rv | AAAgcggccgcCAAGATGTACCACAGCC |  |
|  | TkUGT80B1 1256 rv | GGTTGATATTTTGAAGCAAGATTC |  |
|  | TkUGT80B1 1130 fw | GTTCAATTTCTCATTTTCCAAC |  |
| *TkGAPDH* | TkGAPDH fw | cttcagagagatgatgttgacc |  |
|  | TkGAPDH rv | cttccacctctccagtcctt |  |
|  | M13 rv | CAGGAAACAGCTATGAC |  |
|  | attR BglII fw | agaagaAGATCTcAacaagtttgtacaaaaaagctg |  |
|  | attR SpeI fw | agaagaactagtacaagtttgtacaaaaaagctg |  |
|  | attR AgeI rv | agaagaACCGGTaccactttgtacaagaaagc |  |
|  | 35S Prom fw | ATCTCCACTGACGTAAGG |  |
|  | 35S Term rv | TGCTCAACACATGAGCGAAACC |  |
|  | Cerulean seq rv | GTATCTTGCAAAGCATTGTACTC |  |
|  | eYFPrevscreen | agcttgccgtaggtggc |  |
|  | mEmerald screen rv | AAGTAGTGACAAGTGTTGG |  |
|  | PMet17 seq fw | GTCTTTTCATCTACTATTTCC |  |
|  | Cub seq rv | GTAATCAGACAGCGTTCTACC |  |
|  | Nua/Nui Seq fw | CAAGACTTTGACCGGTAAAACC |  |
|  | pAGD fw | ctagaactagtggatcccccatc |  |
|  | AtLEC2 NcoI fwd | AAACCATGGATAACTTCTTACCCTTTC |  |
|  | AtLEC2 NotI rev | AAAGCGGCCGCTCACCACCACCTCAAAGTC |  |

Supplementary Table S3. Overview of TkSRPPs analyzed in this study. Corresponding IDs from the two published *T. koksaghyz* genomes and comparison with nomenclatures of recently published studies.

| **Annotation in this study** | **Annotation Lin *et al.*, 2018** | **Gene ID Lin *et al.*, 2018** | **Annotation Lin *et al.*, 2022** | **Gene ID Lin *et al.*, 2022** | **GWHT ID Lin *et al.*, 2022** | **GWHG ID Lin *et al.*, 2022** | **Annotation He *et al.*, 2024** | **Annotation Wu *et al.*, 2024** |
| --- | --- | --- | --- | --- | --- | --- | --- | --- |
| TkSRPP1a | TkSRPP4 | evm.model.utg16048.6 | TkSRPP5 | TkA04G477570 | GWHTBCHF027828 | GWHGBCHF026520 | TkSRPP4 | TkSRPP4 |
| TkSRPP1a1 | TkSRPP4 | evm.model.utg16048.6 | TkSRPP11 | TkA04G488020 |  | GWHGBCHF026616 | TkSRPP10 | TkSRP4 |
| TkSRPP1b | TkSRPP3 | evm.model.utg8404.10 | TkSRPP6 | TkA04G477840.1TkA04G477840.2TkA04G477840.3 | GWHTBCHF027829 GWHTBCHF027830 GWHTBCHF027831 | GWHGBCHF026521 | TkSRPP5 | TkSRPP4 |
| TkSRPP1b1 | TkSRPP3 | evm.model.utg8404.10 | TkSRPP10 | TkA04G487970 | GWHTBCHF027939 | GWHGBCHF026615 | TkSRPP9 | TkSRPP4 |
| TkSRPP1c | TkSRPP4 | evm.model.utg16048.6 | TkSRPP7 | TkA04G478010 | GWHTBCHF027832 | GWHGBCHF026522 | TkSRPP6 | TkSRPP4 |
| TkSRPP1c1 | - | - | - | GWHBCHF00000005:123,296,947-123,297,889 | - | - | - | - |
| TkSRPP2a | TkSRPP2 | evm.model.utg16048.3 | - | GWHBCHF00000005:120,751,923-120,750,745 | - | - | - | TkSRPP2 |
| TkSRPP2b | TkSRPP2 | evm.model.utg16048.3 | - | TkA04G478030 | GWHTBCHF027833 | GWHGBCHF026523 | - | TkSRPP2 |
| TkSRPP3 | TkSRPP5 | evm.model.utg19387.14 | TkSRPP4 | TkA04G477110 | GWHTBCHF027826 | GWHGBCHF026517 | TkSRPP3 | TkSRPP5 |
| TkSRPP4 | TkSRPP9 | evm.model.utg19387.11 | TkSRPP3 | TkA04G477020 | GWHTBCHF027825 | GWHGBCHF026516 | TkSRPP2 | TkSRPP9 |
| TkSRPP5 | TkSRPP6 | evm.model.utg19387.8 | TkSRPP2 | TkA04G476990 | - | GWHGBCHF026515 | - | TkSRPP6 |
| TkSRPP6 | TkSRPP7 | evm.model.utg2059.21 | TkSRPP1 | TkA04G007290 | GWHTBCHF022706 | GWHGBCHF021617 | TkSRPP1 | TkSRPP7 |
| TkSRPP7a | - | evm.model.utg16048.5 | TkSRPP8 | TkA04G478030 | GWHTBCHF027833 | GWHGBCHF026523 | TkSRPP7 | TkSRPP1 |
| TkSRPP7b | - | evm.model.utg16048.5 | TkSRPP9 | TkA04G487760 | GWHTBCHF027937 | GWHGBCHF026613 | TkSRPP8 | TkSRPP1 |
| TkSRPP8a | TkSRPP8 | evm.model.utg7314.20 | - | GWHBCHF00000004: 28,516,148- 28,515,492 |  |  | - | - |
| TkSRPP8b | - | - | - | GWHBCHF00000004: 28,519,409- 28,518,753 |  |  |  |  |
| TkSRPP8c | - | - | - | GWHBCHF00000004: 28,522,668- 28,522,012 |  |  |  |  |

Supplementary Table S4. Enriched GO cellular components among proteins found in separated latex fractions.

|  | **GO ID** | **GO Term** | **# Background proteins** | **# Subset proteins** | **# Expected subset proteins** | **p-value weight Fisher’s exact test** |
| --- | --- | --- | --- | --- | --- | --- |
| RP | GO:0009506 | plasmodesma | 216 | 204 | 184.41 | 8.00E-06 |
|  | GO:0005774 | vacuolar membrane | 172 | 163 | 146.84 | 0.00016 |
|  | GO:0005886 | plasma membrane | 384 | 349 | 327.84 | 0.00063 |
|  | GO:0005618 | cell wall | 111 | 105 | 94.77 | 0.00907 |
|  | GO:0005739 | mitochondrion | 238 | 222 | 203.19 | 0.01006 |
|  | GO:0005743 | mitochondrial inner membrane | 71 | 68 | 60.62 | 0.01077 |
|  | GO:0016020 | membrane | 754 | 687 | 643.72 | 0.01341 |
|  | GO:0016021 | integral component of membrane | 95 | 93 | 81.11 | 0.01605 |
|  | GO:0005730 | nucleolus | 106 | 97 | 90.5 | 0.038 |
|  | GO:0098796 | membrane protein complex | 119 | 111 | 101.6 | 0.04441 |
| IP | GO:0005829 | cytosol | 661 | 653 | 628.01 | 4.20E-09 |
|  | GO:0005737 | cytoplasm | 1519 | 1442 | 1443.18 | 0.015 |
|  | GO:0005634 | nucleus | 484 | 473 | 459.84 | 0.019 |
| PP | GO:0009506 | plasmodesma | 216 | 206 | 186.67 | 4.60E-06 |
|  | GO:0005774 | vacuolar membrane | 172 | 165 | 148.64 | 0.00013 |
|  | GO:0005886 | plasma membrane | 384 | 354 | 331.85 | 0.00019 |
|  | GO:0005783 | endoplasmic reticulum | 145 | 138 | 125.31 | 0.00353 |
|  | GO:0005618 | cell wall | 111 | 106 | 95.93 | 0.00604 |
|  | GO:0005739 | mitochondrion | 238 | 228 | 205.68 | 0.00845 |
|  | GO:0005794 | Golgi apparatus | 154 | 141 | 133.09 | 0.00956 |
|  | GO:0005743 | mitochondrial inner membrane | 71 | 71 | 61.36 | 0.01446 |
|  | GO:0016021 | integral component of membrane | 95 | 94 | 82.1 | 0.02174 |
|  | GO:0016020 | membrane | 754 | 699 | 651.6 | 0.03762 |
|  | GO:0005759 | mitochondrial matrix | 34 | 33 | 29.38 | 0.04292 |

Supplementary Table S5. List of *cis*-acting regulatory elements found within the first 1 kb upstream of the *TkSRPP6* and *TkUGT80B1* genes. Motifs were identified using NSITE-PL.

|  | **Gene and organism** | **Transcription factor binding site** | **Binding factor** | **Position and sequence** |
| --- | --- | --- | --- | --- |
| TkSRPP6  (27 motifs) | *rbcS-3.6*  *Pisum sativum* | ST-1 (2) | AT-1 | - strand 533-521  AAgTATTTTTATT |
|  | *Aux28*  *Glycine max* | B1-core | SGBF-1/2 | - strand 802-793  TaCACGTGTC |
|  | *rbcS2*  *Lycopersicon esculentum* | 2&3 U | Unknown nuclear factor | - strand 812-793  ttGAGaTGCTTACACGTGTC |
|  | *A121*  *Hordeum vulgare* | M1 | DOF | - strand 922-913  tAACGGCAAC |
|  | *patatin*  *Solanum tuberosum* | G-Box | GBF | - strand 802-793  TaCACGTGTC |
|  | *Synthetic oligonucleotides*  *Arabidopsis thaliana* | GBF1 BS3 | GBF1 | + strand 794-801  ACACGTGT  - strand 901-794  ACACGTGT |
|  | *CHS2*  *P. sativum* | Box II | Unknown transcription factor | - strand 396-385  TCAAtAAACTCA |
|  | *ABI5*  *A. thaliana* | CE1-like element | ABI4 | - strand 499-493  GCCACCG |
|  | *COX5b-2* (At1g80230)  *A. thaliana* | G-Box | Unknown nuclear factor | + strand 793-802  GACACGTGTa  - strand 802-793  tACACGTGTC |
|  | *Synthetic oligonucleotides*  *A. thaliana* | G-Box | AtMYC2/JIN1 (ZBF1) | + strand 792-803  TGACACGTGtaA  - strand 803-792  TtACACGTGtCA |
|  | *BRI1*  *Oryza sativa* | E-Box (BRI1, P2_1) | RAVL1 | + strand 178-187  TACAAATGAT |
|  | *Synthetic oligonucleotides*  *A. thaliana* | bZIP28 BS | bZIP28 | + strand 793-800  GACACGTG |
|  | *Pks1*  *Phytophthora infestans* | CATTTGTTTT motif | Unknown nuclear factor | - strand 696-687  CATTTGTTTT |
|  | *ERF11*  *A. thaliana* | G-Box | OsHsfA2c; OsHsfA9 | - strand 803+792  ttACACGTGTCA |
|  | *QPT2*  *Nicotiana tabacum* | G-Box (QPT2) | NtMYC2 | + strand 794-801  ACACGTGT  - strand 801-794  ACACGTGT |
|  | *BDP 2-273*  *O. sativa* | CRE45-48 | Unknown nuclear factor | + strand 492-498  GCGGTGG |
|  | *ANAC019*  *A. thaliana* | PIF7 BS1 | PIF7 | + strand 793-802  GACACGTGtA |
|  | *ANAC019*  *A. thaliana* | PIF7 BS2 | PIF7 | - strand 802-793  tACACGTGTC |
|  | *ANAC055*  *A. thaliana* | PIF7 BS1 | PIF7 | - strand 802-793  TACACGTGTc |
|  | *ANAC055*  *A. thaliana* | PIF7 BS2 | PIF7 | + strand 793-802  GACACGTGtA |
|  | *ANAC072*  *A. thaliana* | PIF7 BS1 | PIF7 | + strand 793-802  GACACGTGTa |
|  | *ANAC072*  *A. thaliana* | PIF7 BS2 | CBF1; CBF2; CBF3; CBF4 | + strand 793-802  GACACGTGtA |
|  | *MIR168a*  *A. thaliana* | ABRE5 | ABF1; ABF2; ABF3; ABF4 | - strand 800-793  CACGTGTC |
| TkUGT80B1  (15 motifs) | *prxC2*  *Armoracia lapathifolia* | Box 3 | Unknown nuclear factor | - strand 691-680  AGAATTATTAAA |
|  | *hcbt2*  *Dianthus caryophyllus* | ERE2 | Unknown nuclear factor | + strand 167-177  TTGGTCAATTt |
|  | *α-Amy2*  *Avena fatua* | Box 2 | Unknown nuclear factor | - strand 431-417 TGAaTTGACCGctAT |
|  | *rbcS3A*  *L. esculentum* | Box II EE1 | Unknown nuclear factor | - strand 906-898  GCTTAATTA |
|  | *prxC2*  *N. tabacum* | Box 3 | Unknown nuclear factor | - strand 691-680  AGAATTATTAAA |
|  | *STK*  *A. thaliana* | GA-1 | BPC1 | + strand 99-107  AGAAAGAAA |
|  | *hcbt2*  *D. caryophyllus* | ERE2 | Unknown nuclear factor | - strand 177-167 aAATTGACCAA |
|  | *hcbt2*  *D. caryophyllus* | ERE3 | Unknown nuclear factor | - strand 177-167 aAATTGACCAA |
|  | *SIRK/FRK1* (At2g19190)  *A. thaliana* | AtSIRKp (WRKY11) BS2 | WRKY11 | - strand 177-165  aAaTTGACCAATA |
|  | *AtCMPG1* (At3g02840)  *A. thaliana* | WRKY11 BS | WRKY11 | - strand 429-418  AATTGACCGcTa |
|  | *Hsp18.1-CI*  *A. thaliana* | TATA-HSE module M5 | HsfA2 | - strand 628-613  TCGATTCAAGAATCCC |
|  | *ABF4*  *A. thaliana* | W-Box 3 | WRKY40 | - strand 792-781  AAAAGTCAaCAA |
|  | *BRI1*  *O. sativa* | E-Box (BRI1, P1_1) | RAVL1 | + strand 44-53  tGCACATGCC |
|  | *ACS4*  *L. esculentum* | CArG (LeACS4-b) | RIN | + strand 605-614  CAATTTTGGG |
|  | *Synthetic oligonucleotides*  *A. thaliana* | NTL8 BS | NTL8 | - strand 599-590  GTTTCCTTTT |

For **Supplementary Table S6** please see attached Excel File.

Supplementary Table S7. List of selected interactors related to NR and isoprenoid synthesis.

|  |  | **EVM ID** | **Protein name** | **DAP *TkCPTL1*-RNAi** | **Latex** | | **RP** | | **IP** | | **PP** | |
| --- | --- | --- | --- | --- | --- | --- | --- | --- | --- | --- | --- | --- |
|  |  |  |  |  | **–log q‑value** | **log_2_FC** | **–log q‑value** | **log_2_FC** | **–log q‑value** | **log_2_FC** | **–log q‑value** | **log_2_FC** |
| **TkSRPP3** | **REF family** | evm.model.utg8404.10 | TkSRPP1 |  | 1.69 | 1.98 |  |  |  |  |  |  |
|  |  | evm.model.utg19387.8 | TkSRPP5 |  | 1.34 | 1.05 |  |  | 2.00 | 2.72 |  |  |
|  |  | evm.model.utg2059.21 | TkSRPP6 |  |  |  | 2.00 | 4.67 | 1.61 | 4.96 |  |  |
|  |  | evm.model.utg16048.5 | TkSRPP7 |  | 2.18 | 2.28 |  |  | 1.42 | 2.07 |  |  |
|  |  | evm.model.utg9849.3 | TkREF |  | 1.51 | 1.94 |  |  | 1.63 | 1.89 |  |  |
|  | **MVA pathway** | evm.model.utg7214.3 | TkMVAK10 |  | 1.69 | 1.19 |  |  |  |  |  |  |
|  |  | evm.model.utg1827.11 | TkPMVK3 |  | 1.38 | 3.34 |  |  |  |  |  |  |
|  | **NR synthesis** | evm.model.utg11341.6 | TkCPT1 | down | 2.47 | 2.28 |  |  | 1.72 | 1.25 |  |  |
|  | **Terpenoid synthesis** | evm.model.utg489.7 | TkOSC1 |  | 1.45 | 3.86 |  |  |  |  |  |  |
| **TkSRPP4** | **REF family** | evm.model.utg16048.3 | TkSRPP2 |  | 1.77 | 1.21 |  |  |  |  | 1.71 | 1.81 |
|  |  | evm.model.utg2059.21 | TkSRPP6 |  | 1.35 | 1.09 |  |  |  |  |  |  |
|  |  | evm.model.utg16048.5 | TkSRPP7 |  | 1.63 | 1.04 |  |  |  |  |  |  |
|  | **MVA pathway** | evm.model.utg20332.7 | TkACLA1 |  |  |  | 2.20 | 1.54 |  |  | 1.91 | 2.55 |
|  |  | evm.model.utg10104.22 | TkHMGR1 | down |  |  |  |  | 3.30 | 2.68 |  |  |
|  |  | evm.model.utg12000.19 | TkMVAK1 |  |  |  | 2.24 | 3.20 |  |  |  |  |
|  |  | evm.model.utg1827.11 | TkPMVK3 |  | 1.40 | 1.97 |  |  |  |  |  |  |
|  | **MEP pathway** | evm.model.utg6938.1 | TkDXS8 |  |  |  |  |  | 3.30 | 6.12 |  |  |
|  |  | evm.model.utg341.5 | TkHDS1 |  |  |  | 1.37 | 1.73 |  |  |  |  |
|  | **Terpenoid synthesis** | evm.model.utg4319.82 | TkFPS1 |  |  |  |  |  | 3.30 | 2.11 |  |  |
|  |  | evm.model.utg9445.19 | TkSQS1 |  |  |  |  |  | 3.30 | 2.79 |  |  |
|  |  | evm.model.utg16440.3 | TkSQE1 | up |  |  |  |  | 1.89 | 1.43 |  |  |
|  |  | evm.model.utg31094.4 | TkOSC5 |  |  |  |  |  |  |  | 2.06 | 4.85 |
|  |  | evm.model.utg24682.2 | TkGAO | up |  |  |  |  | 2.85 | 4.05 |  |  |
|  |  | evm.model.utg1018.48 | TkCOS1 |  |  |  |  |  | 3.28 | 5.15 |  |  |
| **TkSRPP5** | **REF family** | evm.model.utg16048.3 | TkSRPP2 |  | 2.52 | 2.12 | 2.30 | 1.28 | 2.11 | 2.31 |  |  |
|  |  | evm.model.utg2059.21 | TkSRPP6 |  |  |  |  |  | 2.49 | 2.24 |  |  |
|  |  | evm.model.utg16048.5 | TkSRPP7 |  | 1.83 | 1.42 |  |  | 1.95 | 2.56 |  |  |
|  | **MEP pathway** | evm.model.utg341.5 | TkHDS1 |  | 2.17 | 1.68 |  |  |  |  |  |  |
|  | **Terpenoid synthesis** | evm.model.utg31094.4 | TkOSC5 |  |  |  | 1.45 | 1.35 |  |  |  |  |
|  |  | evm.model.utg6273.3 | TkDDS |  |  |  | 2.3 | 3.51 |  |  |  |  |
